# Transcriptional activation during cell reprogramming correlates with the formation of 3D open chromatin hubs

**DOI:** 10.1038/s41467-020-16396-1

**Published:** 2020-05-22

**Authors:** Marco Di Stefano, Ralph Stadhouders, Irene Farabella, David Castillo, François Serra, Thomas Graf, Marc A. Marti-Renom

**Affiliations:** 1 CNAG-CRG, Centre for Genomic Regulation (CRG), Barcelona Institute of Science and Technology (BIST), Baldiri i Reixac 4, 08028 Barcelona, Spain; 2 Centre for Genomic Regulation (CRG), Barcelona Institute of Science and Technology (BIST), Dr. Aiguader 88, 08003 Barcelona, Spain; 3 Universitat Pompeu Fabra (UPF), 08002 Barcelona, Spain; 4 ICREA, Pg. Lluís Companys 23, 08010 Barcelona, Spain; 5 Present Address: Department of Pulmonary Medicine and Department of Cell Biology, Erasmus MC, Rotterdam, the Netherlands; 6 Present Address: Computational Biology Group—Barcelona Supercomputing Center (BSC), 08034 Barcelona, Spain

**Keywords:** Computational models, Genome informatics

## Abstract

Chromosome structure is a crucial regulatory factor for a wide range of nuclear processes. Chromosome conformation capture (3C)-based experiments combined with computational modelling are pivotal for unveiling 3D chromosome structure. Here, we introduce TADdyn, a tool that integrates time-course 3C data, restraint-based modelling, and molecular dynamics to simulate the structural rearrangements of genomic loci in a completely data-driven way. We apply TADdyn on in situ Hi-C time-course experiments studying the reprogramming of murine B cells to pluripotent cells, and characterize the structural rearrangements that take place upon changes in the transcriptional state of 21 genomic loci of diverse expression dynamics. By measuring various structural and dynamical properties, we find that during gene activation, the transcription starting site contacts with open and active regions in 3D chromatin domains. We propose that these 3D hubs of open and active chromatin may constitute a general feature to trigger and maintain gene transcription.

## Abstract (web summary)

Regulation of chromosome structure plays essential roles in many nuclear processes. Here, the authors present TADdyn, a tool that integrates time-course 3C data, restraint-based modelling, and molecular dynamics to simulate the structural rearrangements of genomic loci and find that during gene activation, transcription starting sites contact with open chromatin regions into active physical domains.

## Introduction

The three-dimensional (3D) structure of the genome has been shown to modulate transcriptional regulation^1–3^ and to play a role in cancer and developmental abnormalities^4^. In the effort of characterizing 3D genome structures, chromosome conformation capture (3C)-based experiments^5^ allow to capture a single snapshot of the genome conformation at a given time. A plethora of theoretical approaches have been developed to take advantage of 3C-based experimental data and model genome spatial organization. Restraint-based modelling approaches^6^ take 3C-based contact frequencies as input and employ ad hoc conversions to spatial distances for determining 3D genome structure^7–12^. This approach has provided valuable insights into the structural organization of chromosomal regions in various organisms^13^. Complementary, thermodynamics-based approaches^14–22^ use physics-based principles to test specific interactions or interaction mechanisms to explain the molecular origins of the contact patterns obtained in 3C-based experiments. Together, these theoretical strategies provide insights into chromatin conformation^16,17,23,24^ and the possible mechanisms that form chromosome territories^18^, compartments^19^ and topologically associating domains (TADs)^20,22,25,26^.

Decreased sequencing costs, together with more refined experimental protocols, has permitted performing 3C-based time-resolved experiments to monitor genome conformation dynamics of biological processes at high resolution. For example, High-throughput chromosome conformation capture (Hi–C) experiments have been applied to study the dynamics of nuclear organization during mitosis^27,28^ or meiosis^29–31^, during hormone treatment^32^ and during induced neural or adipose cells differentiations^33,34^ or cell reprogramming^35^. However, none of the computational strategies developed so far can take full advantage of these time-series datasets. Hence, approaches specifically designed for the simulation of time-dependent conformational changes (4D) are urgently needed.

To fill this gap, we introduce TADdyn, a computational method allowing to model 3D structural transitions of chromatin using time-resolved Hi–C datasets. We combine in TADdyn a physics-based model of chromatin fiber^18,36^ with dynamic restraint-based modelling. For any genomic locus, this integrated strategy allows for simulating a plausible 4D trajectory that is data-driven and at the same time satisfies basic physical properties of the chromatin fiber.

The potential of TADdyn to provide insights beyond the Hi–C datasets is highlighted by the simulation of 21 loci of the mouse genome during cell reprogramming of pre-B lymphocytes into pluripotent stem cells (PSCs)^35^. By measuring structural and dynamical properties from the simulations, we characterize the interplay between 3D structure and gene transcription at an extent unreachable from the experimental datasets alone. Interestingly, we find that transcription starting sites (TSS) of simulated loci embed into in a cage-like structure that favors contacts with open and active regions located (even) several kilo-bases (kb) away from the gene promoter. Hence, TADdyn simulations are compatible with the formation of 3D hubs^37^ as a general mechanism to modulate gene transcription.

## Results

### The TADdyn modelling strategy

**Fig. 1:**
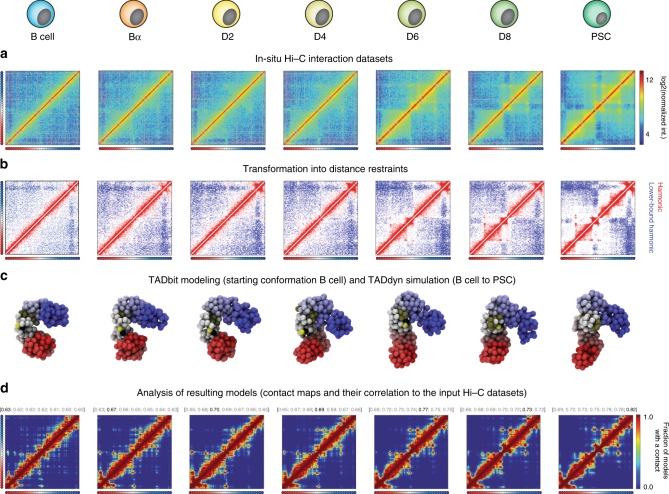
The TADdyn key steps for simulating 4D dynamic changes in a locus. The shown example corresponds to the reprogramming dynamics for the *Sox2* locus from B cells to PSC. **a** Data collection. In situ normalized Hi–C interaction matrices^35^ for the region of 1.5 Mb centered around the *Sox2* promoter. **b** Distance restraints definition. Both LowerBound- (blue) and Harmonic (red) spatial restraints are obtained by filtering the Hi–C interaction maps using the optimal triplet of TADbit parameters (“Methods”). **c** TADdyn steered dynamics runs. The simulated regions are represented as polymers made of spherical particles each a 5 kb-bin of the input Hi–C matrix. Particles are colored from red (first particle) to blue (last particle of the modeled region). The TSS of the locus of interest is represented by a black particle and the promoter by green ones. The models for all the stages (that is, from B to PSC) were dynamically built by TADdyn. **d** Contact maps (dcutoff = 200 nm) of models during the simulation were used to assess their accuracy by means of its Spearman correlation coefficients (SCCs) with the Hi–C input matrices. The SCCs of the contact maps with the seven Hi–C input matrices are shown with the coefficient in bold black letters corresponding to the time point of the column. As our previous modelling benchmark indicates^41^, all the SCCs > 0.60, that are obtained between models and Hi–C maps at corresponding cell stages, are indicative of good models. In c, d, only the models and the contact maps at the correspondent experimental time-points are shown, but TADdyn allows to visualize the entire dynamics filling the blanks between stages as shown in the Supplementary Movies 1–21.

TADdyn is based on the following methodological steps (“Methods” and Fig. 1): (i) collection of experimental data, (ii) representation of selected chromatin regions using a bead-spring polymer model, (iii) conversion of experimental data into time-dependent restraints, (iv) application of steered molecular dynamics to simulate the adaptation of chromatin models to satisfy the imposed restraints, and (v) analysis of the conformation dynamics. As discussed below, each of these steps constitutes per se an extension of all the existing restraint-based strategies for chromosome modelling and, in particular, of TADbit^38^, a modelling tools previously developed in our lab.

We applied TADdyn to a previously published in situ Hi–C interaction time-series dataset (GEO accession number GSE96611). We could use at once restraint-based modelling for seven distinct time-points of in situ Hi–C experiments during *C/EBPα* priming followed by *Oct4*, *Sox2*, *Klf4* and *Myc* (OSKM)-induced reprogramming of B cells to PSCs^35^. To collect statistics on distinct expression dynamics, we focused on 20 different ~2 mega-bases (Mb) regions of the mouse genome encompassing a total of 21 different loci (Supplementary Data 1). The selected genes are representative of different time-dependent patterns of transcriptional activity (Supplementary Fig. 1 and “Methods”), which allowed us to study how various different transcription dynamics interplay with changes in the 3D genomic organization. Overall, we analyzed seven continuously active loci (*Hsp90ab1, Ppia*, *Rad23a, Rad23b, Rpl41, Rps14*, and *Rps26*) and 5 completely silent ones (*Neurod6*, *Olfr1022*, *Olfr33*, *Rergl*, and *Rnu7*). Additionally, we studied 9 loci of interest with varying transcriptional dynamics, such as: the early activated *Tet2*, the late activated *Sox2* and *Nanog* reprogramming genes, the transiently activated *Nos1ap* gene, the transiently silenced *Lmo7*, and the gradually silenced *Mmp3*, *Mmp12, C/EBPα*, *Ebf1* genes (Supplementary Data 1, Supplementary Figs. 2–22a and Supplementary Movies 1–21).

Next, Hi–C interaction matrices of all the regions at 5 kb resolution were converted into TADdyn time-dependent spatial restraints (“Methods” and Fig. 1a). Specifically, each region of interest was represented as a chain of spherical beads each spanning 50 nm in diameter and containing 5 kb of DNA. The chain features the general physical properties of the chromatin fiber: connectivity, excluded volume and (optionally) bending rigidity^36^ (“Methods”). At each experimental stage the Hi–C interaction matrix was converted into harmonic spatial restraints between pairs of particles (Fig. 1b). This conversion follows the simple, yet effective, rationale that pairs of particles with high Hi–C interaction can be restrained close in space, while poorly interacting particle pairs can be kept far apart^39^ (“Methods”). Differently from previous methods^40^, the parameters of each imposed pairwise harmonic restraint (the spring constant and the equilibrium distance) were linearly interpolated between the values at two consecutive experimental cell stages during the steered molecular dynamics simulations (“Methods” and Supplementary Fig. 23). The TADdyn dynamic restraints were designed to simulate smooth structural changes between models of consecutive experimental data points.

### TADdyn simulations are an accurate dynamic description of the input Hi–C data

To quantify the degree of agreement of the TADdyn 4D models to the time-series Hi–C interaction matrices, contact maps were calculated on an ensemble of 100 models obtained at each time step of the trajectories (Fig. 1d). Next, for each time-point the Spearman correlation coefficient (SCC) of the modeled contact map and the experimental Hi–C interaction matrices was computed as a measure of the agreement between the two matrices (Methods). For the 21 loci, the SCC ranged between 0.60 and 0.89 (Fig. 1d for *Sox2* simulations and Supplementary Figs. 2–22c for all other loci). From a previous benchmark study of restraint-based models^41^, we conclude that such SCC values are a good proxy of accurate reconstructions of genomes and genomic domains. Notably, at each cell stage, the Hi–C interaction map correlated best with the contacts maps at the corresponding time of the simulated reprogramming trajectories. These results show that for a diverse set of loci the simulated structures are a reliable 3D representation of two-dimensional Hi–C interaction matrices, which effectively reproduce the actual contact pattern at the correct time of the trajectory. Interestingly, these results, obtained from 100 trajectories, did not vary when the ensemble of simulated trajectories was extended to 500 or 1000 (Supplementary Fig. 24). This result is an indication of the robustness of TADdyn’s simulations, and suggests that 100 models are enough to obtain exhaustive statistics on the system and to draw meaningful conclusions on the simulated dynamics.

To test whether TADdyn is suited to model processes characterized by gradual chromatin structural transitions, we also performed two alternative set of simulations for the *Sox2* locus. We removed restraints from cell stages D2 (ΔD2 dynamics) and D6 (ΔD6 dynamics), and doubled the time duration (from 10 to 20 τ_LJ_) of the remaining transitions (Bα→D4 in ΔD2 and D4→D8 in ΔD6 simulations) to maintain the same total time per trajectory (60 τ_LJ_). These tests indicated that the deleted restraints marginally affected the overall dynamics of the locus. Specifically, the conformations expected to represent the missing cell stages along the trajectories still provided accurate models at the removed stage (SCC_D2_ = 0.68 and SCC_D6_ = 0.75 in ΔD2 and ΔD6 simulations, respectively). Additionally, in the case of the ΔD2 simulation, the Hi–C interactions map at D2 correlated best with the contact map computed on the models predicting the D2 stage, while, for the ΔD6, the SCC between Hi–C in D6 correlated slightly better with the models in PSC than in D6 (SCC_PSC_ = 0.76 vs. SCC_D6_ = 0.75). These results suggest that the reprogramming dynamics, simulated in this work, are dominated by smooth and gradual chromatin rearrangements that can be effectively and robustly simulated using TADdyn.

For clarity, we present the details of the simulations for three loci, *Sox2* (as a late activated locus), *Mmp12* (as a late repressed locus) and *Rad23a* (as a stably active locus) in the following sections. The cumulative analysis of the 21 genes is presented in the last paragraph of the Results section. All the results for all the simulated loci are presented in the Supplementary Figs. 2–22, and in the Supplementary Movies 1–21.

### Dynamic structural reorganization correlates with local transcriptional changes

**Fig. 2:**
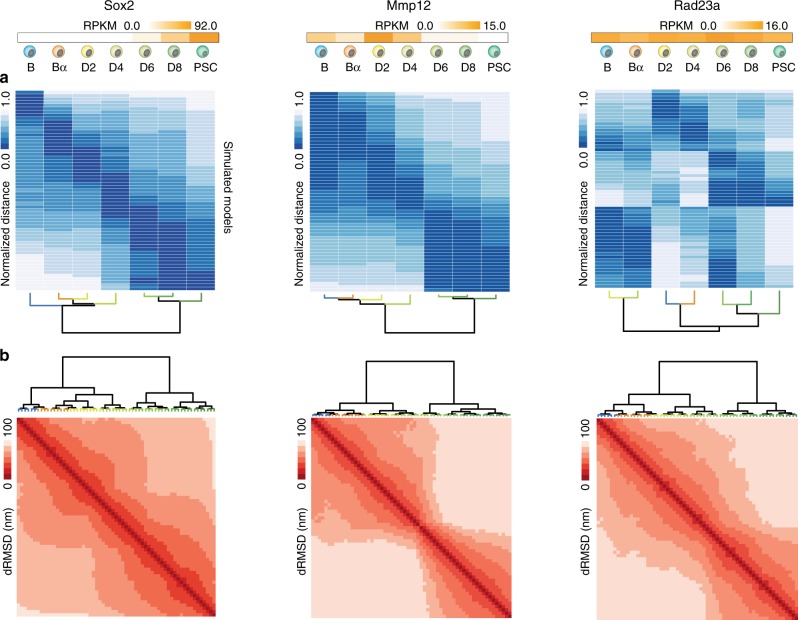
Dynamic structural chromatin reorganization linked to transcriptional activity. Top of the figure indicates expression level (RPKM) for each of the selected loci at each reprogramming stage^35^. **a** Heat-map of the normalized spearman rank correlations computed for each pair of 60 model contact maps (i.e. one map every 1-time step of the simulation) against each of the seven Hi–C interaction maps obtained during the reprogramming process. **b** Heat-map of the distance RMSD (dRMSD) between all models within a simulation. Each heat-map is accompanied by the corresponding dendrogram obtained from the clustering analysis (“Methods”).

To explore how the simulated models changed over time, we performed a hierarchical clustering analysis of the correlation matrix between the contact maps of the models and the input Hi–C interaction matrices. As a term of comparison, the same clustering analysis has been performed between the Hi–C interactions matrices including two replicates and the cumulative datasets (“Methods”, Fig. 2a, b and Supplementary Figs. 2–22d–f). Hi–C interaction matrices were grouped in well-separated clusters reflecting the expected changes in expression activity of each locus. This clustering indicates that the studied loci changed their topology along with their transcriptional activity. Interestingly, the bi-partite clustering is also reflected in the analysis of the Hi–C matrices (Supplementary Figs. 2–22e). Specifically, for 8 of the 21 simulated loci (that is *C/EBPα*, *Ebf1*, *Lmo7*, *Mmp12, Mmp3*, *Nos1ap, Sox2*, and *Tet2*) the active and inactive states of the locus were represented by two major clusters. For example, the first cluster of *Sox2* comprised the cell stages from B to D4 in which the locus is inactive (RPKM < 0.06), while the second cluster includes D6 to PSC when *Sox2* is active (RPKM > 8.0) (Fig. 2a). Similar observations were made for *Mmp12*. In contrast, *Rad23a* and other strongly expressed loci, such as *Ppia*, *Rpl41*, *Rps14*, *Rps26*, resulted in a less clear bipartite behavior likely because these loci remain in a constantly active state during reprogramming with relatively small fluctuations of expression. The *Rad23a* Hi–C datasets were clustered, for instance, in 3 (almost) equidistant clusters associating consecutive stages (D2-D4, B-Bα, and D6-D8-PSC) in a reshuffled order respect to the reprogramming dynamics. In almost all the highly expressed loci (*Rad23a*, *Ppia*, *Rpl41*, *Rps14*, *Rps26*), the clustering of the models’ correlations was mixed among the different cell stages. Interestingly, an analogous clustering analysis between the Hi–C datasets (two replicates and merged, “Methods”) reflects these stage mixing (*Ppia*, *Rad23a*, *Rpl41*, *Rps14*, *Rps26*, and *Hsp90ab1*). The silent loci (*Rnu7*, *Neurod6*, *Rergl*, *Olfr33*, and *Olfr1002*) clustered with different scenarios: most of these loci were clearly di- or tri-partite (*Olfr1002*, *Olfr33*, *Rergl*, and *Neurod6*) within the expected order of the reprogramming stages from B to PS, and only one, *Rnu7*, resulted in a completely reshuffled partition not reflecting the time course of the reprogramming nor the clusters of replicates of the Hi–C datasets. To further characterize the structural changes associated with variations in transcriptional activity, we performed a clustering analysis of the model structures based on the distance RMSD (dRMSD) values between pairs of 3D models (Methods). As for the matrix-based analysis, the dRMSD clustering reflects the presence of different folding states that correlated with the different transcriptional activities (Fig. 2b and Supplementary Figs. 2–22f).

### Time-dependent measures reveal locus-dependent structural dynamics

In our simulations (Supplementary Movies 1–21), for some of the studied loci (*Sox2*, *Mmp12*) we observed a “caging” effect of the transcription start site (TSS) at the time when the locus was transcriptionally active. To quantify this observation, we first calculated for each TSS the time-dependent changes in its 3D structural embedding as well its explored volume (Methods, Fig. 3 and Supplementary Figs. 2–22g, h). Notably, TADdyn simulations showed that, as cells reprogramed, the TSS of *Sox2* and *Mmp12* remained largely accessible to other particles during their less transcriptionally active phases (i.e. stages B-D4 for *Sox2* and D6-PSC for *Mmp12*) (Fig. 3a). During the most active stages, however, the TSS particle became embedded in the 3D model (the TSS embedding was between 0.8 and 0.95 at the PSC stage for *Sox2* and B-D4 for *Mmp12*) (Fig. 3a).

**Fig. 3:**
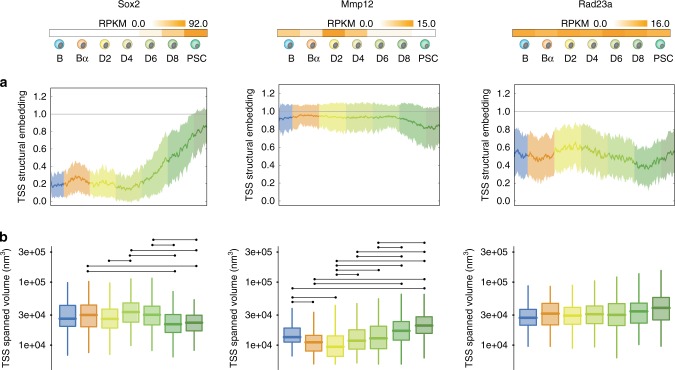
Gene activity correlates with TSS structural embedding and spanned volume. Top of the figure indicates expression level (RPKM) for each of the selected loci at each reprogramming stage^35^. **a** Dynamic structural embedding of the TSS particle. Each central line and the surrounding shadowed area represent respectively the average and the standard deviation interval of the profile over the 100 TADdyn replicates. **b** Volume explored by the TSS particle during the various stages of the reprogramming dynamics. The convex-hull values are represented as boxplots (*n* = 100 values for B and PSC and *n* = 200 values for the other cell stages) showing: central line, median; box limits, 75th and 25th percentiles; whiskers, 1.5× interquartile range. Outliers are not shown. Connecting lines between distributions indicate they are statistically different (*p* < 0.0001, two-sided Wilcoxon rank sum test). Exact significant p-values are: *Sox2* (*p*_Bα-D8_ = 3.7e-08_,_ *p*_Bα-PSC_ = 8.1e_-_06, *p*_D2-D4_ = 2.0e-06, *p*_D4-D8_ = 5.0e-14, *p*_D4-PSC_ = 7.8e-10, *p*_D6-D8_ = 6.9e-09, *p*_D6-PSC_ = 3.6e-06); *Mmp12* (*p*_B-Bα_ = 7.4e-08, *p*_B-D2_ = 4.0e-15, *p*_B-PSC_ = 2.6e-07, *p*_Bα-D8_ = 3.8e-17_,_ p_Bα-PSC_ = 7.5e_-_20, p_D2-D4_ = 3.3e-08, p_D2-D6_ = 3.5e-10, p_D2-D8_ = 5.4e-27, *p*_D2-PSC_ = 7.4e-26, *p*_D4-D8_ = 5.4e-11, *p*_D4-PSC_ = 4.7e-15, *p*_D6-D8_ = 1.8e-07, *p*_D6-PSC_ = 1.9e-11).

Next, we assessed whether the 3D embedding would also affect the dynamics of the TSS of different loci. For each simulation, we calculated the convex-hull of the TSS particle every 50 time-steps of the trajectory as a proxy of the volume explored (“Methods” and Fig. 3b). The results indicate that the TSS of *Sox2* explored a smaller volume in the D6-PSC stages, when the gene was transcriptionally active, compared to the volume explored in the B-D4 stages when the gene was transcriptionally silent. The TSS of *Mmp12* acquired increased mobility during reprogramming as its expression levels decreased (compare B-D4 and D6-PSC stages). Interestingly, the explored volume of the *Rad23a* TSS barely changed during the entire reprograming process. In fact, for several other loci the embedding and spanned volume profiles seem not to be related to transcriptional changes. For example, the embedding profiles of several continuously active loci, such as *Hsp90ab1* and *Ppia*, and silent genes, such as *Olfr1002* and *Rergl*, varied substantially along the simulations even if the loci were constantly active or inactive. These cases indicated that over the 21 simulated loci the dynamics of gene activation varied with no a unique general description in terms of embedding and explored volume. Importantly, the introduced measures on TSS may need to be taken with caution for large loci (>100 kb). For example, the *Ebf1* and *Nos1ap* TSS particles are, in fact, constantly ‘caged’ within their own structural domain independently on their transcriptional state.

### TADdyn simulations characterize the time-dependent variations in domain borders

**Fig. 4:**
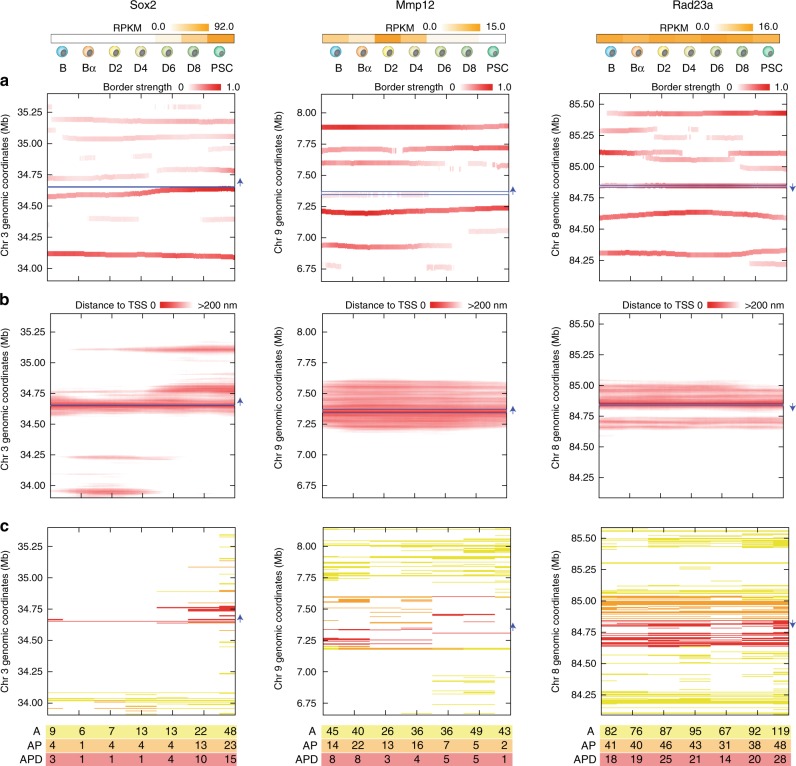
Gene activity correlates with domain borders and enhancer proximity. Top of the figure indicates expression level (RPKM) for each of the selected loci at each reprogramming stage^35^. **a** Time dependent position of the domain borders as defined by the insulation score analysis on the contact maps derived from the models. The position of the loci in the graph is indicated by a blue horizontal line and its transcriptional orientation is indicated by a blue arrow. **b** Heat maps of the distances between the TSS particle and all the other model particles as a function of time. **c** Particles classification into active (A, yellow), active and proximal to the TSS (AP, orange), and active, proximal and within the domain (APD, red). The bottom table shows the number of particles in each category for each reprogramming stage.

To further characterize the possible topological transitions between gene expression states, we calculated dynamic changes in TAD insulation score and border strength^42^ using the contact maps of the models along the TADdyn trajectories (“Methods”). We found that, although the number of borders was overall constant during the simulation, their position often changed. In a striking example involving the *Sox2* locus, during the D4-D6 transition there was a clear shift of a border at position chr3:34.60 Mb, moving further downstream and converging near the TSS (Fig. 4a). A second border at position chr3:34.74 Mb was displaced about 20 kb downstream, thereby removing the topological insulation of a super-enhancer region at chr3:34.70 Mb and the TSS (Fig. 4b). These border changes resulted in the formation of a domain of about 120 kb, which included exclusively the *Sox2* gene and its super-enhancer region. The fact that the border strength remained high after gene activation at D6 is in agreement with our previously described findings that the *Sox2* TSS and its super-enhancer became isolated in a sub-TAD when the gene was transcribed in PSCs (Fig. 4a). Consistent with this observation, the *Mmp12* TSS was partially included in a weak domain border at chr9:7.25 Mb during transcription. This weak border disappeared during the gene’s transition from an active to an inactive state (during the D4-D6 stages), so that the *Mmp12* TSS became part of a larger domain at the time of gene silencing. Finally, and with a similar trend, the TSS of the invariantly active gene *Rad23a* remained part of a domain border, and insulated in an unvaried domain between chr8:84.58 and 82.86 Mb for the entire simulation. This trend, which may not be general, was not observed for other loci where the proximity to domains borders did not correlated with the transcriptional state. Overall, TADdyn simulations indicate that border formation may not be the only structural property correlating with transcriptional activity.

### The formation of 3D hubs of active chromatin is a common motif triggering transcriptional activation

To characterize the elements within the borders of TADs containing transcriptionally active loci, we first identified in the models what we call active particles in any time-point of the reprogramming process, which had overlapping signals (at least 250 base-pairs, bp) of ATAC-seq and H3K4me2 ChIP-seq^35^ (“Methods”, Fig. 4b and Supplementary Figs. 2–22l). Upon activation, the *Sox2* gene was positioned spatially close (<200 nm) to a set of particles containing its super-enhancer (SE) about 120 kb downstream of the TSS. Notably, and in line with our previous observations^35^, the TSS-SE proximity started at D4, that is before the *Sox2* transcriptional activity could be detected (D6). This spatial proximity was maintained for the rest of the simulation while the *Sox2* gene was transcribed. Consistently with its constantly active state, the TSS 3D distance profiles of *Rad23a* remained overall constant during reprogramming (Fig. 4b) with marginal changes during the simulation, while the *Mmp12* late inactivation was not accompanied by a change in its proximity with other genomic regions.

To further assess to what extend active particles (Supplementary Data 2) became proximal to the TSS of the locus, we counted for each reprogramming stage how many of the active (A) particles became available to the TSS particle, either by spatial proximity only (active-proximal particles, AP) or also by sharing the same local domain (active-proximal-domain, APD) (“Methods” and Fig. 4c). Interestingly, this analysis resulted in a robust trend for all analyzed loci, showing higher numbers of AP and APD particles when the locus was transcriptionally active. Notably, for *Sox2* the A, AP and APD particles were increasing together with the locus transcription activity, but the regions containing the annotated super-enhancer were only classified as AP or APD at the D6 stage, that is after the structural changes observed during the simulation at D4 stage. For the *Mmp12* simulation the number of A particles did not decrease in the inactive stage, yet the numbers of AP or APD decreased consistently with the gene activity. The *Rad23a* locus was instead characterized by a quite constant and high number of A, AP and APD particles consistently with is stable transcription activity during the entire reprogramming process. Altogether, we observed a consistent 3D co-localization of active and TSS chromatin particles upon activation of the gene, reminiscent of the recently proposed formation of enhancer hubs or condensates^37,43,44^.

The only locus escaping this general trend was *Rnu7* that despite being silent was proximal to many active (A) particles (Supplementary Fig. 17n). However, the TSS of this locus, which was selected using unsupervised analysis (Methods), was surrounded within <5 kb by several genes (*Ptpn6*, *Gm20531*, *Grcc10*, *Gm45234*, and *Atn1*) that were expressed at different levels during the reprogramming process. This may suggest a cross-talk between genes and active particles when they are in close sequential proximity (Supplementary Data 1).

**Fig. 5:**
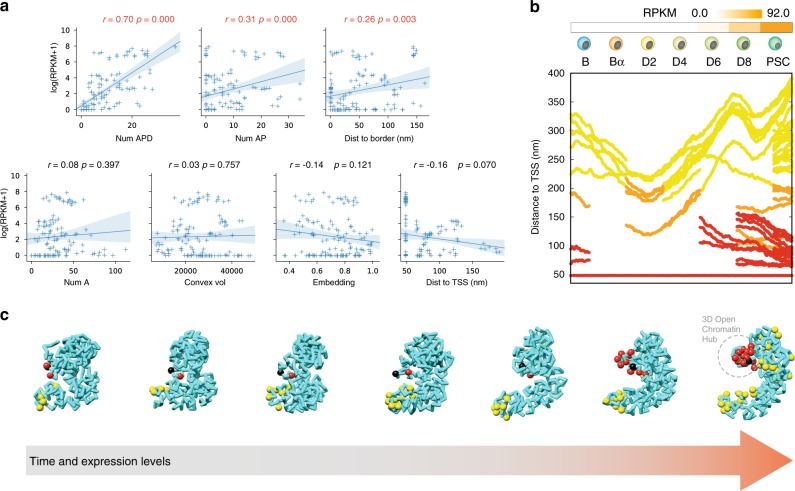
The formation of 3D hubs of active chromatin triggers dynamic gene activation. **a** Correlations between structural features of the models and expression of the resident genes. Each of the seven panels show the scatter-plot, the linear regression model fit line, and the 95% confidence interval between expression (log(RPKM + 1)) with respect number of APD particles, number of AP particles, TSS distance to the closer border, number of A particles, explored (convex-hull) volume, structural embedding and TSS-TTS distance, respectively. The linear regression r coefficient and p-values, that are shown on top of each scatterplot, have been computed using Python (RegPlot function of the seaborn package). **b** Dynamic changes of the distance to the *Sox2* locus TSS for each APD (red), AP (orange) and A (yellow) particles during reprogramming simulation. **c** Snapshots of the dynamic simulation of the *Sox2* locus simulation with the chromatin represented as a cyan ribbon with the *Sox2* locus TSS as a black sphere and all APD, AP and A particles as red, orange, and yellow spheres, respectively.

Overall, TADdyn simulations clearly indicate a correlation between the formation of an active 3D hub that correlates with the expression of the embedded genes (Fig. 5). Of the seven structural measures (that is, the 3D embedding, the explored space, the genomic distance to the closest domain boundary, the distance to the transcription termination site (TTS), and the numbers of A, AP, and APD particles) only the number of APD and AP particles positively correlated (*r* = 0.71, *p*-value = 0.0) with the transcriptional state (RPKM value) of a given locus. However, there are additional locus specific correlations, which may indicate that other structural factors could affect the regulation of gene expression.

## Discussion

Here we have introduced a computational tool, TADdyn, aimed at studying time-dependent dynamics of chromatin domains during natural and induced cell processes by simulating smooth 3D transitions of chromosome structure. TADdyn is unique in its main features being a data-driven method to simulate structural changes of chromatin over time. Additionally, TADdyn presents several additions with respect to other existing restraint-based approaches. First, chromatin is represented as a fiber of continuous spherical particles mapping each of the input Hi–C matrix bins and embedding the main physical properties of the chromatin fiber, such as chain connectivity, excluded volume, and (optionally) bending rigidity^21^. This chromatin representation, that allow to effectively integrate restraint- and polymer physics-based modeling, implies that distinct regions of the model chain cannot cross each other and, as such, may fail when conflicting restraints from Hi–C are imposed to the same particles, which happened only in 1 of the 2100 simulations performed. Altogether, it demonstrates that the structural rearrangements studied here are possible and satisfy both the physical properties of chromatin and the experimentally derived restraints. Second, TADdyn takes as input an entire set of time-series Hi–C interaction maps and converts the interactions propensities between matrix bins into dynamical spatial restraints between pairs of particles. The latter is an approach allowing to study, on a physically reliable chromatin model, the dynamic transitions of chromatin that provides biological insights not directly accessible by the analysis of Hi–C data alone nor by using other static modelling strategies. Third, TADdyn allows to obtain robust results upon changes of the input datasets, which could be used to predict the effect of genomic perturbations. For example, here we found that in two alternative simulations of the *Sox2* locus region (ΔD2 and ΔD6) that missed one reprogramming step resulted in only marginally affected trajectories. This result suggests that the reprogramming dynamics are dominated by smooth, gradual and physically feasible spatial chromatin rearrangements.

TADdyn’s simulations of 21 loci across the mouse genome have revealed structural features that correlate with the expression levels of the studied genes. In particular, we found that the TSS of the *Sox2* and *Mmp12* loci gets embedded inside a structural cage making it less accessible to external particles and more spatially constrained against random thermal motion (Figs. 3 and 4). These findings are consistent with previous super-resolution imaging studies suggesting that the mobility of the promoter is constrained upon activation^45,46^. However, this effect is not observed for other simulations, which is also consistent with the variety of dynamical behaviors described in the literature^47^.

Interestingly, within the cage the TSS establishes an increasing number of interactions with enhancer-like chromatin regions compared to the inactive phases (Fig. 4). Notably, within the caged environment, active particles (that is, with ATAC-seq and H3K4me2 signals) often include annotated enhancers as well as newly predicted putative enhancers. Such predicted enhancers can act at long-range, as exemplified by the distal active particles interacting with the *Sox2* promoter. Upon transcription activation, the dynamic formation of local topological domains (“cages”) inferred here from high-resolution time-resolved Hi–C data, could be reminiscent of structural or topological motifs (such as rosettes or cliques) suggested in previous studies using polymer-physics-based models^48^ as well as transcription factories or phase-separated condensates^43,44,49,50^. We speculate that these cages might trigger and maintain the activation of some promoters by facilitating their local association with a series of proximal enhancers or open chromatin sites. At a larger scale, such single-gene cages might eventually coalescence into larger domains to form large multi-gene condensates^43,51^.

Additionally, to the caging of active chromatin observed in our simulations, TADdyn also revealed that different structural mechanisms may correlated with the expression of specific loci. Some loci are, in fact, characterized by the absence of correlation with either the caging effect or the confined dynamics of the TSS or both phenomena (Fig. 5). As previously suggested^47^, our findings indicate that confinement of loci does not always correlated with increased expression and that other structural features could have a role in gene regulation. It is thus important to integrate our simulations with additional experimental observations beyond Hi–C experiments. The models, however, suggest a common mechanism for all the 21 loci upon activation that promoter-enhancer communication^52^ occurs via direct interactions between distant enhancers and its cognate target gene as previously observed in many studies^33,46,49,50,53–62^. Importantly, our models also suggest that the transcriptional activation of a gene could be regulated by a series of spatially proximal enhancers (i.e. 3D enhancer hubs) and that such regulation could be independent of specific pairwise interactions. These results are compatible with recent imaging approaches challenging the need of direct and continuous promoter-enhancer interaction to promote transcription^63–66^. However, as the time difference between consecutive reprogramming samples used to obtain Hi–C interaction maps was in the order of tens of hours we cannot rule out that at smaller scales cell sub-populations diverge from the general mechanism here proposed. Therefore, this limitation could have precluded detection of other possible dynamic pathways of genome conformation, satisfying only a subset of the input interaction matrices. To address this, TADdyn will have to be implemented in the future using data obtained from finer time-resolved Hi–C and/or imaging-based approaches.

## Methods

### Collection of experimental data

Structural data were obtained from in situ Hi–C chromatin interaction experiments previously generated by us^35^. Specifically, the datasets were downloaded from the GEO database (accession number GSE96611) for the cells stages from B to PSC cells of the reprogramming process in mouse. The dataset included seven Hi–C experiments: B cells (B), Bα cells (after 18 h, Bα), day2 (after 48 h, D2), day4 (after 48 h, D4), day6 (after 48 h, D6), day8 (after 48 h, D8), and Pluripotent Stem Cells (after about 48 h, PSC). The reads were mapped onto the *Mus musculus* reference genome (mm10, Dec 2011 GRCm38) using the fragment-based strategy and filtered for invalid reads, such as self-circles, dangling ends, errors, extra dangling ends, duplicated and random breaks^35^. Using the filtered fragments, the genome-wide raw interaction maps were binned at 5 kilo-base (kb) and normalized using the Vanilla algorithm^67,68^ as implemented in TADbit^38^. Genomic regions were selected around 21 loci of interest (Supplementary Data 1) each characterized by a specific gene expression profile during the cell reprogramming process (Supplementary Figs. 2–22a).

The selection of the loci of interest was aimed to explore scenarios with diverse gene expression dynamics and with the maximum propensity to be accurately modelled along the reprogramming process. This included 11 loci of general interest including: two mostly active loci (*Rad23a* and *Rad23b*), the early activated *Tet2*, the late activated *Sox2* and *Nanog* reprogramming genes, the transiently activated *Nos1ap* gene, the transiently silenced *Lmo7*, and the gradually silenced *Mmp3*, *Mmp12, C/EBPα* and *Ebf1* genes (Supplementary Data 1, Supplementary Figs. 2–22a and Supplementary Movies 1–21). Additionally, we simulated 10 control loci including 5 continuously silent genes (RPKM = 0) (*Rnu7*, *Neurod6*, *Rergl*, *Olfr33*, and *Olfr1002*) and 5 continuously active genes (RPKM maximum) (*Hsp90ab1*, *Ppia*, *Rpl41*, *Rps14*, and *Rps26*). The control loci were selected in an unsupervised way by looking among the completely silent (45 loci) and the mostly expressed genes (top 45 loci) (Supplementary Fig. 1). The Hi–C matrices containing the selected loci were next assessed for their potential to result in accurate 3D models using the Matrix Modelling Potential (MMP)^41^. All Hi–C interaction matrices of these loci had MMP scores higher than 0.78, and contained very few low-coverage bins (<0.05% of the bins), indicating that the restrained-based approach at 5 kb would provide accurate 3D models for all the regions.

The majority of the selected genes span less than 100 kb and where simulated in the center of 1.5 mega-bases (Mb) chromatin regions. For the *Nanog* locus, the modelled region contained only 1 Mb around the locus after filtering low coverage bins (that is, those with more than 75% of cells with zero counts), and for the *Mmp12* (chr9:7,344,381–7,369,499 bp) as well as *Mmp3* (chr9:7,445,822-7,455,975 bp) neighbor loci, a single region of 1.5 Mb was considered (chr9:6,650,000–8,150,000 bp). Three loci (*Ebf1*, *Lmo7*, and *Nos1ap*) were longer than 100 kb and were simulated in 3.0 Mb regions centered on the gene promoter (Supplementary Figs. 3, 5, and 10b).

### Representation of the chromatin region using bead-spring polymer model

TADdyn represents chromatin as a bead-spring polymer describing the effective physical properties of the underlying fiber^18,21^. Specifically, each Hi–C-matrix bin of 5 kb was represented as a spherical particle of diameter 50 nm using a compaction ratio of 0.01 bp/nm^69,70^. Additionally, two non-harmonic potentials were introduced taking into account the excluded volume interaction (purely repulsive Lennard-Jones) and the chain connectivity (Finitely Extensible Nonlinear Elastic, FENE)^18^. In the present application, the bending rigidity potential (although it is available in TADdyn) is not used for consistency with the initial models generated using the TADbit software at the B stage (see below). The chromatin chain was simulated inside a cubic box of size 50 μm (much larger than the size of the models), which was centered at the origin of the Cartesian axis O = (0.0, 0.0, 0.0). To avoid any border effect, the center of mass of the chromatin chain was tethered to the origin O using a Harmonic (*K*_t_ = 50., *d*_eq_ = 0.0).

To account for the physical properties of chromatin, TADdyn requires as initial model conformations already connected polymer chains with no overlapping particles. This initial condition can be obtained in TADdyn in three ways: (i) a generic random self-avoiding walk, (ii) a rod-like arrangement made of stacked rosettes^18^, or (iii) a previously generated data-driven model. For the 100 time-series simulations performed here, the initial conformations at B cell stage (the first Hi–C time point) were the 100 optimal models built using TADbit^38,71^. Briefly, TADbit generates 3D models using a restraint-based modeling approach, where the experimental frequencies of interaction are transformed into a set of spatial restraints (Fig. 1a, b). The size of each particle in the models is defined by the relationship of 0.01 bp/nm assuming the canonical 30 nm fibre^69,70^. Using a grid search approach, TADbit identifies empirically three optimal parameters to be used for modeling: (1) maximal distance between two non-interacting particles (maxdist); (2) a lower-bound cutoff to define particles that do not frequently interact (lowfreq); and (3) an upper-bound cutoff to define particles that frequently interact (upfreq). Once the three optimal parameters are defined, TADbit sets the type of restraints between each pair of particles considering an inverse relationship between the frequencies of interactions of the contact map and the corresponding spatial distances. Two consecutive particles are next spatially restrained by a harmonic oscillator with an equilibrium distance that corresponds to the sum of their radii. Non-consecutive particles with contact frequencies above the upper-bound cutoff are restrained by a harmonic oscillator at an equilibrium distance, while those below the lower-bound cutoff are maintained further than an equilibrium distance by a lower-bound harmonic oscillator. To identify 3D models that best satisfy all the imposed restraints, the optimization procedure is then performed using a Monte Carlo simulated annealing sampling.

### Converting the experimental data into TADdyn restraints

All possible combinations of the parameters (lowfreq, upfreq, maxdist, dcutoff)^71^ were explored in the intervals lowfreq = (−3.0, −2.0, −1.0, 0.0), upfreq = (0.0, 1.0, 2.0, 3.0), maxdist = (150, 200, 250, 300, 350, 400) nm, and dcutoff = (150, 175, 200, 225, 250) nm. To select the optimal set of parameters, the Spearman correlation coefficient (SCC) of the input Hi–C interaction map in the B cell stage and the models contact map was computed per each value of dcutoff using only the 100 best models (that is, with the 100 with lowest objective function over the 500 generated structures). Next, per each triplet of parameters, the median Spearman correlation values were computed over the 21 studied loci. The largest median correlation coefficient of 0.78 was obtained for lowfreq = −1.0, upfreq = 1.0, maxdist = 300 nm, and dcutoff = 225 nm.

Next, the 100 TADbit generated models in B cells were energy minimized using a short run of the Polak-Ribiere version of the conjugate gradient algorithm^72^ to favor smooth adaptations of the implementations of the excluded volume and chain connectivity interaction in TADdyn.

The optimal TADbit parameters optimized for the B stage (that is, lowfreq of −1.0, upfreq of 1.0, and maxdist of 300 nm) were then used to define the set of distance harmonic restraints of the other time points of the series (Supplementary Fig. 23 and 1b). To adapt the harmonic restraint of a given pair of particles (i,j) between consecutive cell stages c_n_ and c_n + 1_, one of the following 3 possible scenarios was applied:

If the pair (i,j) was restrained by the same type of distance restraint (Harmonic or LowerBoundHarmonic) in both c_n_ and c_n+1_, the strength (k) and the equilibrium distance (*d*_eq_) of the harmonic were both changed linearly from the values they had in c_n_ to the values they had in c_n+1_ (Supplementary Fig. 23).
(a) If the distance restraint applied between (i,j) was present at time c_n_, but vanished at time c_n+1_, the strength k was decreased from the value at c_n_ to 0.0, and the equilibrium distance *d*_eq_ was kept constant and equal to the value in c_n_. (b) If the distance restraint was present only at time c_n+1_, the strength k was increased from 0.0 at c_n_ to the value in c_n+1_, and the equilibrium distance *d*_eq_ was kept constant and equal to the value in c_n+1_.
If the pair (i,j) was restrained by different type of distance restraint (Harmonic to LowerBoundHarmonic, or vice versa) in c_n_ and c_n+1_, two distance restraints were defined for (i,j). The restraint which was active at time c_n_ was then switched off as in case 2a, and the one active at time c_n+1_ was switched on as in 2b (Supplementary Fig. 23).

### TADdyn restraint-based dynamics simulations

By applying the previous protocol, the simulation effectively and smoothly modified the underlying restraints during the steered transition from c_n_ to c_n+1_. The dynamics of the system was thus described using the stochastic (Langevin) equation^73^, which was integrated using LAMMPS^74^ (http://lammps.sandia.gov) with values of the particle mass (*m* = 1.0), the friction (*γ* = 0.5 τ_LJ_^−1^), and the integration time step of dt = 0.001 τ_LJ_, where τ_LJ_ is the internal time unit^75^. The time-dependent Harmonic and LowerBoundHarmonic restraints were implemented using the Colvars plug-in for LAMMPS^76^ originally introduced for advanced sampling techniques, and here modified to implement the TADdyn transitional restraints. The transition between consecutive time stages was set to last for 10 τ_LJ_, hence a single run to simulate the complete B to PSC reprogramming process passing through the seven-cell stages lasted for 60 τ_LJ_. In each of the 100 replicates of the reprogramming run, the model conformations were stored every 0.1 τ_LJ_, and used for further data analysis.

To test whether the total number of replicate trajectories (100) was enough to provide a good description of the variability of the ensemble of trajectories, we generated for *Sox2* and *Mmp12* loci 2 additional runs producing respectively 500 and 1,000 replicates. Additionally, to test the predictive power of TADdyn in case of smooth structural rearrangements two additional simulations were performed for the *Sox2* locus by removing the restraints of stages D2 (ΔD2) and D6 (ΔD6), and by extending the corresponding transitions (Bα → D4 and D4 → D8 respectively) from 10 to 20 τ_LJ_ to keep constant the total duration of the runs (60 τ_LJ_).

### Time dependent contact maps and TADdyn models’ assessment

(i) The contact maps were computed at each time step based on the probability within the 100 simulations that pairs of particles are contacting (that is within a distance cut-off of 200 nm) (Fig. 2a and Supplementary Figs. 2–22c). Videos of the contact maps along the reprogramming process are shown in the right panels of Supplementary Movies 1–22. (ii) The resulting contact maps along the simulations were clustered. The Spearman’s rank correlation coefficients (SCC) was computed with each of the time-series Hi–C interaction map. The SCCs were converted into normalized distances from 0 (max SCC) to 1 (min SCC) and used to cluster the contact maps using the Ward hierarchical approach criterion^77^ as implemented in R (Fig. 2b and Supplementary Figs. 2–22d). Analogous analysis was performed on the interaction Hi–C maps for the 2 replicates and the merged Hi–C experiments (Supplementary Figs. 2–22e) (iii) For each simulation, the distance root mean square deviation (dRMSD) between the optimally superimposed models (separated by 1 τ_LJ_) was computed. Next, per each model pair, the median dRMSD over the 100 replicates was computed. Finally, a structural clustering analysis was done on the matrix of median dRMSDs by using the Ward hierarchical approach criterion as implemented in R (Fig. 2b and Supplementary Figs. 2–22f).

### Time dependent measures of the TADdyn structures

(i) The accessibility (A) of each particle in the ensemble of models was calculated using TADbit with parameters nump = 100, radius = 50 nm, and super-radius = 200 nm. The accessibility was, next, converted into embedding (E = 1.0-A) that is a measure of the propensity of a particle to be caged in an internal cavity inside the model structure. The embedding ranges from 0.0 meaning that the particle is located on the interface of the model to 1.0 when the particle is closely surrounded by other particles inside the model (caged) (Fig. 3a and Supplementary Figs. 2–22g). (ii) The explored volume per particle every 5 τ_LJ_ of trajectory was calculated. Specifically, all trajectories were partitioned in time intervals of 5 τ_LJ_. In each time interval, the 50 positions (i.e. one every 0.1 τ_LJ_) occupied by the particle i were considered, and the convex-hull embedding these 50 positions was calculated. In a given time interval, the average (over the 100 replicate runs) convex-hull volume explored by particle i was considered as the typical volume explored by the model particle i during the time interval. The convex-hull values are represented as boxplots (geom_boxplot function of the R package ggplot2) showing: central line, median; box limits, 75th and 25th percentiles; whiskers, 1.5× interquartile range. Outliers are not shown. The statistical comparison between the distributions of the explored volume was performed with a two-sided Wilcoxon test using R. The comparisons that resulted in *p*-values < 0.0001 were deemed to be significantly different. (Fig. 3b and Supplementary Figs. 2–22h). (iii) The spatial distance between selected pairs of particles was computed for the ensemble of simulations as the Euclidian distance in nanometers using TADbit (Fig. 4a and Supplementary Figs. 2–22k).

### Time dependent insulation score analysis of TADdyn models

To study the partitioning of the models and the Hi–C interaction maps into structural domains (reminiscent of TADs^78–80^), the insulation score (I-score) analysis^42^ was performed on the models contact maps using the parameters --is 100000 --ids 50000 --ez --im mean --nt 0.1 --bmoe 3. The called domain borders, whose border strength was deemed significant by the I-score pipeline, were used for further analysis (Fig. 4b and Supplementary Figs. 2–22I, j).

### Active particles analysis

In each cell stage, we classified model particles (5 kb) into one of 3 possible categories (Supplementary Data 2, Fig. 4c and Supplementary Figs. 2–22l): active (A) are particles hosting at least one overlapping 250bp-peak of ATAC-seq and H3K4me2 (ATAC-seq and H3K4me2 data were obtained from our previous work^35^), active-proximal (AP) are Active particles that are close to the TSS particle of the gene either in absolute terms (spatial distance < 200 nm) or in relative terms (spatial distance < half of average distance at the genomic separation) for at least half of the duration of the stage. Active-proximal-domain (APD) are AP particles that are inside the local domain containing the TSS (see insulation score analysis above).

### Gene expression analysis of RNA-seq data

Reads were mapped with STAR^81^ (-outFilterMultimapNmax 1 -outFilterMismatchNmax 999 -outFilterMismatchNoverLmax 0.06 -sjdbOverhang 100 –outFilterType BySJout -alignSJoverhangMin 8 -alignSJDBoverhangMin 1 –alignIntronMin 20 -alignIntronMax 1000000 -alignMatesGapMax 1000000) and the Ensembl mouse genome annotation (GRCm38.78). Gene expression was quantified (RPKM) with STAR (--quantMode GeneCounts).

### Chromatin accessibility analysis of ATAC–seq data

Reads were mapped to the UCSC mouse genome build (mm10) in Bowtie2^82^ with standard settings. Reads mapping to multiple locations in the genome were removed in SAMtools^83^; PCR duplicates were filtered in Picard. Bam files were parsed to HOMER^84^ for downstream analyses. Peaks in ATAC–seq signals were identified with findPeaks (-region -localSize 50000 -size 250 -minDist 500 -fragLength 0, FDR < 0.001).

### ChIPmentation/ChIP–seq data analysis

Reads were mapped and filtered as described for ATAC–seq. H3K4me2-enriched regions were identified with HOMER findpeaks (findPeaks -region -size 1000 -minDist 2500, by using a mock IgG experiment as background signal).

### Reporting summary

Further information on research design is available in the Nature Research Reporting Summary linked to this article.

## Supplementary information

Supplementary Information

Description of Additional Supplementary Files

Supplementary Data 1

Supplementary Data 2

Supplementary Movie 1

Supplementary Movie 2

Supplementary Movie 3

Supplementary Movie 4

Supplementary Movie 5

Supplementary Movie 6

Supplementary Movie 7

Supplementary Movie 8

Supplementary Movie 9

Supplementary Movie 10

Supplementary Movie 11

Supplementary Movie 12

Supplementary Movie 13

Supplementary Movie 14

Supplementary Movie 15

Supplementary Movie 16

Supplementary Movie 17

Supplementary Movie 18

Supplementary Movie 19

Supplementary Movie 20

Supplementary Movie 21

Reporting Summary

## Data Availability

In situ Hi-C, ChIP-seq (H3K4me2 histone mark), ATAC-seq, and RNA-seq datasets were downloaded from Gene Expression Omnibus (GEO) at the accession number GSE96611.

## Code availability

The TADdyn approach is available as part of the 3DGenome Github repository (https://github.com/3DGenomes/TADbit/tree/TADdyn). Custom scripts used to analyze data and generate figures are available at http://sgt.cnag.cat/3dg/datasets/.
