# Supplementary material for: Transcriptional activation during cell reprogramming correlates with the formation of 3D open chromatin hubs
Source: Nat Commun. 2020 May 22;11:2564. doi: 10.1038/s41467-020-16396-1 (PMC7244774; doi:10.1038/s41467-020-16396-1)
Supplement: Supplementary file 1 — Supplementary Information [file 41467_2020_16396_MOESM1_ESM.pdf]

Transcriptional activation during cell reprogramming correlates with the formation of 3D open chromatin hubs.

Di Stefano et al.

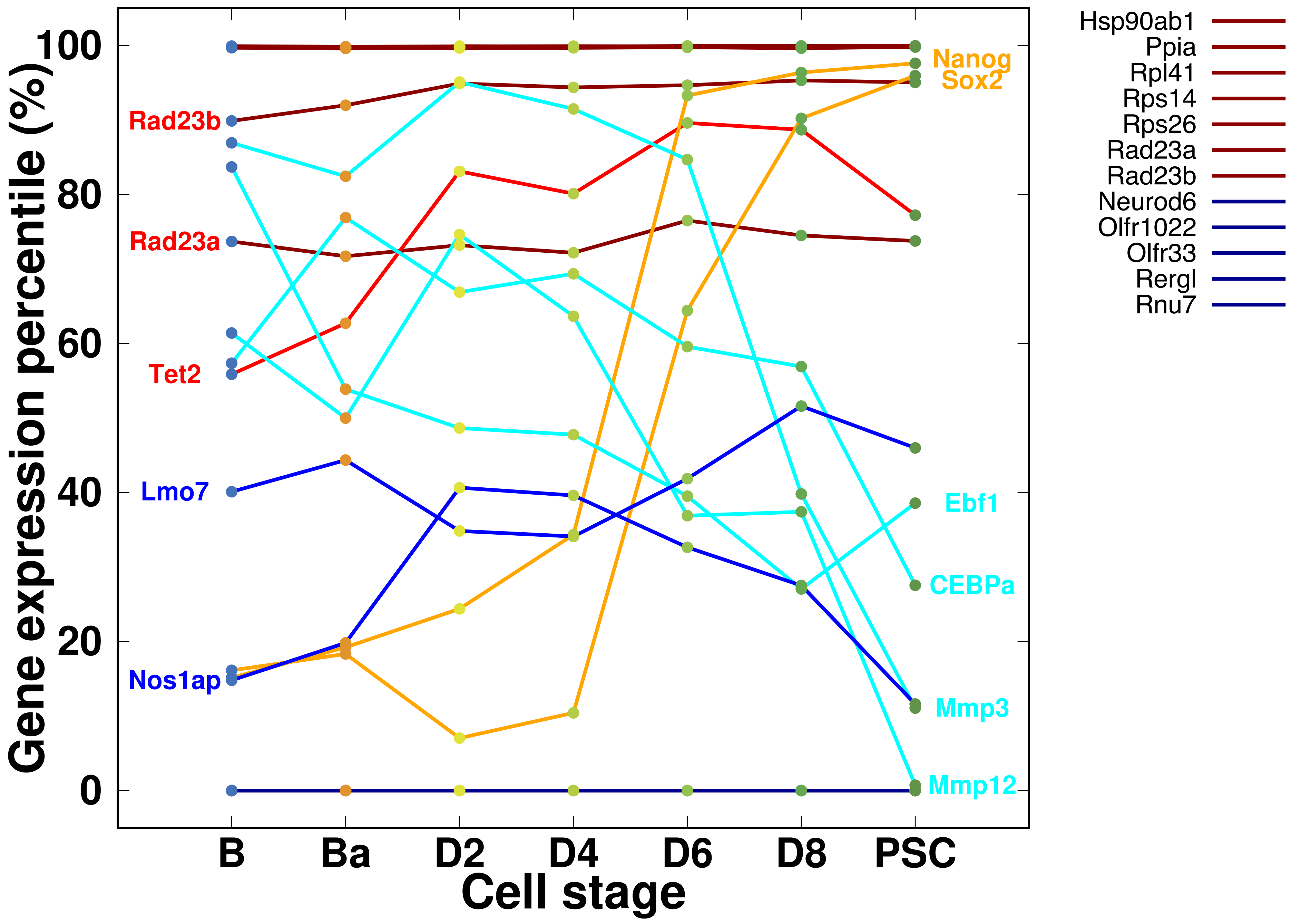

Supplementary Figure 1 . Selection of the simulated loci. The variations in normalized gene expression are shown for the 21 selected loci. 7 are always active (*Hsp90ab1*, *Ppia*, *Rad23a, Rad23b, Rpl41, Rps14* and *Rps26*), 5 are always completely silent (*Neurod6*, *Olfr1022*, *Olfr33*, *Rergl* and *Rnu7*), 1 is early activated (*Tet2*), 2 are late activated (*Sox2* and *Nanog*), 1 is transiently activated (*Nos1ap*), 1 is transiently silenced (*Lmo7*), and 4 are gradually silenced (*Mmp3*, *Mmp12, C/EBP* and *Ebf1*).

CEBPa: chr7:35119292-35121928. Forward

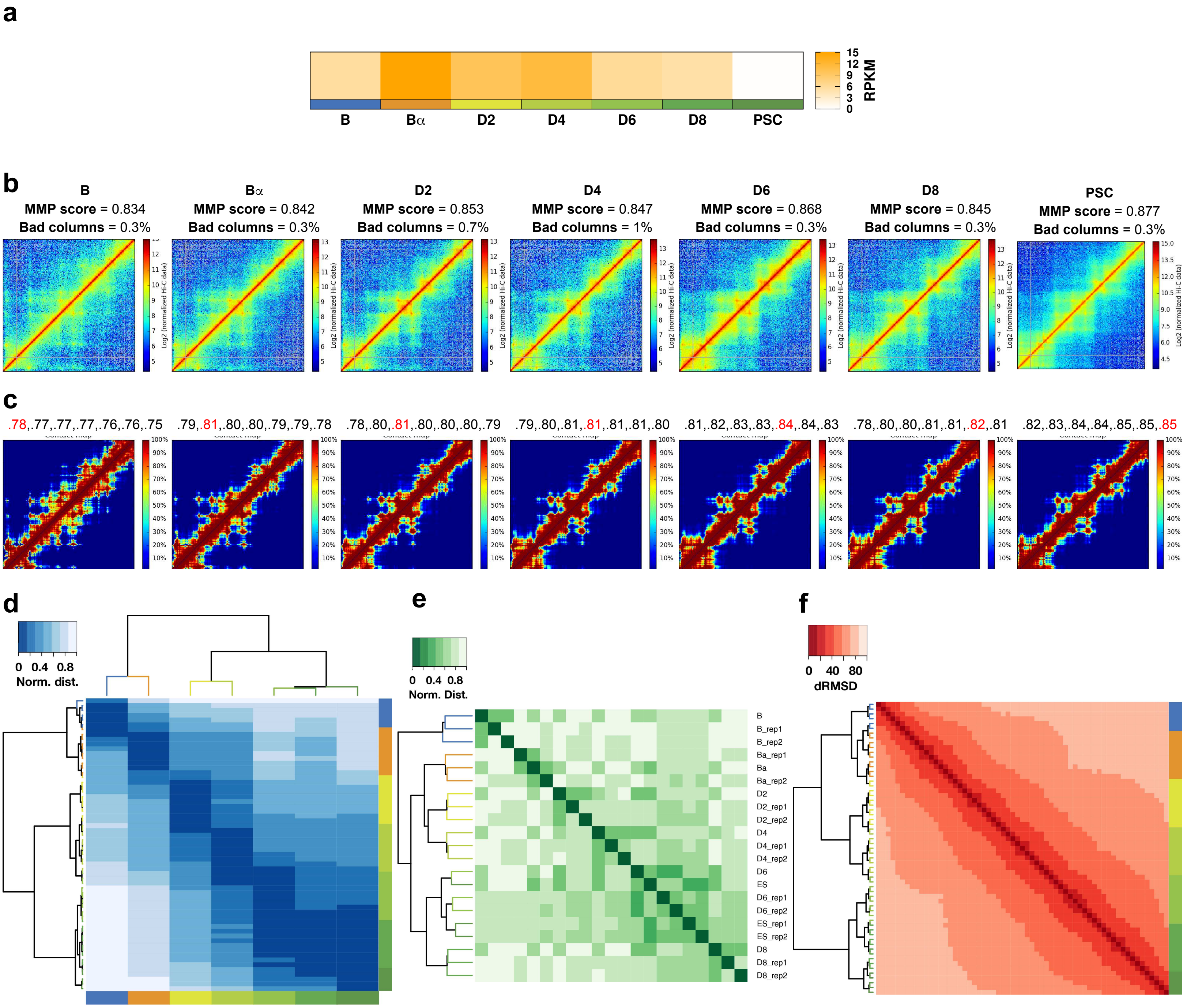

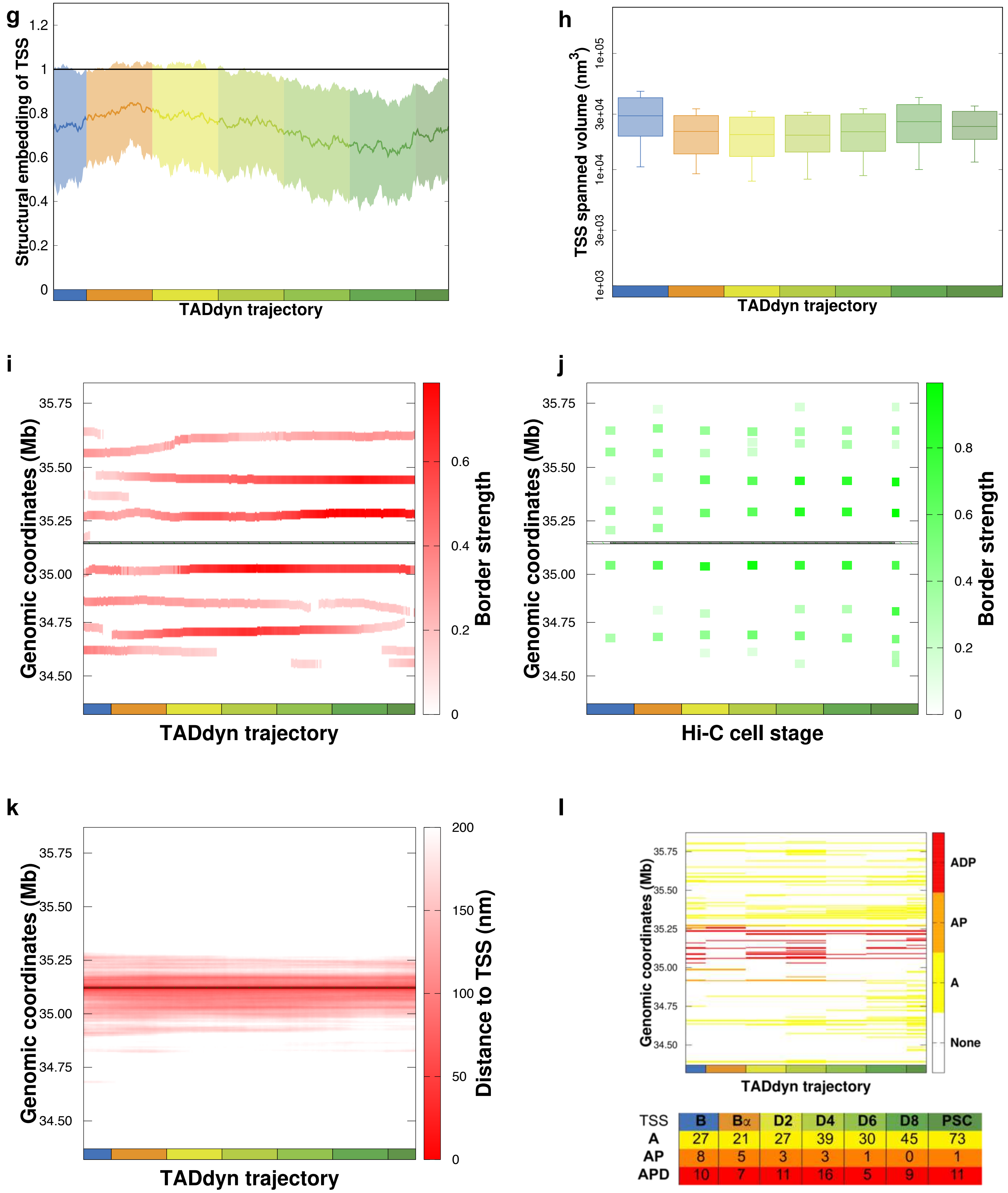

TSS	B	Bα	D2	D4	D6	D8	PSC
A	27	21	27	39	30	45	73
AP	8	5	3	3	1	0	1
APD	10	7	11	16	5	9	11

Supplementary Figure 2. Complete TADdyn analysis of the *CEBPa* simulated locus. The panels present in two pages all the analysis done on the TADdyn models, the Hi-C datasets, the chromatin tracks (ATAC-seq and H3K4me2 peaks), and RNAseq experiments. Specifically, in page 1 we present **(a)** the expression level per cell stage, **(b)** the in-situ Hi-C interaction maps, **(c)** the models' contact maps at dcutoff=200 nm, **(d-f)** the clustering analysis based on models' vs. Hi-C **(d)** and Hi-C vs. Hi-C **(e)** correlations transformed in normalized distances, and models vs. models **(f)** based on structural distance root-mean-squared displacement (dRMSD). On page 2 we show **(g)** the TSS structural embedding along the trajectories where the line represent the average and the colored areas (+/-) the standard deviation, **(h)** the average (over the 100 replicates) of the volume explored by the TSS along the TADdyn trajectories every 5 simulation timesteps at each cell stage represented as boxplots (n=100 data points for B and PSC stages, and n=200 data points for the other cell stages) showing: central line, median; box limits, 75th and 25th percentiles; whiskers, 1.5x interquartile range (outliers not shown), **(i-j)** the domains borders on the models contact maps along the entire trajectory (600 time points) **(i)** and the Hi-C interactions maps at each cell stage (7 time points) **(j)**, **(k)** the heat-map showing the average distance to the TSS of each particle along the TADdyn trajectories, and **(l)** the number of active (A), active-proximal (AP), and active-proximal-domain (APD) particle respect to the TSS. Genome tracks for the annotated genes and regulatory elements (promoters, enhancers, and protein binding sites), and for ATAC-seq, CTCF, and H3K4me2 peaks at each reprogramming stage generated in Stadhouders *et al. Nat. Genet.* **50**, 238-249 (2018) are available here for the *CEBPa* simulated region.

Ebf1: chr11:44618099-45008096. Forward

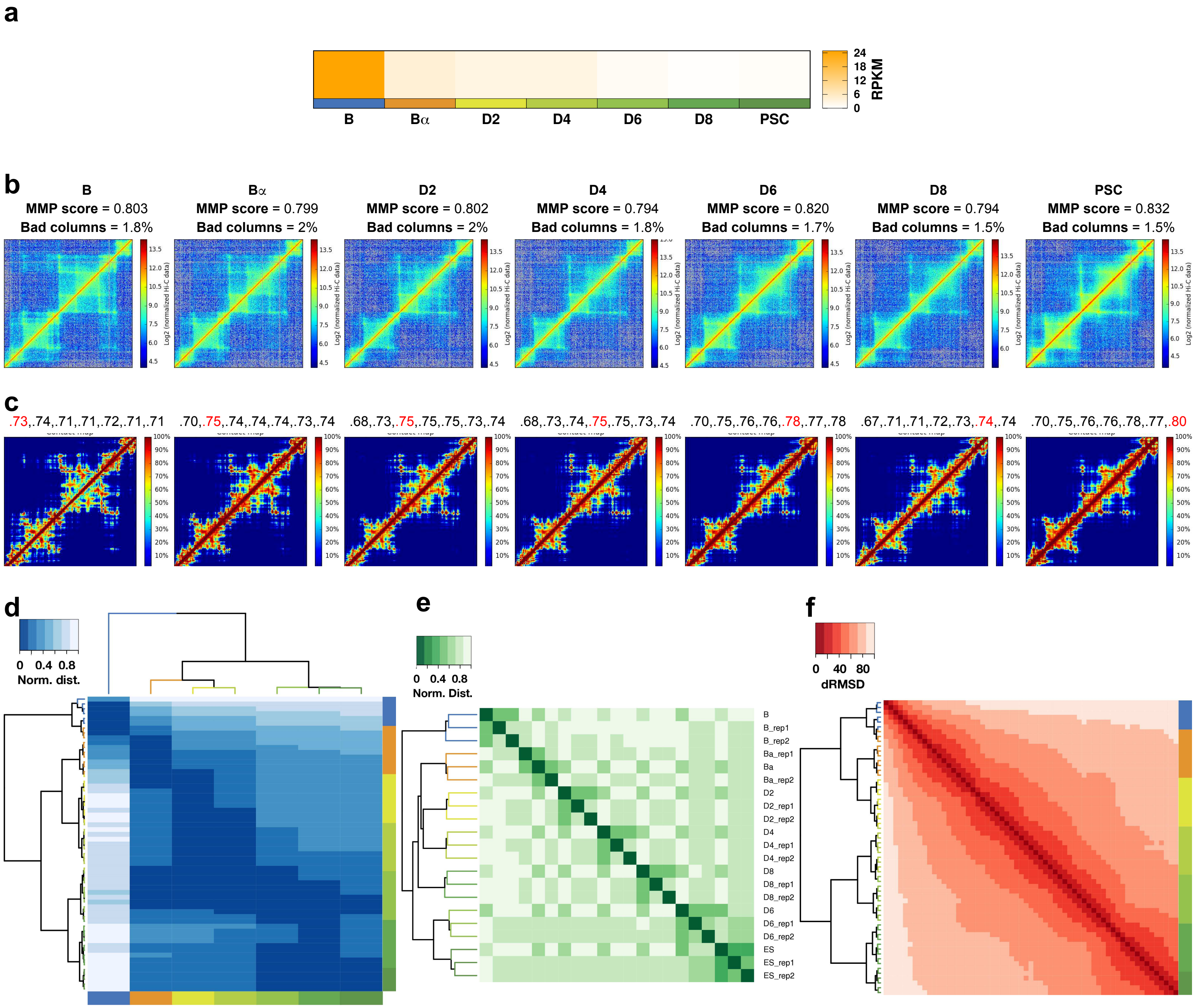

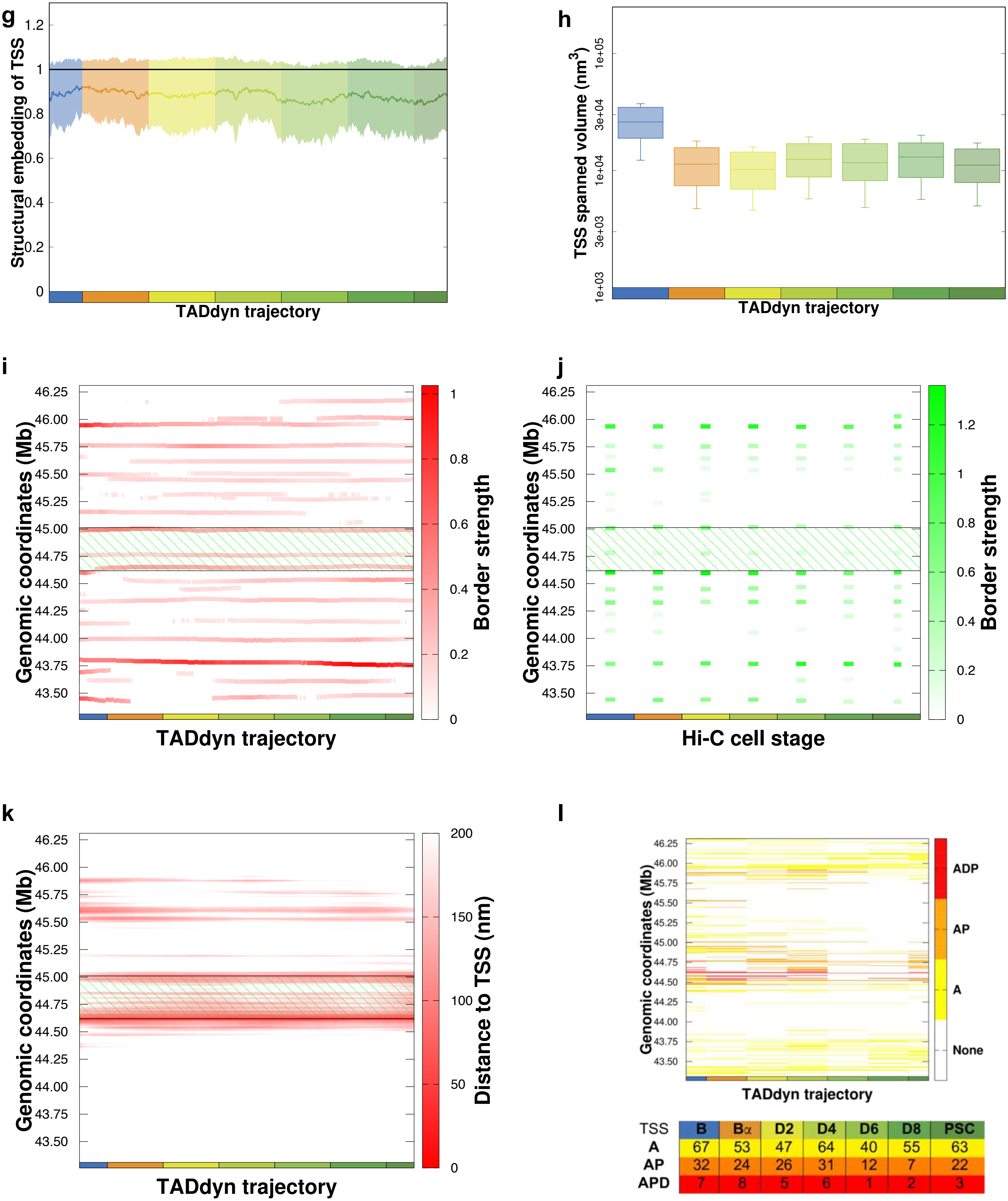

TSS	B	Bα	D2	D4	D6	D8	PSC
A	67	53	47	64	40	55	63
AP	32	24	26	31	12	7	22
APD	7	8	5	6	1	2	3

Supplementary Figure 3. Complete TADdyn analysis of the *Ebf1* simulated locus. The panels present in two pages all the analysis done on the TADdyn models, the Hi-C datasets, the chromatin tracks (ATAC-seq and H3K4me2 peaks), and RNAseq experiments. Specifically, in page 1 we present **(a)** the expression level per cell stage, **(b)** the in-situ Hi-C interaction maps, **(c)** the models' contact maps at dcutoff=200 nm, **(d-f)** the clustering analysis based on models' vs. Hi-C **(d)** and Hi-C vs. Hi-C **(e)** correlations transformed in normalized distances, and models vs. models **(f)** based on structural distance root-mean-squared displacement (dRMSD). On page 2 we show **(g)** the TSS structural embedding along the trajectories where the line represent the average and the colored areas (+/-) the standard deviation, **(h)** the average (over the 100 replicates) of the volume explored by the TSS along the TADdyn trajectories every 5 simulation timesteps at each cell stage represented as boxplots (n=100 data points for B and PSC stages, and n=200 data points for the other cell stages) showing: central line, median; box limits, 75th and 25th percentiles; whiskers, 1.5x interquartile range (outliers not shown), **(i-j)** the domains borders on the models contact maps along the entire trajectory (600 time points) **(i)** and the Hi-C interactions maps at each cell stage (7 time points) **(j)**, **(k)** the heat-map showing the average distance to the TSS of each particle along the TADdyn trajectories, and **(l)** the number of active (A), active-proximal (AP), and active-proximal-domain (APD) particle respect to the TSS. Genome tracks for the annotated genes and regulatory elements (promoters, enhancers, and protein binding sites), and for ATAC-seq, CTCF, and H3K4me2 peaks at each reprogramming stage generated in Stadhouders *et al. Nat. Genet.* **50**, 238-249 (2018) are available here for the *Ebf1* simulated region.

Hsp90ab1: chr17:45567777-45573261. Reverse

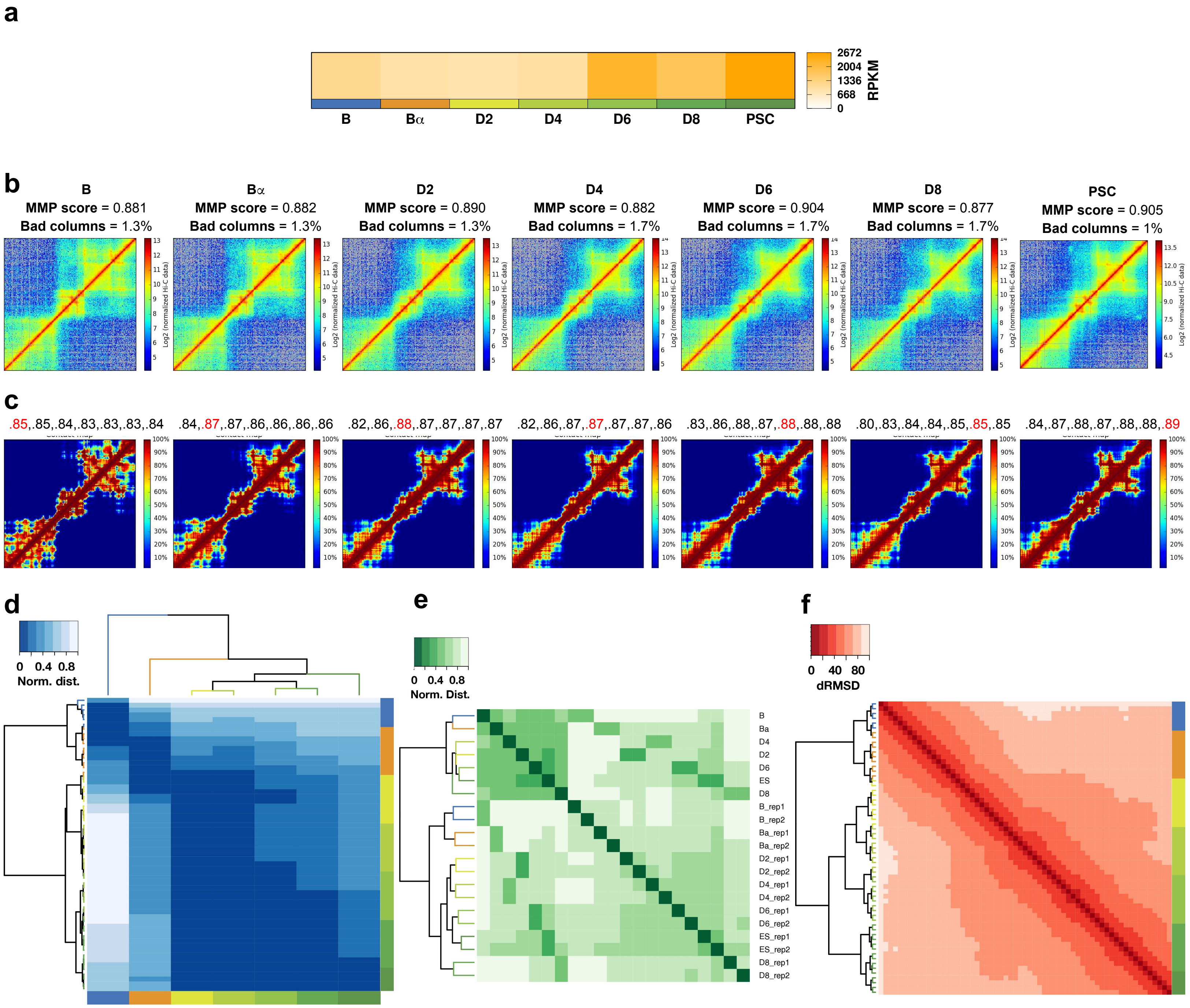

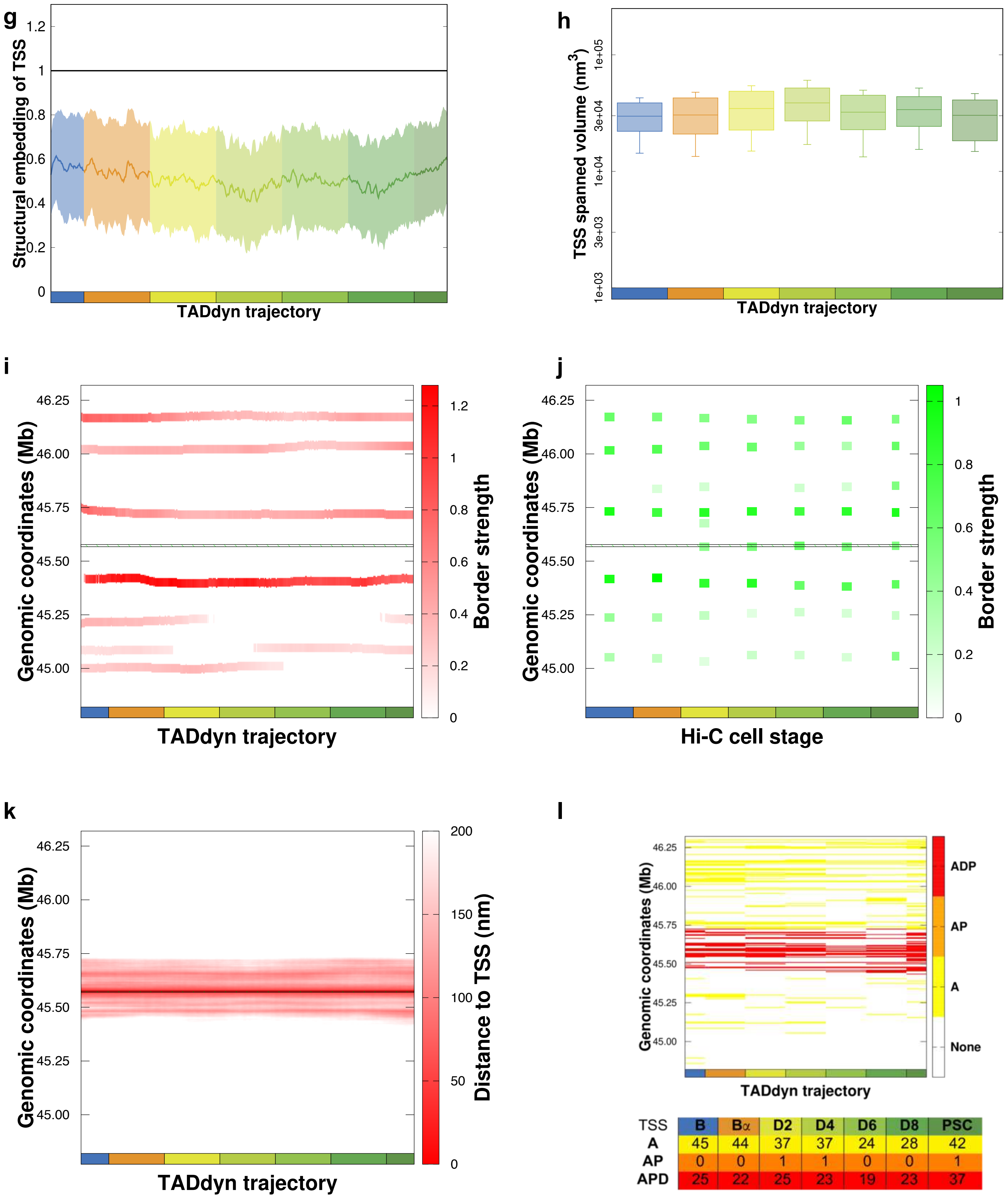

TSS	B	Bα	D2	D4	D6	D8	PSC
A	45	44	37	37	24	28	42
AP	0	0	1	1	0	0	1
APD	25	22	25	23	19	23	37

Supplementary Figure 4. Complete TADdyn analysis of the *Hsp90ab1* simulated locus. The panels present in two pages all the analysis done on the TADdyn models, the Hi-C datasets, the chromatin tracks (ATAC-seq and H3K4me2 peaks), and RNAseq experiments. Specifically, in page 1 we present **(a)** the expression level per cell stage, **(b)** the in-situ Hi-C interaction maps, **(c)** the models' contact maps at dcutoff=200 nm, **(d-f)** the clustering analysis based on models' vs. Hi-C **(d)** and Hi-C vs. Hi-C **(e)** correlations transformed in normalized distances, and models vs. models **(f)** based on structural distance root-mean-squared displacement (dRMSD). On page 2 we show **(g)** the TSS structural embedding along the trajectories where the line represent the average and the colored areas (+/-) the standard deviation, **(h)** the average (over the 100 replicates) of the volume explored by the TSS along the TADdyn trajectories every 5 simulation timesteps at each cell stage represented as boxplots (n=100 data points for B and PSC stages, and n=200 data points for the other cell stages) showing: central line, median; box limits, 75th and 25th percentiles; whiskers, 1.5x interquartile range (outliers not shown), **(i-j)** the domains borders on the models contact maps along the entire trajectory (600 time points) **(i)** and the Hi-C interactions maps at each cell stage (7 time points) **(j)**, **(k)** the heat-map showing the average distance to the TSS of each particle along the TADdyn trajectories, and **(l)** the number of active (A), active-proximal (AP), and active-proximal-domain (APD) particle respect to the TSS. Genome tracks for the annotated genes and regulatory elements (promoters, enhancers, and protein binding sites), and for ATAC-seq, CTCF, and H3K4me2 peaks at each reprogramming stage generated in Stadhouders *et al. Nat. Genet.* **50**, 238-249 (2018) are available here for the *Hsp90ab1* simulated region.

Lmo7: chr14:101729927-101934693. Forward

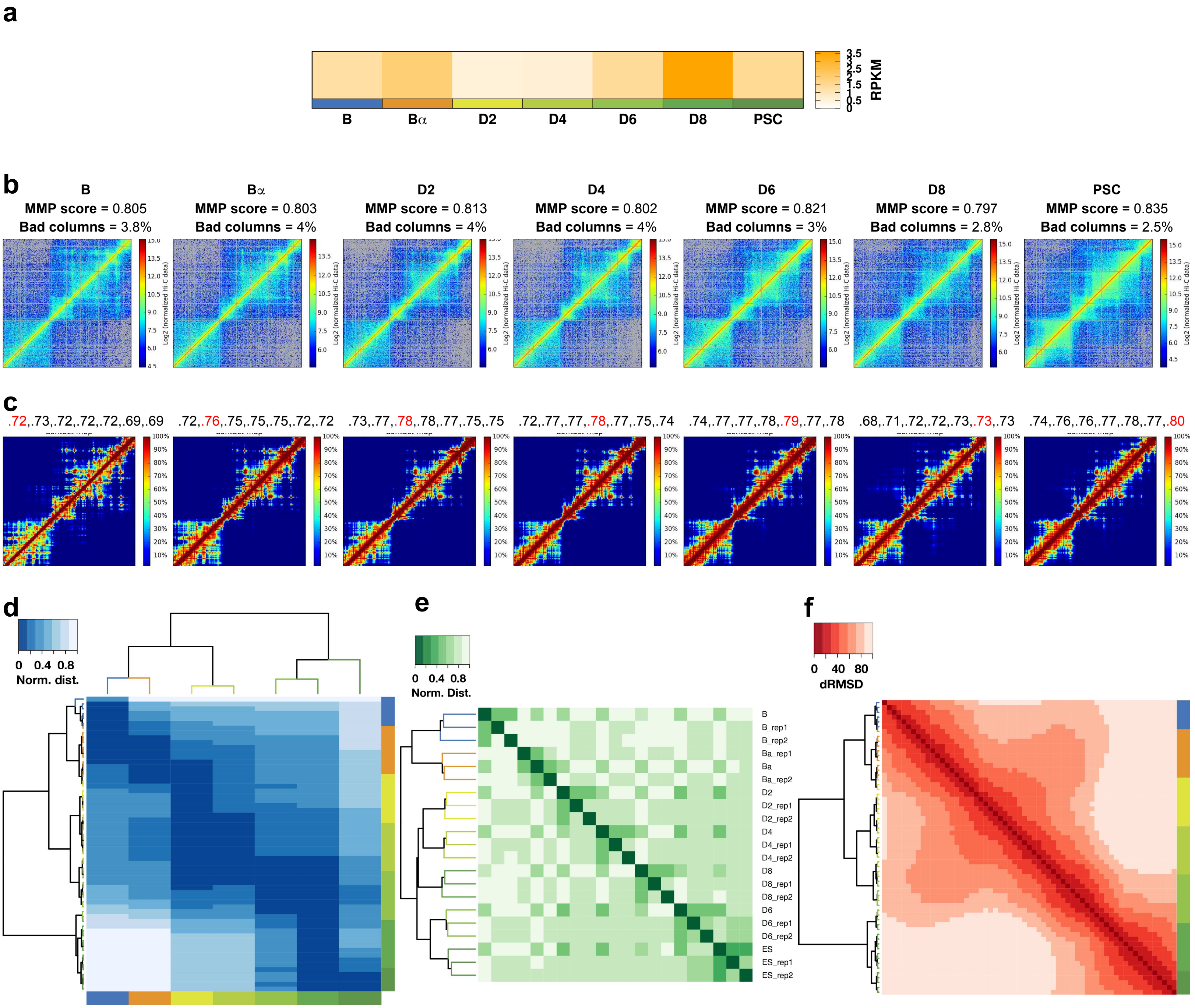

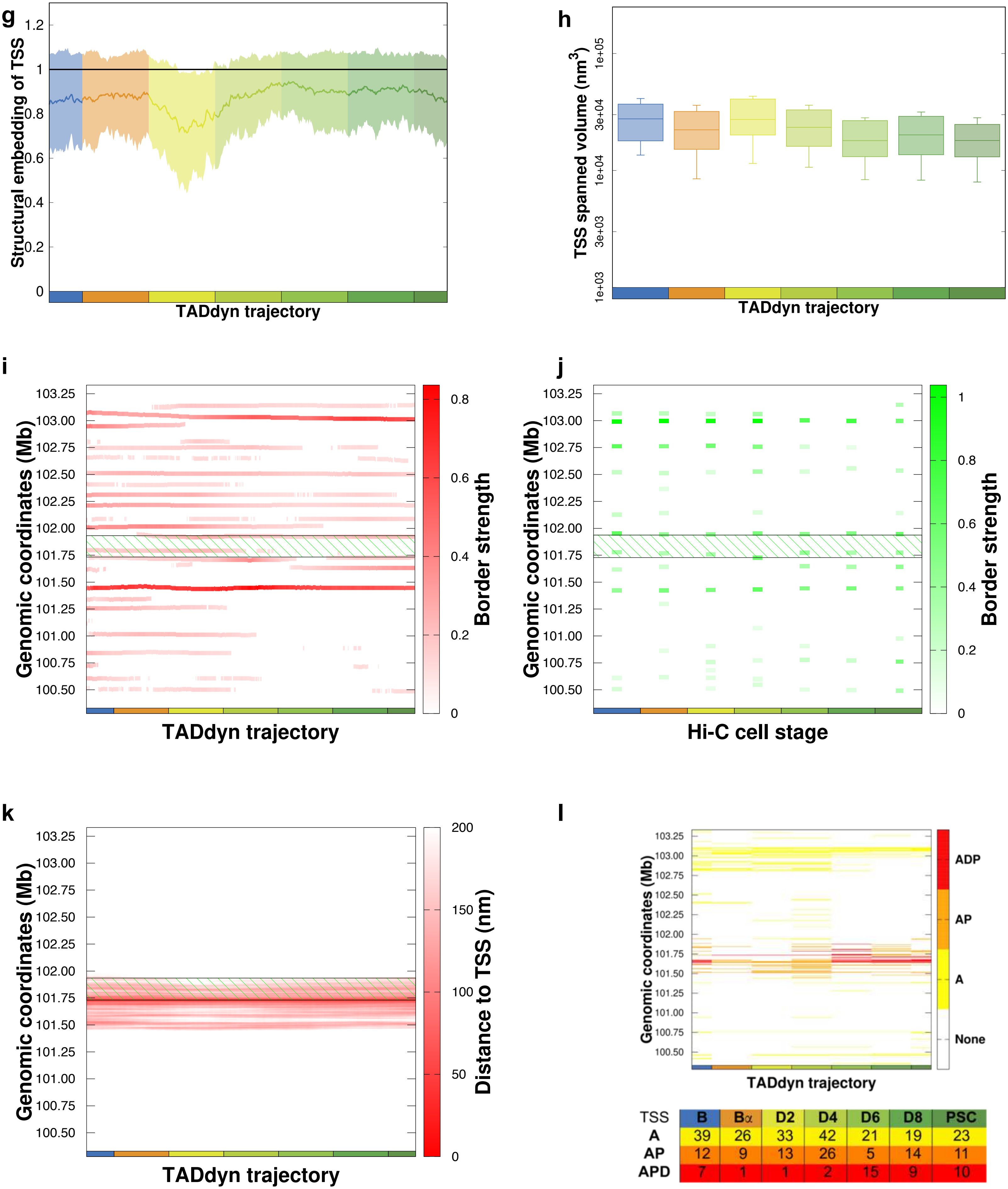

TSS	B	Bα	D2	D4	D6	D8	PSC
A	39	26	33	42	21	19	23
AP	12	9	13	26	5	14	11
APD	7	1	1	2	15	9	10

Supplementary Figure 5. Complete TADdyn analysis of the *Lmo7* simulated locus. The panels present in two pages all the analysis done on the TADdyn models, the Hi-C datasets, the chromatin tracks (ATAC-seq and H3K4me2 peaks), and RNAseq experiments. Specifically, in page 1 we present **(a)** the expression level per cell stage, **(b)** the in-situ Hi-C interaction maps, **(c)** the models' contact maps at dcutoff=200 nm, **(d-f)** the clustering analysis based on models' vs. Hi-C **(d)** and Hi-C vs. Hi-C **(e)** correlations transformed in normalized distances, and models vs. models **(f)** based on structural distance root-mean-squared displacement (dRMSD). On page 2 we show **(g)** the TSS structural embedding along the trajectories where the line represent the average and the colored areas (+/-) the standard deviation, **(h)** the average (over the 100 replicates) of the volume explored by the TSS along the TADdyn trajectories every 5 simulation timesteps at each cell stage represented as boxplots (n=100 data points for B and PSC stages, and n=200 data points for the other cell stages) showing: central line, median; box limits, 75th and 25th percentiles; whiskers, 1.5x interquartile range (outliers not shown), **(i-j)** the domains borders on the models contact maps along the entire trajectory (600 time points) **(i)** and the Hi-C interactions maps at each cell stage (7 time points) **(j)**, **(k)** the heat-map showing the average distance to the TSS of each particle along the TADdyn trajectories, and **(l)** the number of active (A), active-proximal (AP), and active-proximal-domain (APD) particle respect to the TSS. Genome tracks for the annotated genes and regulatory elements (promoters, enhancers, and protein binding sites), and for ATAC-seq, CTCF, and H3K4me2 peaks at each reprogramming stage generated in Stadhouders *et al. Nat. Genet.* **50**, 238-249 (2018) are available here for the *Lmo7* simulated region.

Mmp12: chr9:7347737-7360461. Forward

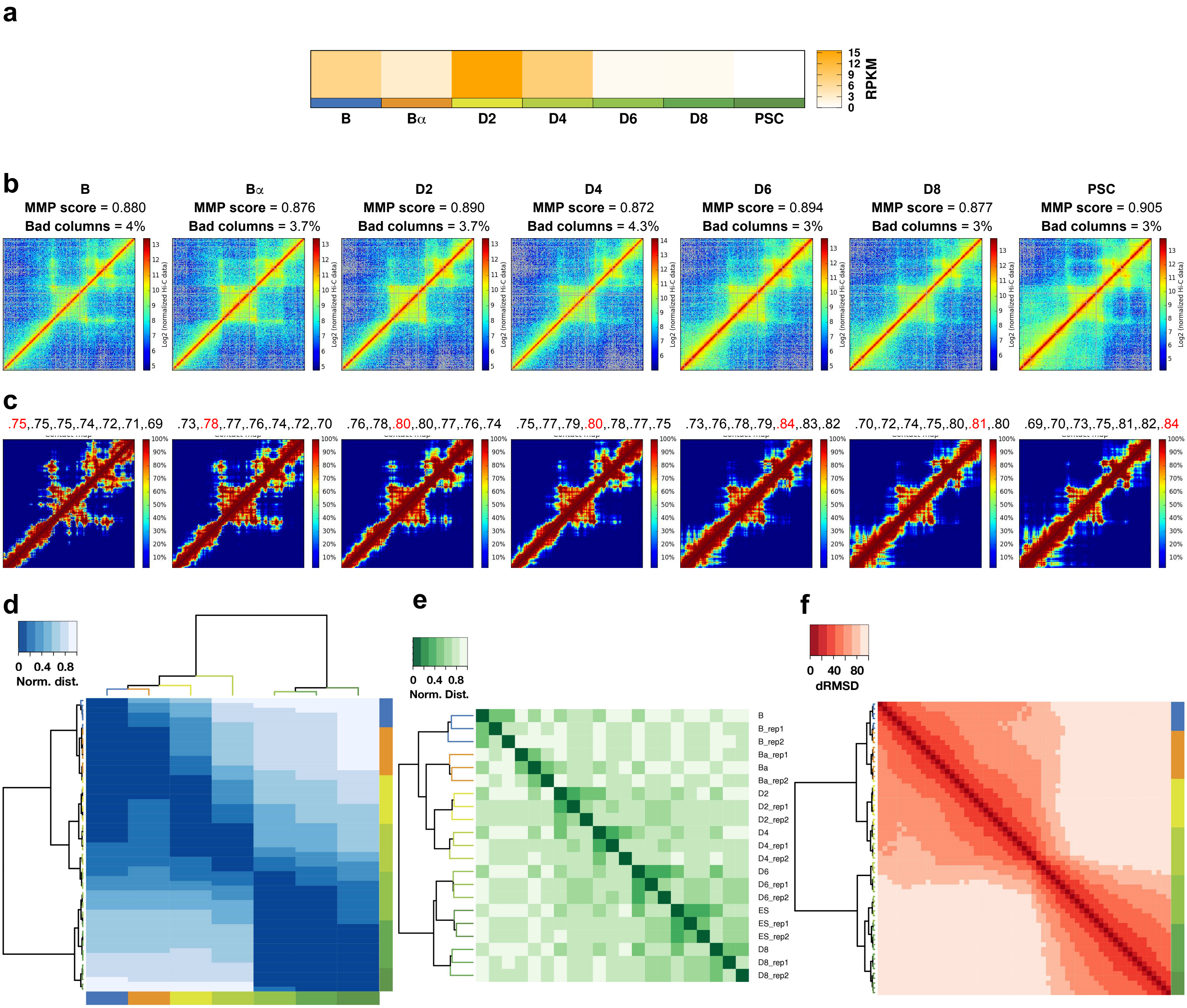

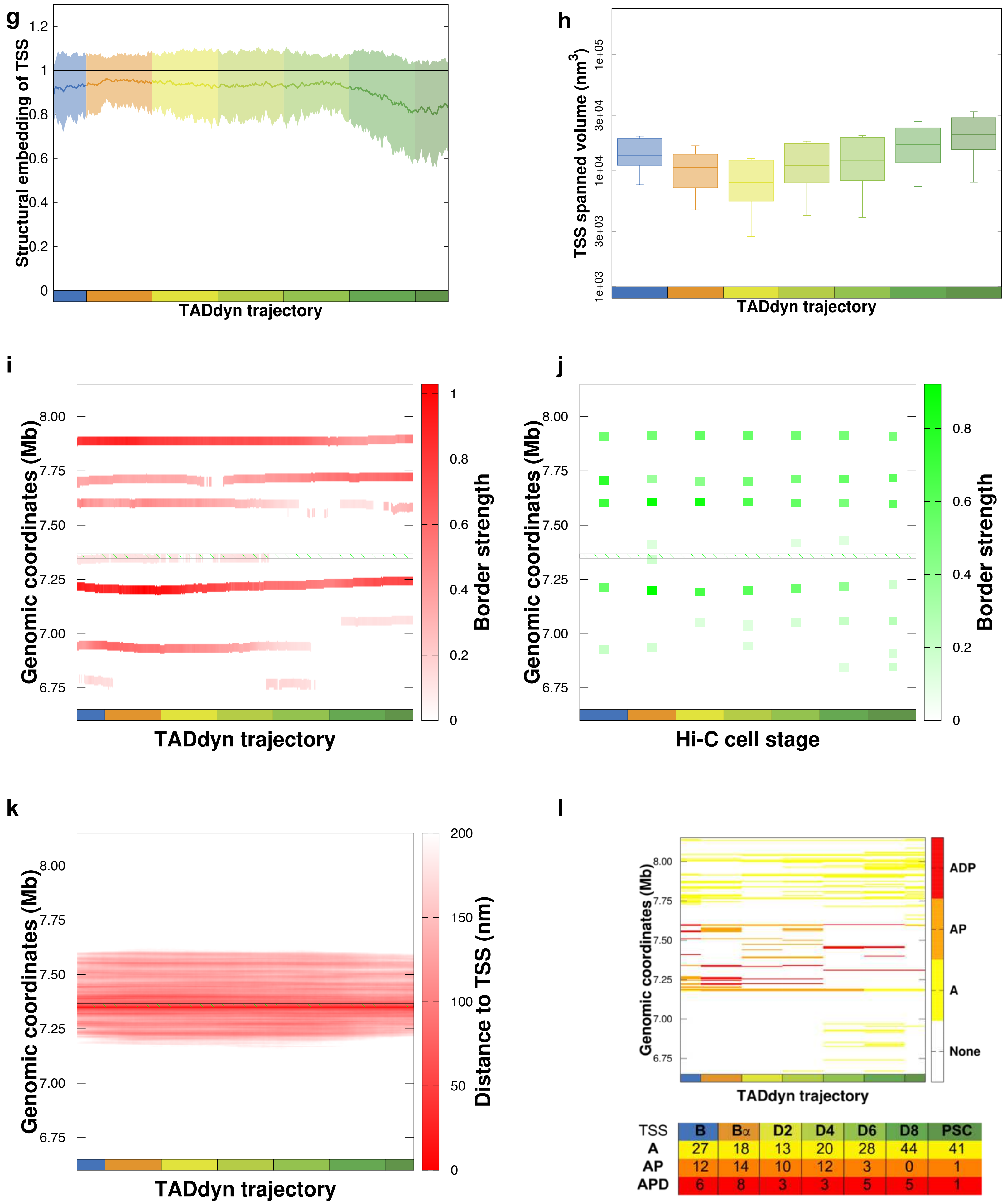

TSS	B	Bα	D2	D4	D6	D8	PSC
A	27	18	13	20	28	44	41
AP	12	14	10	12	3	0	1
APD	6	8	3	3	5	5	1

Supplementary Figure 6. Complete TADdyn analysis of the *Mmp12* simulated locus. The panels present in two pages all the analysis done on the TADdyn models, the Hi-C datasets, the chromatin tracks (ATAC-seq and H3K4me2 peaks), and RNAseq experiments. Specifically, in page 1 we present **(a)** the expression level per cell stage, **(b)** the in-situ Hi-C interaction maps, **(c)** the models' contact maps at dcutoff=200 nm, **(d-f)** the clustering analysis based on models' vs. Hi-C **(d)** and Hi-C vs. Hi-C **(e)** correlations transformed in normalized distances, and models vs. models **(f)** based on structural distance root-mean-squared displacement (dRMSD). On page 2 we show **(g)** the TSS structural embedding along the trajectories where the line represent the average and the colored areas (+/-) the standard deviation, **(h)** the average (over the 100 replicates) of the volume explored by the TSS along the TADdyn trajectories every 5 simulation timesteps at each cell stage represented as boxplots (n=100 data points for B and PSC stages, and n=200 data points for the other cell stages) showing: central line, median; box limits, 75th and 25th percentiles; whiskers, 1.5x interquartile range (outliers not shown), **(i-j)** the domains borders on the models contact maps along the entire trajectory (600 time points) **(i)** and the Hi-C interactions maps at each cell stage (7 time points) **(j)**, **(k)** the heat-map showing the average distance to the TSS of each particle along the TADdyn trajectories, and **(l)** the number of active (A), active-proximal (AP), and active-proximal-domain (APD) particle respect to the TSS. Genome tracks for the annotated genes and regulatory elements (promoters, enhancers, and protein binding sites), and for ATAC-seq, CTCF, and H3K4me2 peaks at each reprogramming stage generated in Stadhouders *et al. Nat. Genet.* **50**, 238-249 (2018) are available here for the *Mmp12* simulated region.

Mmp3: chr9:7445821-7455975. Forward

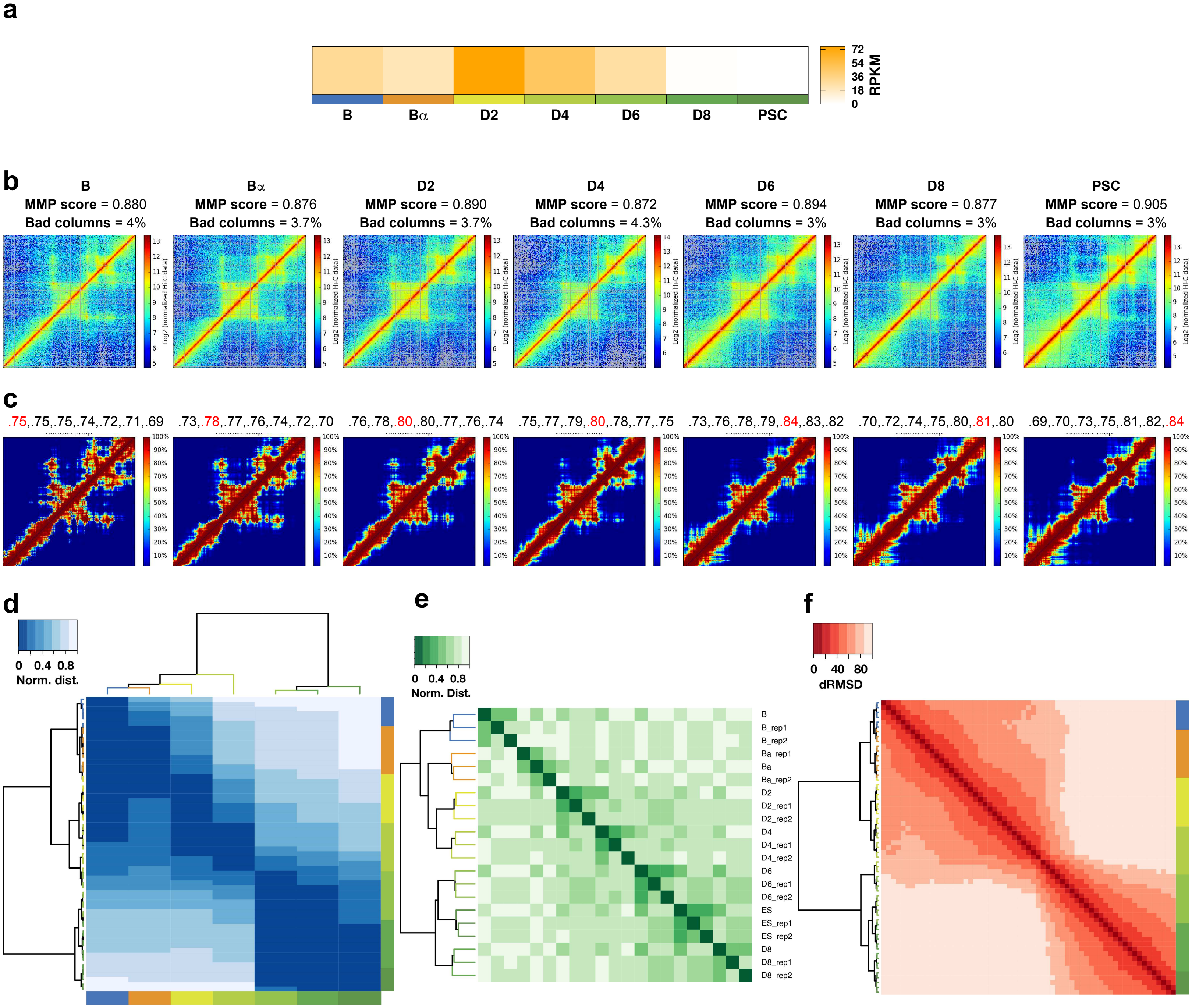

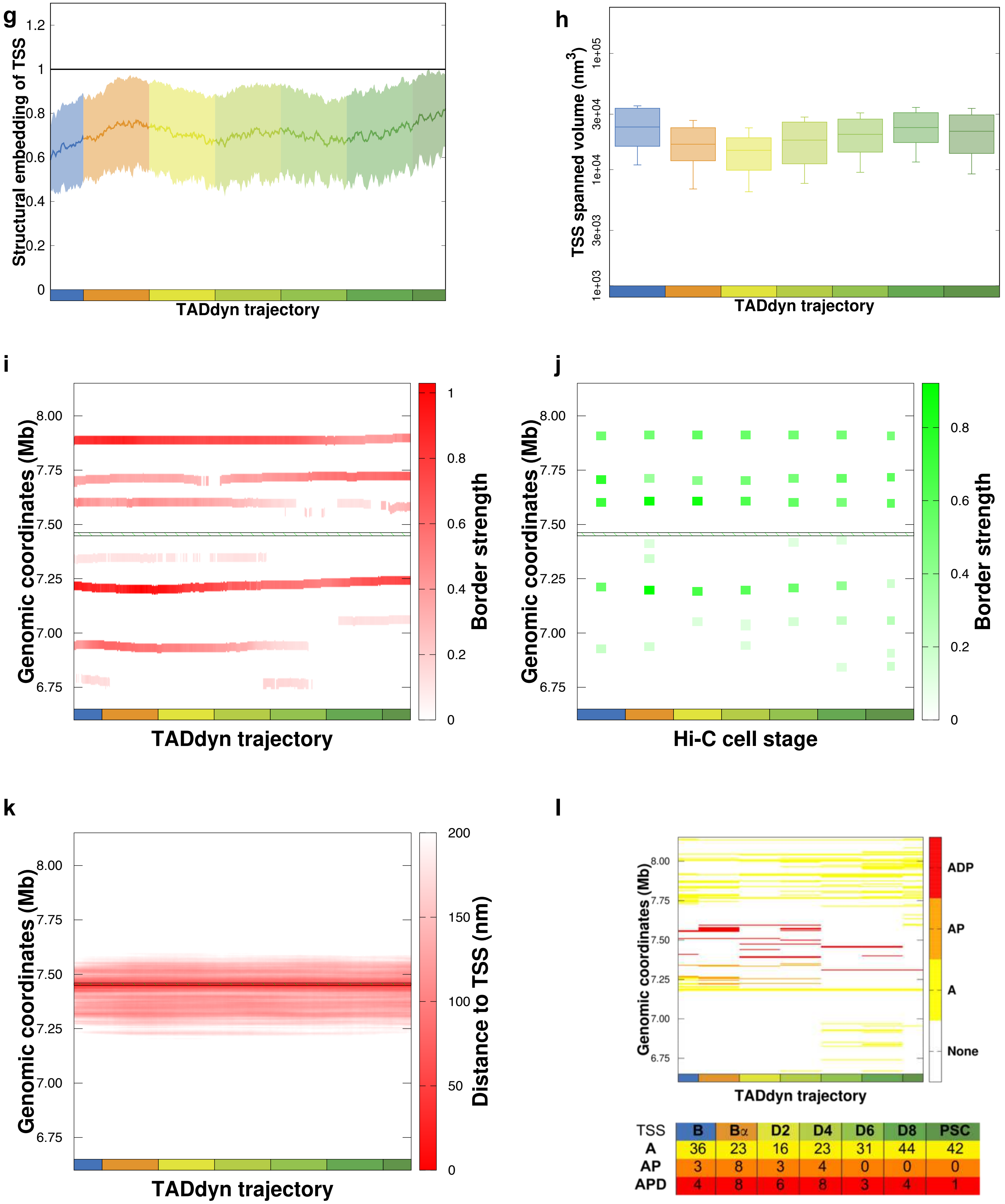

TSS	B	Bα	D2	D4	D6	D8	PSC
A	36	23	16	23	31	44	42
AP	3	8	3	4	0	0	0
APD	4	8	6	8	3	4	1

Supplementary Figure 7. Complete TADdyn analysis of the *Mmp3* simulated locus. The panels present in two pages all the analysis done on the TADdyn models, the Hi-C datasets, the chromatin tracks (ATAC-seq and H3K4me2 peaks), and RNAseq experiments. Specifically, in page 1 we present **(a)** the expression level per cell stage, **(b)** the in-situ Hi-C interaction maps, **(c)** the models' contact maps at dcutoff=200 nm, **(d-f)** the clustering analysis based on models' vs. Hi-C **(d)** and Hi-C vs. Hi-C **(e)** correlations transformed in normalized distances, and models vs. models **(f)** based on structural distance root-mean-squared displacement (dRMSD). On page 2 we show **(g)** the TSS structural embedding along the trajectories where the line represent the average and the colored areas (+/-) the standard deviation, **(h)** the average (over the 100 replicates) of the volume explored by the TSS along the TADdyn trajectories every 5 simulation timesteps at each cell stage represented as boxplots (n=100 data points for B and PSC stages, and n=200 data points for the other cell stages) showing: central line, median; box limits, 75th and 25th percentiles; whiskers, 1.5x interquartile range (outliers not shown), **(i-j)** the domains borders on the models contact maps along the entire trajectory (600 time points) **(i)** and the Hi-C interactions maps at each cell stage (7 time points) **(j)**, **(k)** the heat-map showing the average distance to the TSS of each particle along the TADdyn trajectories, and **(l)** the number of active (A), active-proximal (AP), and active-proximal-domain (APD) particle respect to the TSS. Genome tracks for the annotated genes and regulatory elements (promoters, enhancers, and protein binding sites), and for ATAC-seq, CTCF, and H3K4me2 peaks at each reprogramming stage generated in Stadhouders *et al. Nat. Genet.* **50**, 238-249 (2018) are available here for the *Mmp3* simulated region.

Nanog: chr6:122707564-122714633. Forward

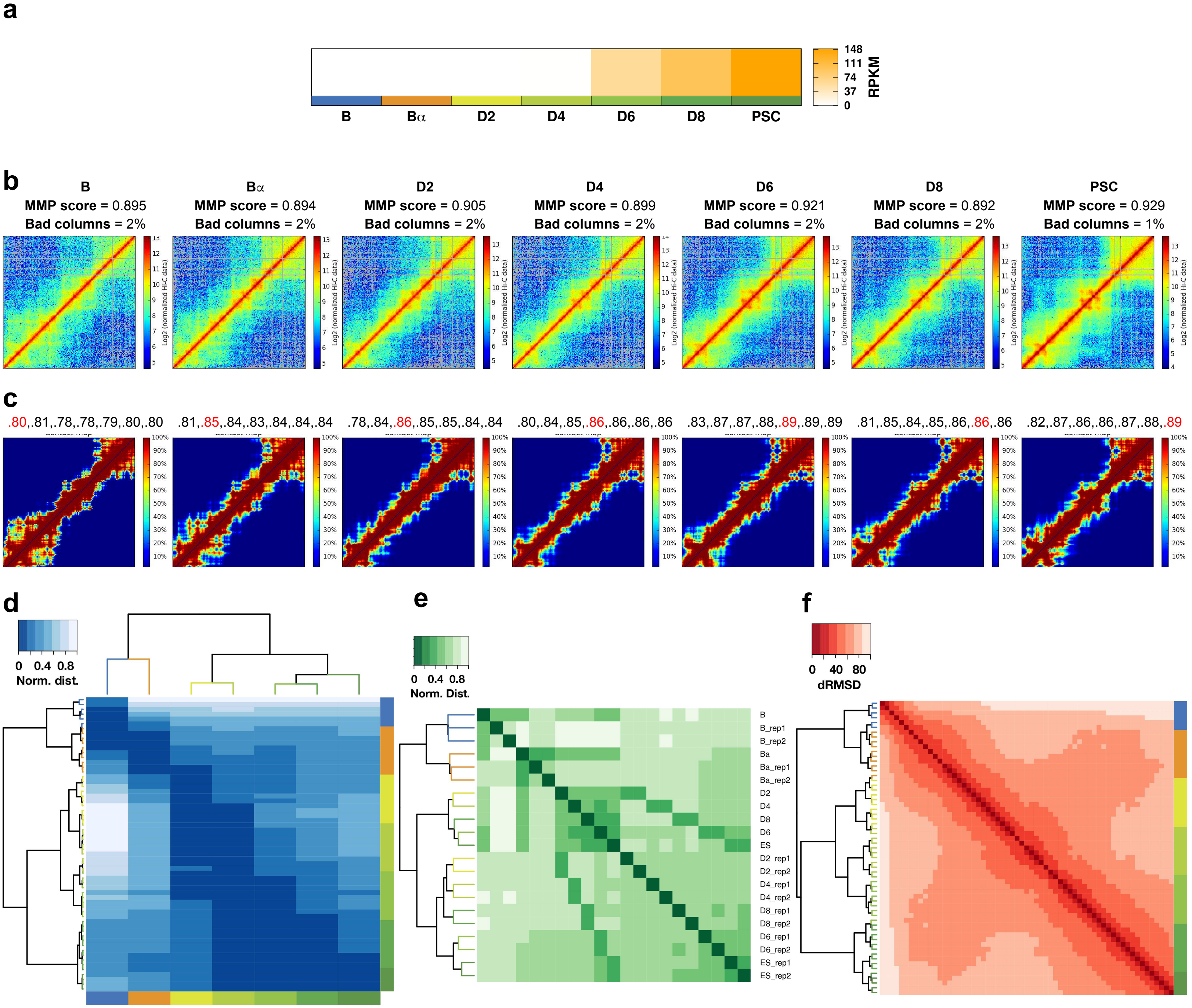

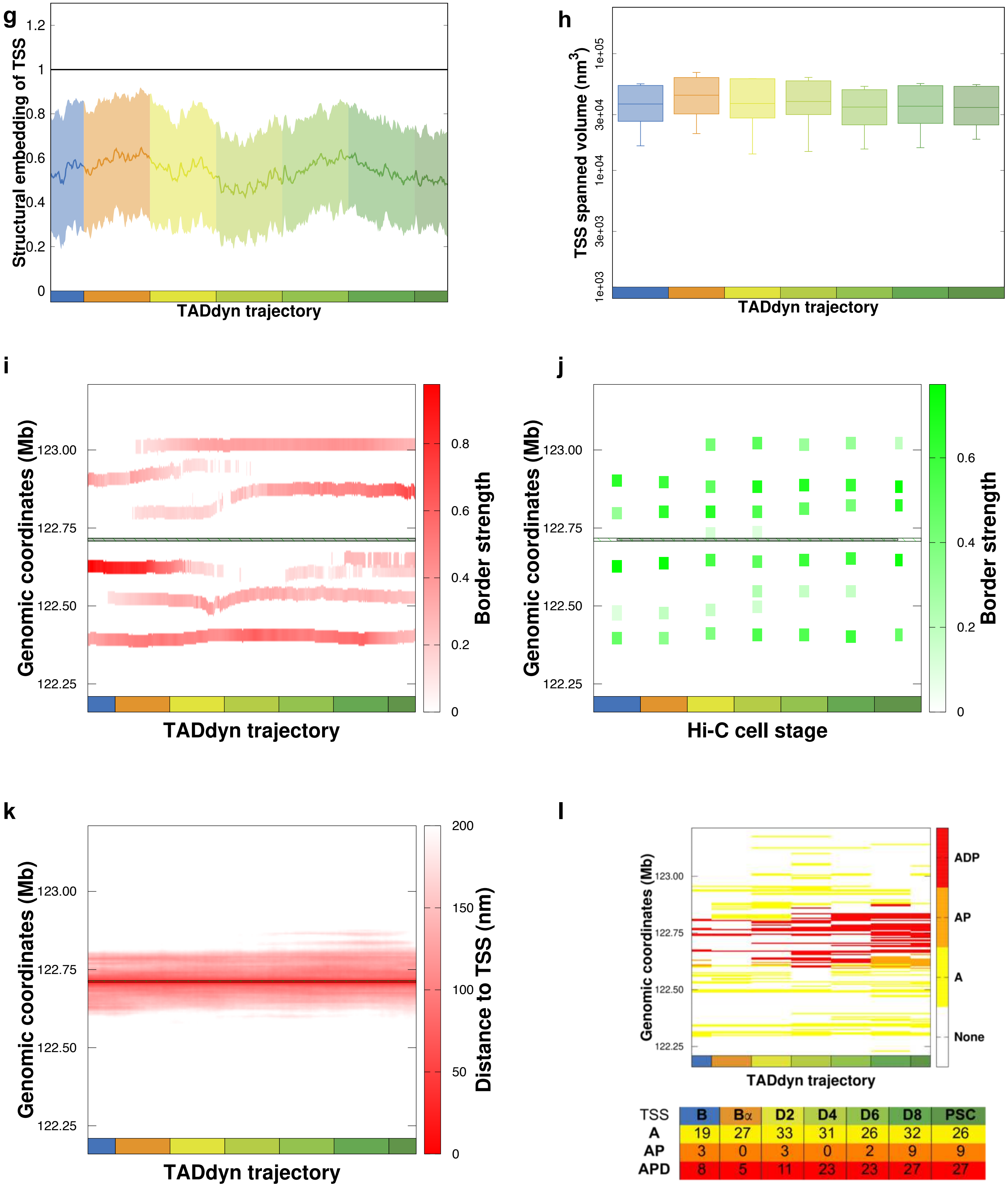

TSS	B	Bα	D2	D4	D6	D8	PSC
A	19	27	33	31	26	32	26
AP	3	0	3	0	2	9	9
APD	8	5	11	23	23	27	27

Supplementary Figure 8. Complete TADdyn analysis of the *Nanog* simulated locus. The panels present in two pages all the analysis done on the TADdyn models, the Hi-C datasets, the chromatin tracks (ATAC-seq and H3K4me2 peaks), and RNAseq experiments. Specifically, in page 1 we present **(a)** the expression level per cell stage, **(b)** the in-situ Hi-C interaction maps, **(c)** the models' contact maps at dcutoff=200 nm, **(d-f)** the clustering analysis based on models' vs. Hi-C **(d)** and Hi-C vs. Hi-C **(e)** correlations transformed in normalized distances, and models vs. models **(f)** based on structural distance root-mean-squared displacement (dRMSD). On page 2 we show **(g)** the TSS structural embedding along the trajectories where the line represent the average and the colored areas (+/-) the standard deviation, **(h)** the average (over the 100 replicates) of the volume explored by the TSS along the TADdyn trajectories every 5 simulation timesteps at each cell stage represented as boxplots (n=100 data points for B and PSC stages, and n=200 data points for the other cell stages) showing: central line, median; box limits, 75th and 25th percentiles; whiskers, 1.5x interquartile range (outliers not shown), **(i-j)** the domains borders on the models contact maps along the entire trajectory (600 time points) **(i)** and the Hi-C interactions maps at each cell stage (7 time points) **(j)**, **(k)** the heat-map showing the average distance to the TSS of each particle along the TADdyn trajectories, and **(l)** the number of active (A), active-proximal (AP), and active-proximal-domain (APD) particle respect to the TSS. Genome tracks for the annotated genes and regulatory elements (promoters, enhancers, and protein binding sites), and for ATAC-seq, CTCF, and H3K4me2 peaks at each reprogramming stage generated in Stadhouders *et al. Nat. Genet.* **50**, 238-249 (2018) are available here for the *Nanog* simulated region.

Neurod6: chr6:55677817-55681263. Reverse

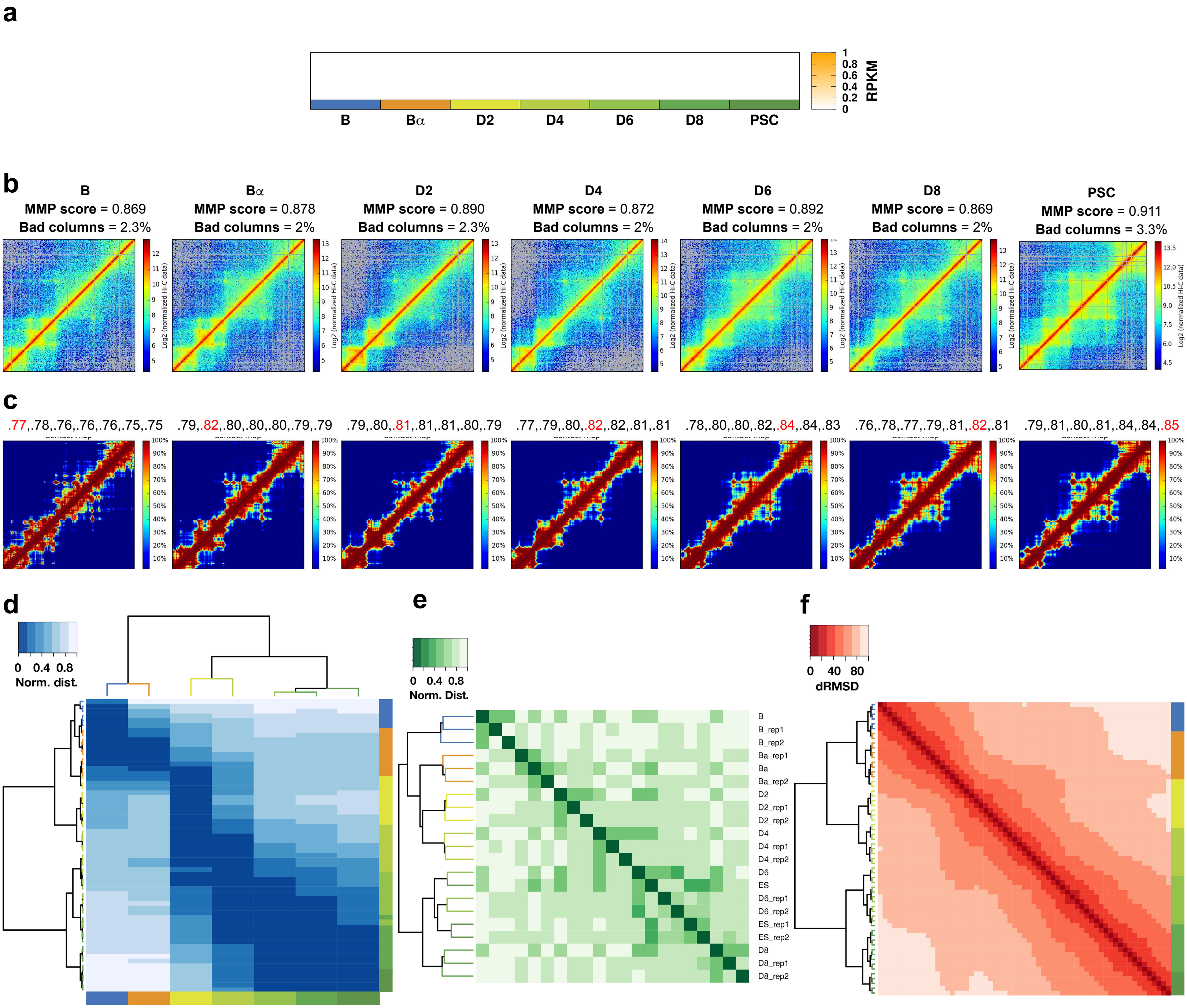

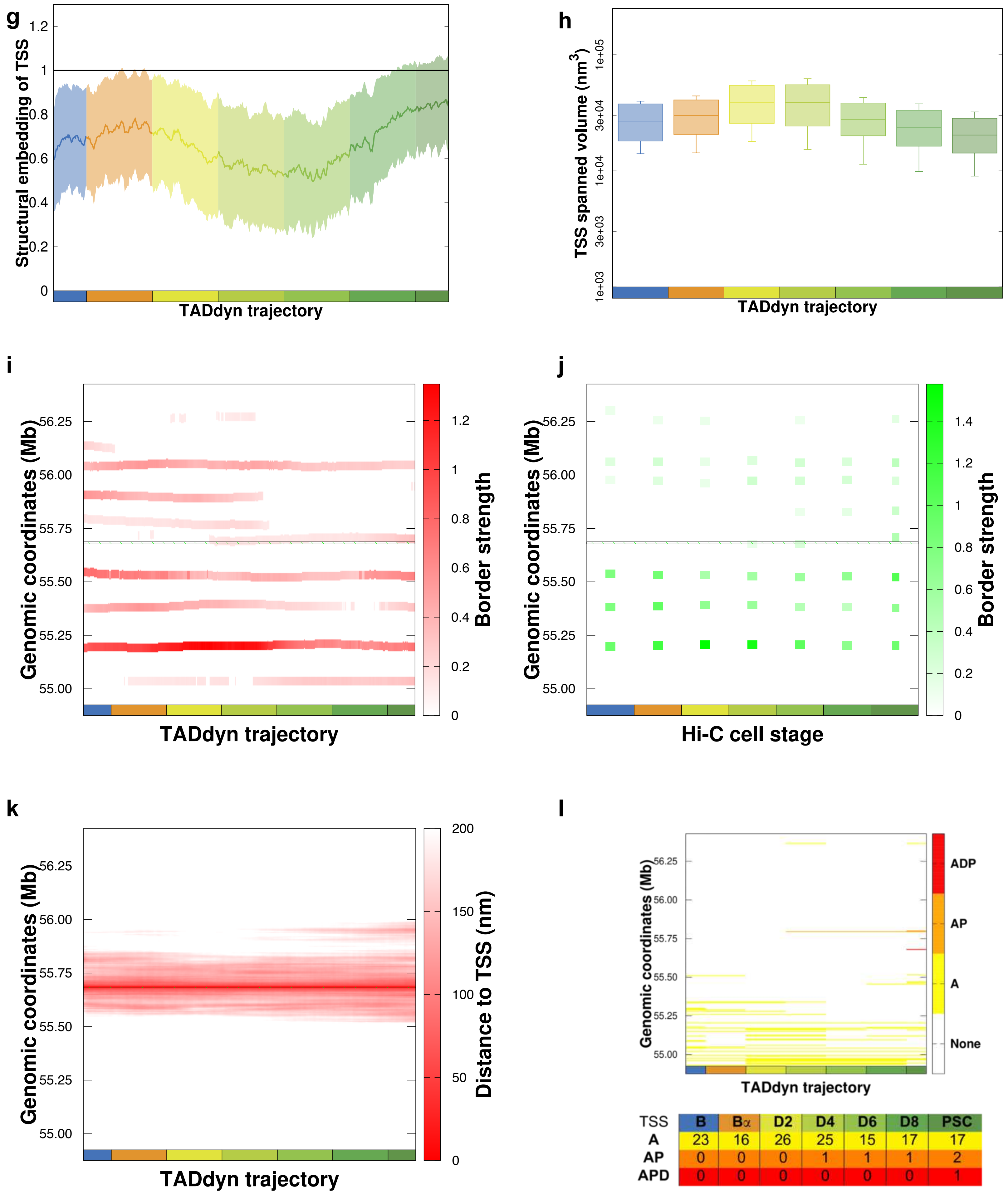

TSS	B	Bα	D2	D4	D6	D8	PSC
A	23	16	26	25	15	17	17
AP	0	0	0	1	1	1	2
APD	0	0	0	0	0	0	1

Supplementary Figure 9. Complete TADdyn analysis of the *Neurod6* simulated locus. The panels present in two pages all the analysis done on the TADdyn models, the Hi-C datasets, the chromatin tracks (ATAC-seq and H3K4me2 peaks), and RNAseq experiments. Specifically, in page 1 we present **(a)** the expression level per cell stage, **(b)** the in-situ Hi-C interaction maps, **(c)** the models' contact maps at dcutoff=200 nm, **(d-f)** the clustering analysis based on models' vs. Hi-C **(d)** and Hi-C vs. Hi-C **(e)** correlations transformed in normalized distances, and models vs. models **(f)** based on structural distance root-mean-squared displacement (dRMSD). On page 2 we show **(g)** the TSS structural embedding along the trajectories where the line represent the average and the colored areas (+/-) the standard deviation, **(h)** the average (over the 100 replicates) of the volume explored by the TSS along the TADdyn trajectories every 5 simulation timesteps at each cell stage represented as boxplots (n=100 data points for B and PSC stages, and n=200 data points for the other cell stages) showing: central line, median; box limits, 75th and 25th percentiles; whiskers, 1.5x interquartile range (outliers not shown), **(i-j)** the domains borders on the models contact maps along the entire trajectory (600 time points) **(i)** and the Hi-C interactions maps at each cell stage (7 time points) **(j)**, **(k)** the heat-map showing the average distance to the TSS of each particle along the TADdyn trajectories, and **(l)** the number of active (A), active-proximal (AP), and active-proximal-domain (APD) particle respect to the TSS. Genome tracks for the annotated genes and regulatory elements (promoters, enhancers, and protein binding sites), and for ATAC-seq, CTCF, and H3K4me2 peaks at each reprogramming stage generated in Stadhouders *et al. Nat. Genet.* **50**, 238-249 (2018) are available here for the *Neurod6* simulated region.

Nos1ap: chr1:170317495-170589849. Reverse

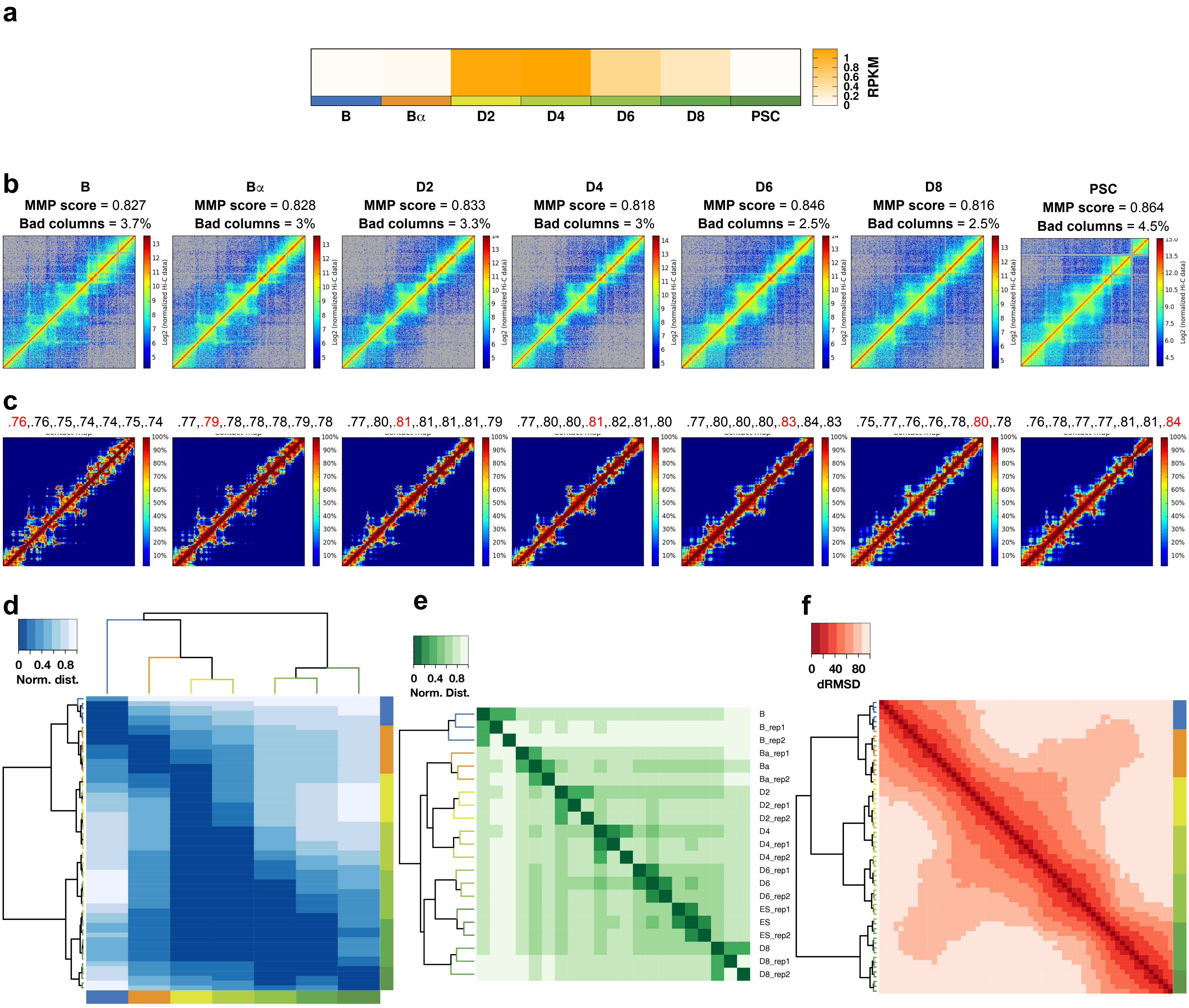

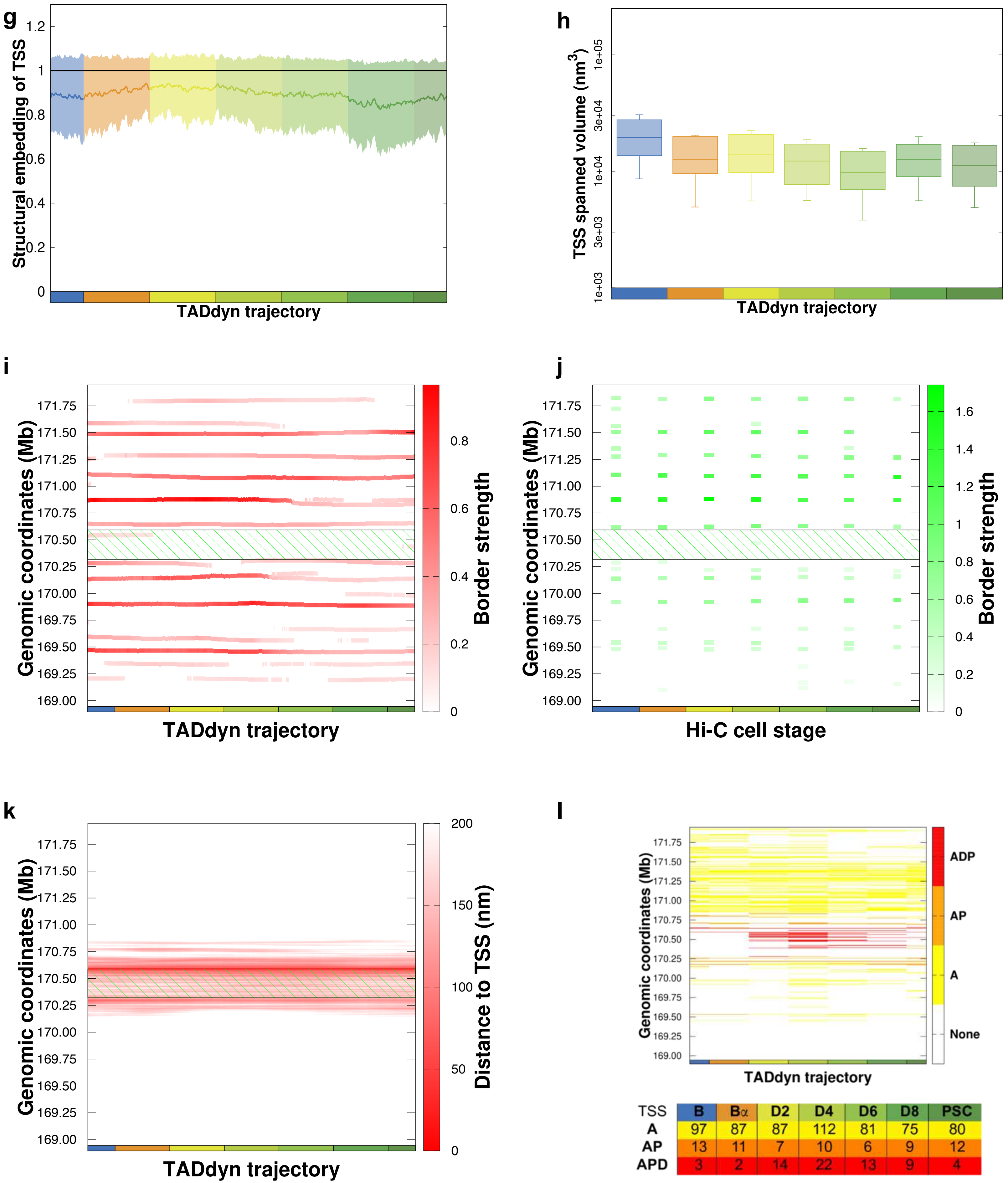

TSS	B	Bα	D2	D4	D6	D8	PSC
A	97	87	87	112	81	75	80
AP	13	11	7	10	6	9	12
APD	3	2	14	22	13	9	4

Supplementary Figure 10. Complete TADdyn analysis of the *Nos1ap* simulated locus. The panels present in two pages all the analysis done on the TADdyn models, the Hi-C datasets, the chromatin tracks (ATAC-seq and H3K4me2 peaks), and RNAseq experiments. Specifically, in page 1 we present **(a)** the expression level per cell stage, **(b)** the in-situ Hi-C interaction maps, **(c)** the models' contact maps at dcutoff=200 nm, **(d-f)** the clustering analysis based on models' vs. Hi-C **(d)** and Hi-C vs. Hi-C **(e)** correlations transformed in normalized distances, and models vs. models **(f)** based on structural distance root-mean-squared displacement (dRMSD). On page 2 we show **(g)** the TSS structural embedding along the trajectories where the line represent the average and the colored areas (+/-) the standard deviation, **(h)** the average (over the 100 replicates) of the volume explored by the TSS along the TADdyn trajectories every 5 simulation timesteps at each cell stage represented as boxplots (n=100 data points for B and PSC stages, and n=200 data points for the other cell stages) showing: central line, median; box limits, 75th and 25th percentiles; whiskers, 1.5x interquartile range (outliers not shown), **(i-j)** the domains borders on the models contact maps along the entire trajectory (600 time points) **(i)** and the Hi-C interactions maps at each cell stage (7 time points) **(j)**, **(k)** the heat-map showing the average distance to the TSS of each particle along the TADdyn trajectories, and **(l)** the number of active (A), active-proximal (AP), and active-proximal-domain (APD) particle respect to the TSS. Genome tracks for the annotated genes and regulatory elements (promoters, enhancers, and protein binding sites), and for ATAC-seq, CTCF, and H3K4me2 peaks at each reprogramming stage generated in Stadhouders *et al. Nat. Genet.* **50**, 238-249 (2018) are available here for the *Nos1ap* simulated region.

Olfr1002: chr2:85647362-85648319. Reverse

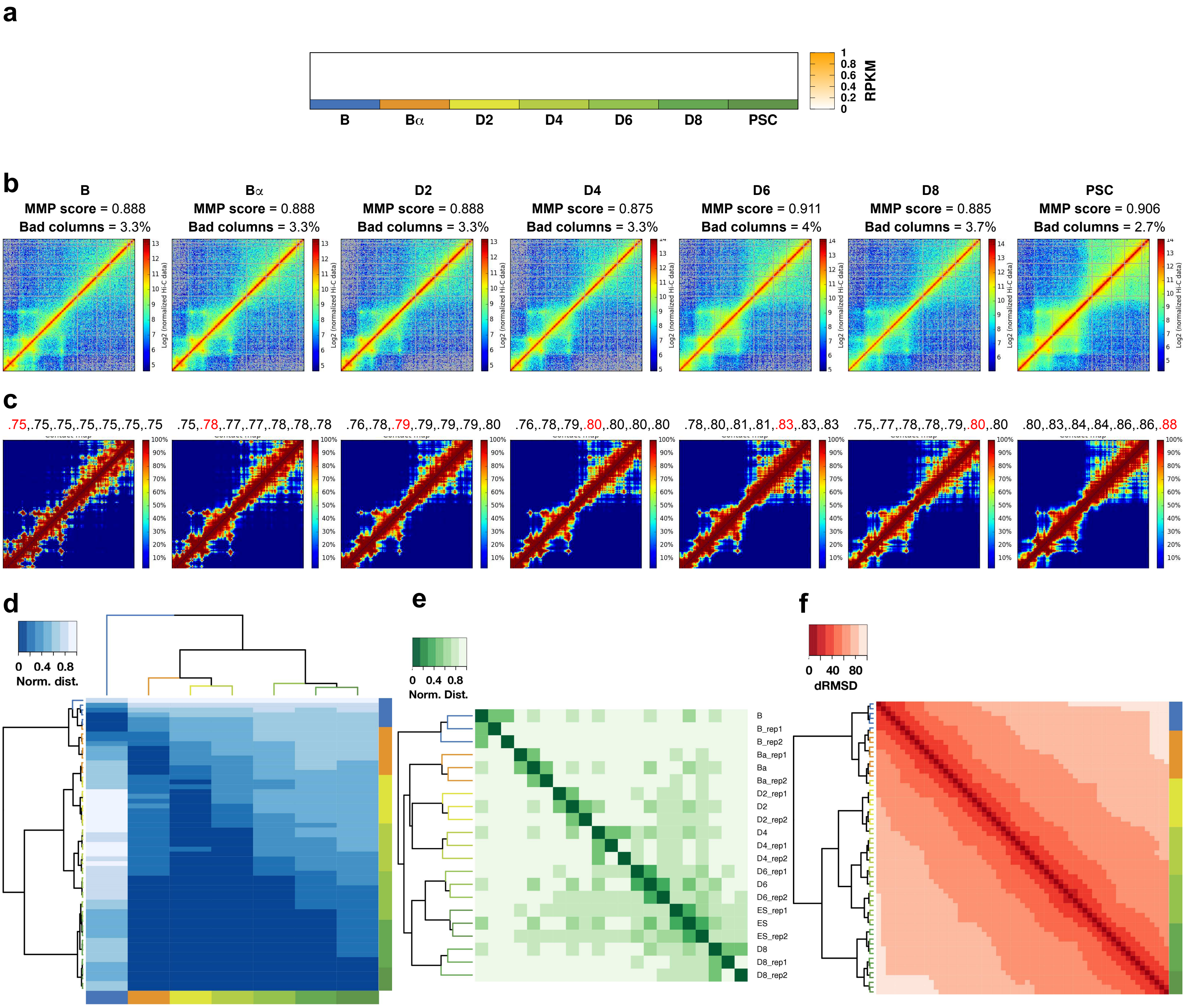

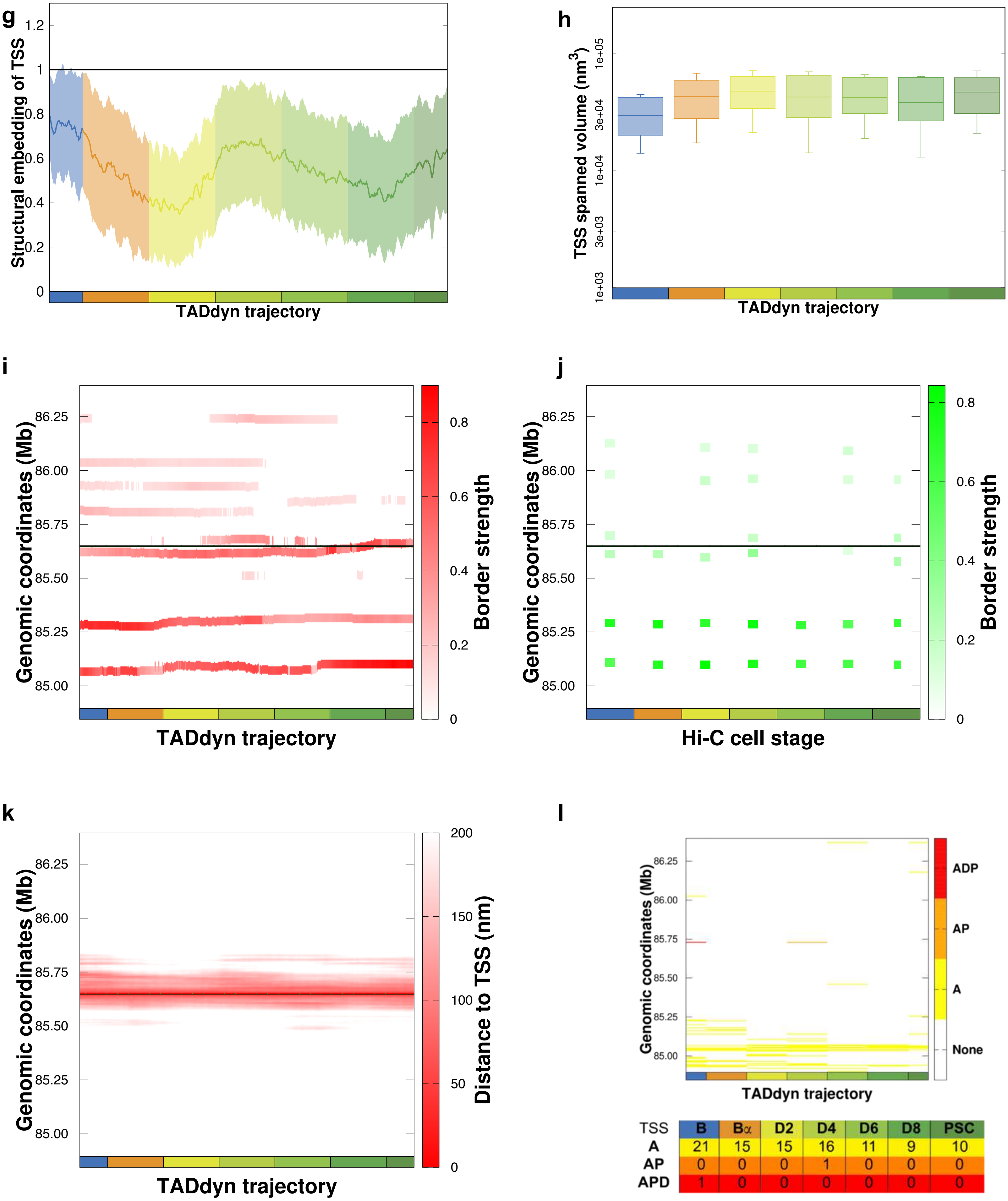

TSS	B	Bα	D2	D4	D6	D8	PSC
A	21	15	15	16	11	9	10
AP	0	0	0	1	0	0	0
APD	1	0	0	0	0	0	0

Supplementary Figure 11. Complete TADdyn analysis of the *Olfr1002* simulated locus. The panels present in two pages all the analysis done on the TADdyn models, the Hi-C datasets, the chromatin tracks (ATAC-seq and H3K4me2 peaks), and RNAseq experiments. Specifically, in page 1 we present **(a)** the expression level per cell stage, **(b)** the in-situ Hi-C interaction maps, **(c)** the models' contact maps at dcutoff=200 nm, **(d-f)** the clustering analysis based on models' vs. Hi-C **(d)** and Hi-C vs. Hi-C **(e)** correlations transformed in normalized distances, and models vs. models **(f)** based on structural distance root-mean-squared displacement (dRMSD). On page 2 we show **(g)** the TSS structural embedding along the trajectories where the line represent the average and the colored areas (+/-) the standard deviation, **(h)** the average (over the 100 replicates) of the volume explored by the TSS along the TADdyn trajectories every 5 simulation timesteps at each cell stage represented as boxplots (n=100 data points for B and PSC stages, and n=200 data points for the other cell stages) showing: central line, median; box limits, 75th and 25th percentiles; whiskers, 1.5x interquartile range (outliers not shown), **(i-j)** the domains borders on the models contact maps along the entire trajectory (600 time points) **(i)** and the Hi-C interactions maps at each cell stage (7 time points) **(j)**, **(k)** the heat-map showing the average distance to the TSS of each particle along the TADdyn trajectories, and **(l)** the number of active (A), active-proximal (AP), and active-proximal-domain (APD) particle respect to the TSS. Genome tracks for the annotated genes and regulatory elements (promoters, enhancers, and protein binding sites), and for ATAC-seq, CTCF, and H3K4me2 peaks at each reprogramming stage generated in Stadhouders *et al. Nat. Genet.* **50**, 238-249 (2018) are available here for the *Olfr1002* simulated region.

Olfr33: chr7:102713454-102714411. Reverse

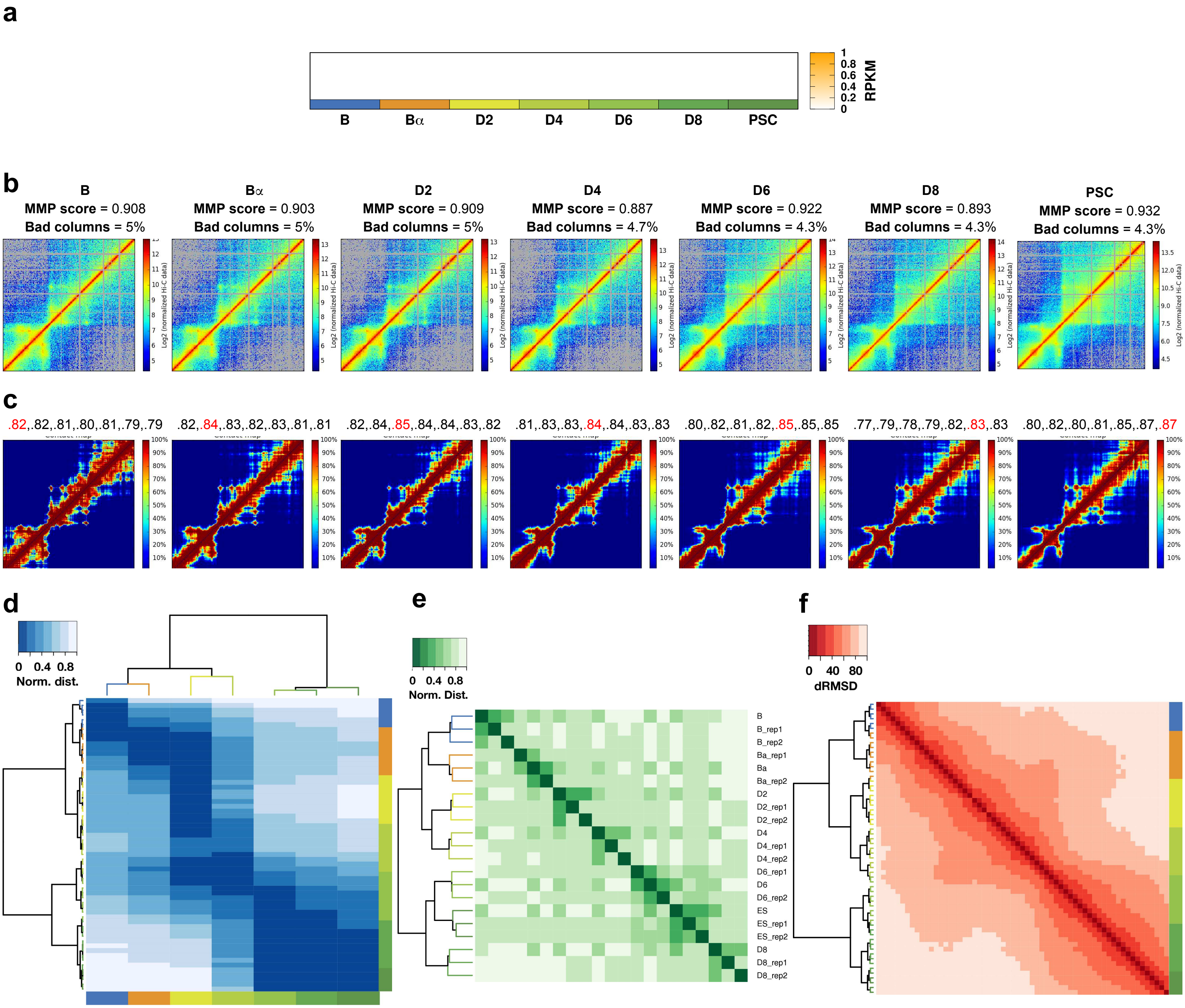

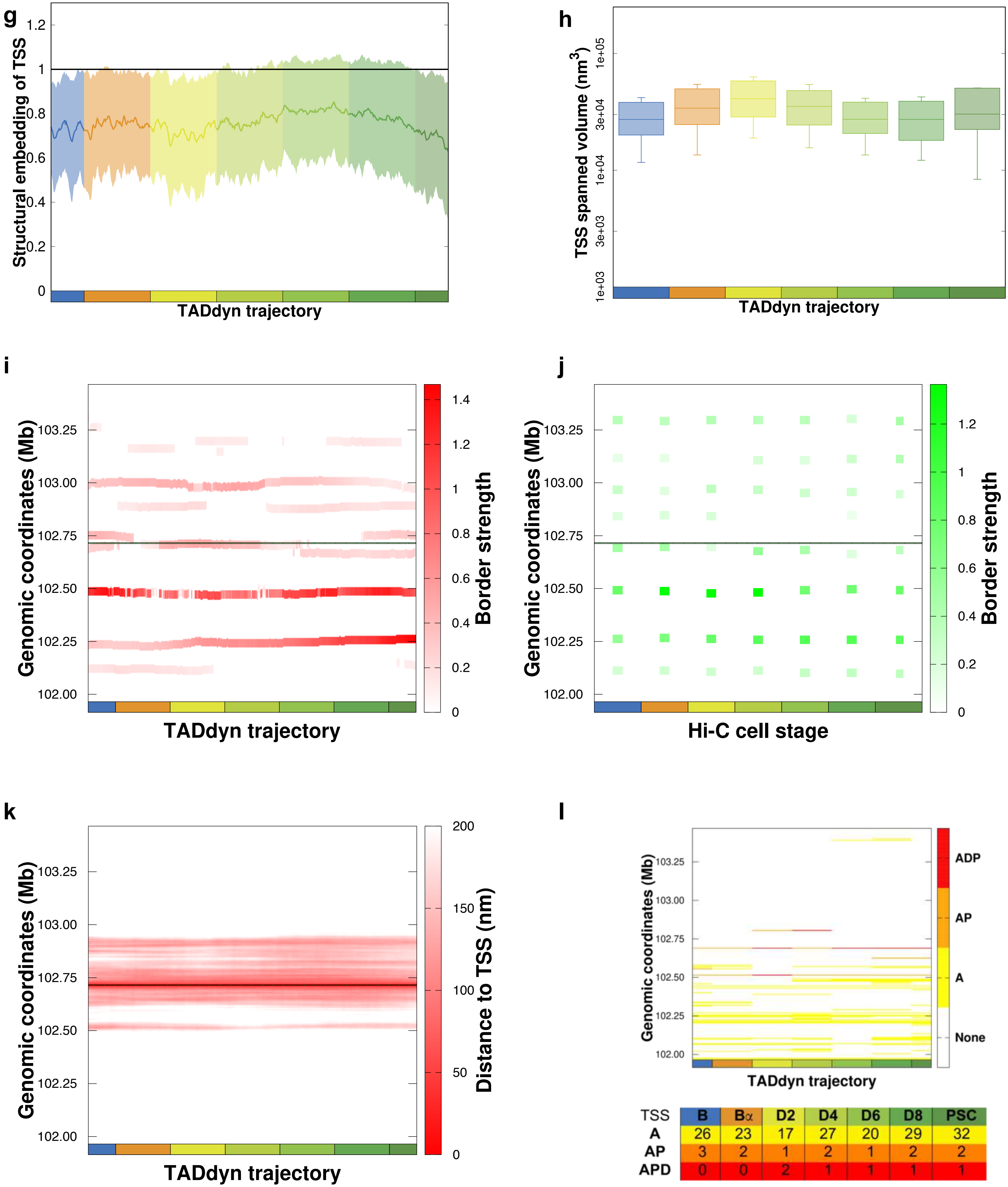

TSS	B	Bα	D2	D4	D6	D8	PSC
A	26	23	17	27	20	29	32
AP	3	2	1	2	1	2	2
APD	0	0	2	1	1	1	1

Supplementary Figure 12. Complete TADdyn analysis of the *Olfr33* simulated locus. The panels present in two pages all the analysis done on the TADdyn models, the Hi-C datasets, the chromatin tracks (ATAC-seq and H3K4me2 peaks), and RNAseq experiments. Specifically, in page 1 we present **(a)** the expression level per cell stage, **(b)** the in-situ Hi-C interaction maps, **(c)** the models' contact maps at dcutoff=200 nm, **(d-f)** the clustering analysis based on models' vs. Hi-C **(d)** and Hi-C vs. Hi-C **(e)** correlations transformed in normalized distances, and models vs. models **(f)** based on structural distance root-mean-squared displacement (dRMSD). On page 2 we show **(g)** the TSS structural embedding along the trajectories where the line represent the average and the colored areas (+/-) the standard deviation, **(h)** the average (over the 100 replicates) of the volume explored by the TSS along the TADdyn trajectories every 5 simulation timesteps at each cell stage represented as boxplots (n=100 data points for B and PSC stages, and n=200 data points for the other cell stages) showing: central line, median; box limits, 75th and 25th percentiles; whiskers, 1.5x interquartile range (outliers not shown), **(i-j)** the domains borders on the models contact maps along the entire trajectory (600 time points) **(i)** and the Hi-C interactions maps at each cell stage (7 time points) **(j)**, **(k)** the heat-map showing the average distance to the TSS of each particle along the TADdyn trajectories, and **(l)** the number of active (A), active-proximal (AP), and active-proximal-domain (APD) particle respect to the TSS. Genome tracks for the annotated genes and regulatory elements (promoters, enhancers, and protein binding sites), and for ATAC-seq, CTCF, and H3K4me2 peaks at each reprogramming stage generated in Stadhouders *et al. Nat. Genet.* **50**, 238-249 (2018) are available here for the *Olfr33* simulated region.

Ppia: chr11:6415869-6419810. Forward

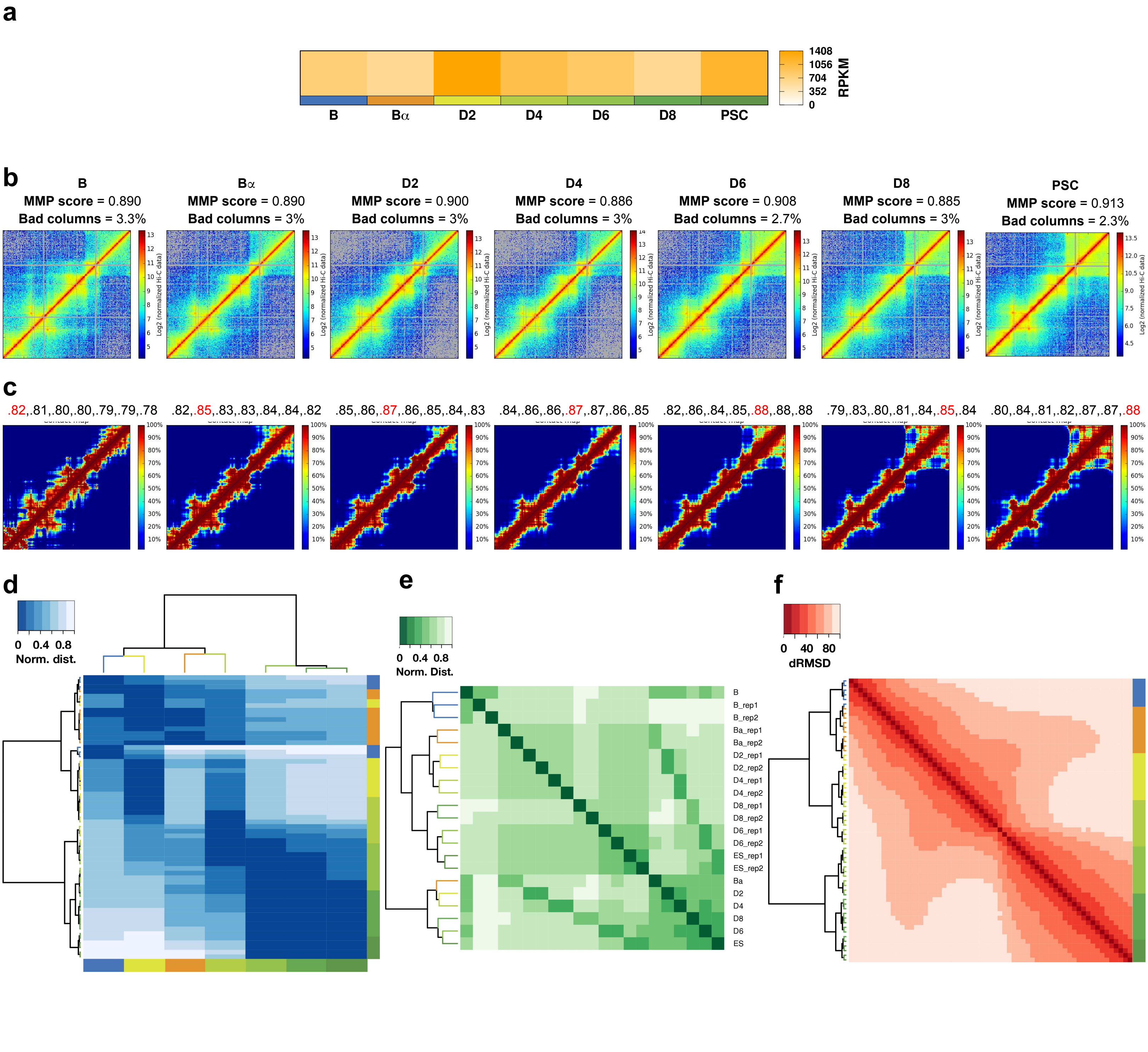

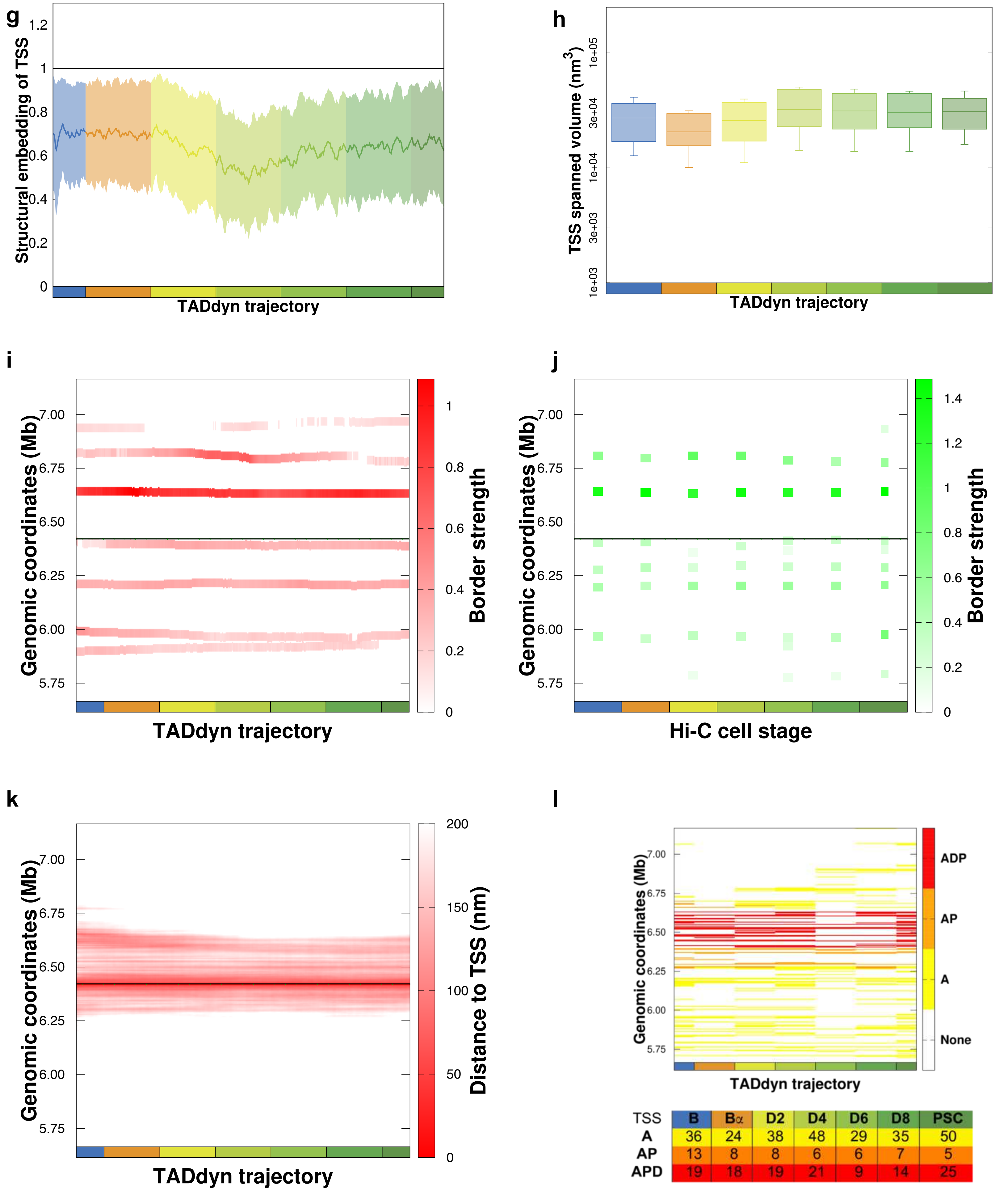

TSS	B	Bα	D2	D4	D6	D8	PSC
A	36	24	38	48	29	35	50
AP	13	8	8	6	6	7	5
APD	19	18	19	21	9	14	25

Supplementary Figure 13. Complete TADdyn analysis of the *Ppia* simulated locus. The panels present in two pages all the analysis done on the TADdyn models, the Hi-C datasets, the chromatin tracks (ATAC-seq and H3K4me2 peaks), and RNAseq experiments. Specifically, in page 1 we present **(a)** the expression level per cell stage, **(b)** the in-situ Hi-C interaction maps, **(c)** the models' contact maps at dcutoff=200 nm, **(d-f)** the clustering analysis based on models' vs. Hi-C **(d)** and Hi-C vs. Hi-C **(e)** correlations transformed in normalized distances, and models vs. models **(f)** based on structural distance root-mean-squared displacement (dRMSD). On page 2 we show **(g)** the TSS structural embedding along the trajectories where the line represent the average and the colored areas (+/-) the standard deviation, **(h)** the average (over the 100 replicates) of the volume explored by the TSS along the TADdyn trajectories every 5 simulation timesteps at each cell stage represented as boxplots (n=100 data points for B and PSC stages, and n=200 data points for the other cell stages) showing: central line, median; box limits, 75th and 25th percentiles; whiskers, 1.5x interquartile range (outliers not shown), **(i-j)** the domains borders on the models contact maps along the entire trajectory (600 time points) **(i)** and the Hi-C interactions maps at each cell stage (7 time points) **(j)**, **(k)** the heat-map showing the average distance to the TSS of each particle along the TADdyn trajectories, and **(l)** the number of active (A), active-proximal (AP), and active-proximal-domain (APD) particle respect to the TSS. Genome tracks for the annotated genes and regulatory elements (promoters, enhancers, and protein binding sites), and for ATAC-seq, CTCF, and H3K4me2 peaks at each reprogramming stage generated in Stadhouders *et al. Nat. Genet.* **50**, 238-249 (2018) are available here for the *Ppia* simulated region.

Rad23a: chr8:84834651-84840665. Reverse

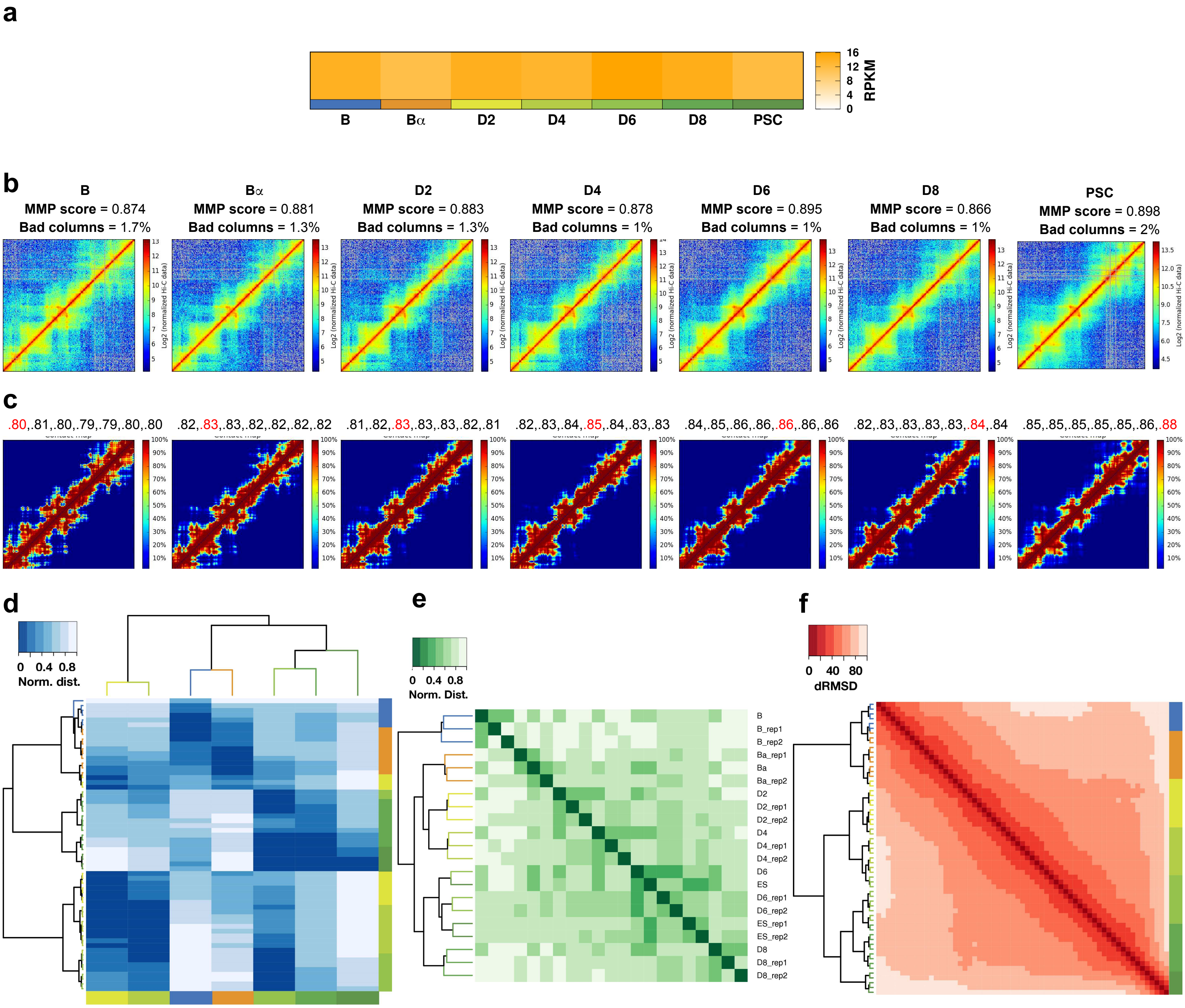

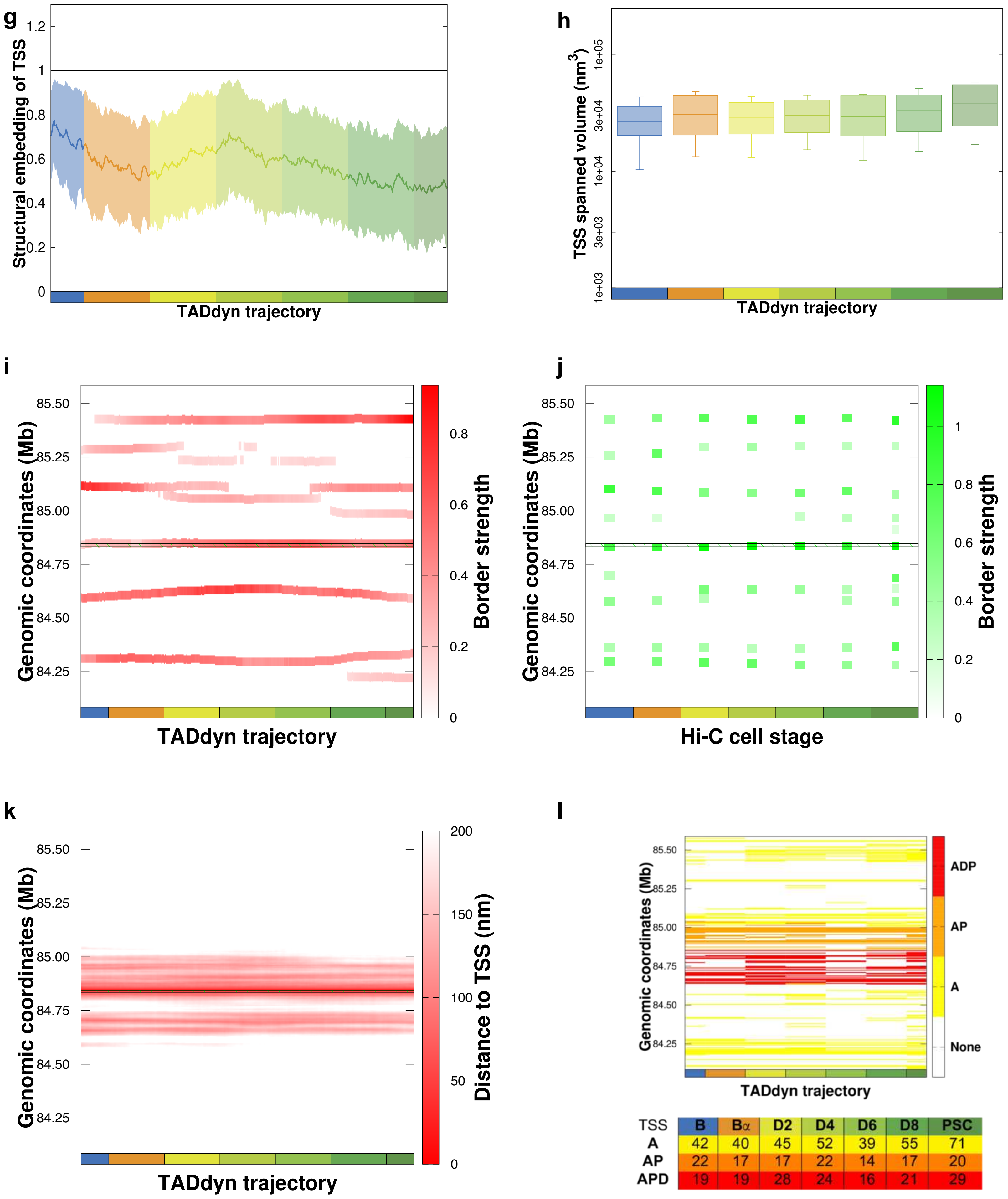

TSS	B	Bα	D2	D4	D6	D8	PSC
A	42	40	45	52	39	55	71
AP	22	17	17	22	14	17	20
APD	19	19	28	24	16	21	29

Supplementary Figure 14. Complete TADdyn analysis of the *Rad23a* simulated locus. The panels present in two pages all the analysis done on the TADdyn models, the Hi-C datasets, the chromatin tracks (ATAC-seq and H3K4me2 peaks), and RNAseq experiments. Specifically, in page 1 we present **(a)** the expression level per cell stage, **(b)** the in-situ Hi-C interaction maps, **(c)** the models' contact maps at dcutoff=200 nm, **(d-f)** the clustering analysis based on models' vs. Hi-C **(d)** and Hi-C vs. Hi-C **(e)** correlations transformed in normalized distances, and models vs. models **(f)** based on structural distance root-mean-squared displacement (dRMSD). On page 2 we show **(g)** the TSS structural embedding along the trajectories where the line represent the average and the colored areas (+/-) the standard deviation, **(h)** the average (over the 100 replicates) of the volume explored by the TSS along the TADdyn trajectories every 5 simulation timesteps at each cell stage represented as boxplots (n=100 data points for B and PSC stages, and n=200 data points for the other cell stages) showing: central line, median; box limits, 75th and 25th percentiles; whiskers, 1.5x interquartile range (outliers not shown), **(i-j)** the domains borders on the models contact maps along the entire trajectory (600 time points) **(i)** and the Hi-C interactions maps at each cell stage (7 time points) **(j)**, **(k)** the heat-map showing the average distance to the TSS of each particle along the TADdyn trajectories, and **(l)** the number of active (A), active-proximal (AP), and active-proximal-domain (APD) particle respect to the TSS. Genome tracks for the annotated genes and regulatory elements (promoters, enhancers, and protein binding sites), and for ATAC-seq, CTCF, and H3K4me2 peaks at each reprogramming stage generated in Stadhouders *et al. Nat. Genet.* **50**, 238-249 (2018) are available here for the *Rad23a* simulated region.

Rad23b: chr4:55350041-55392237. Forward

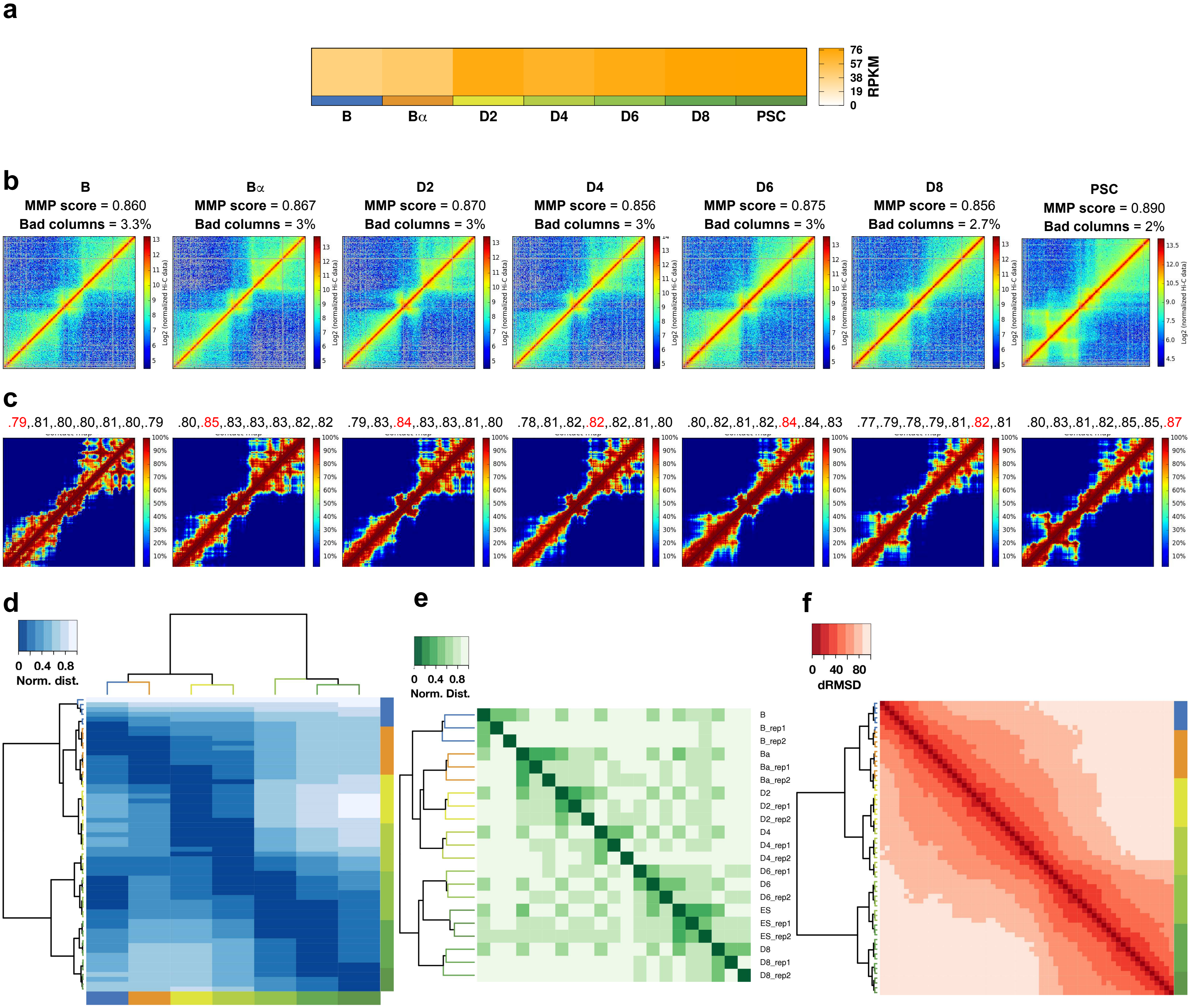

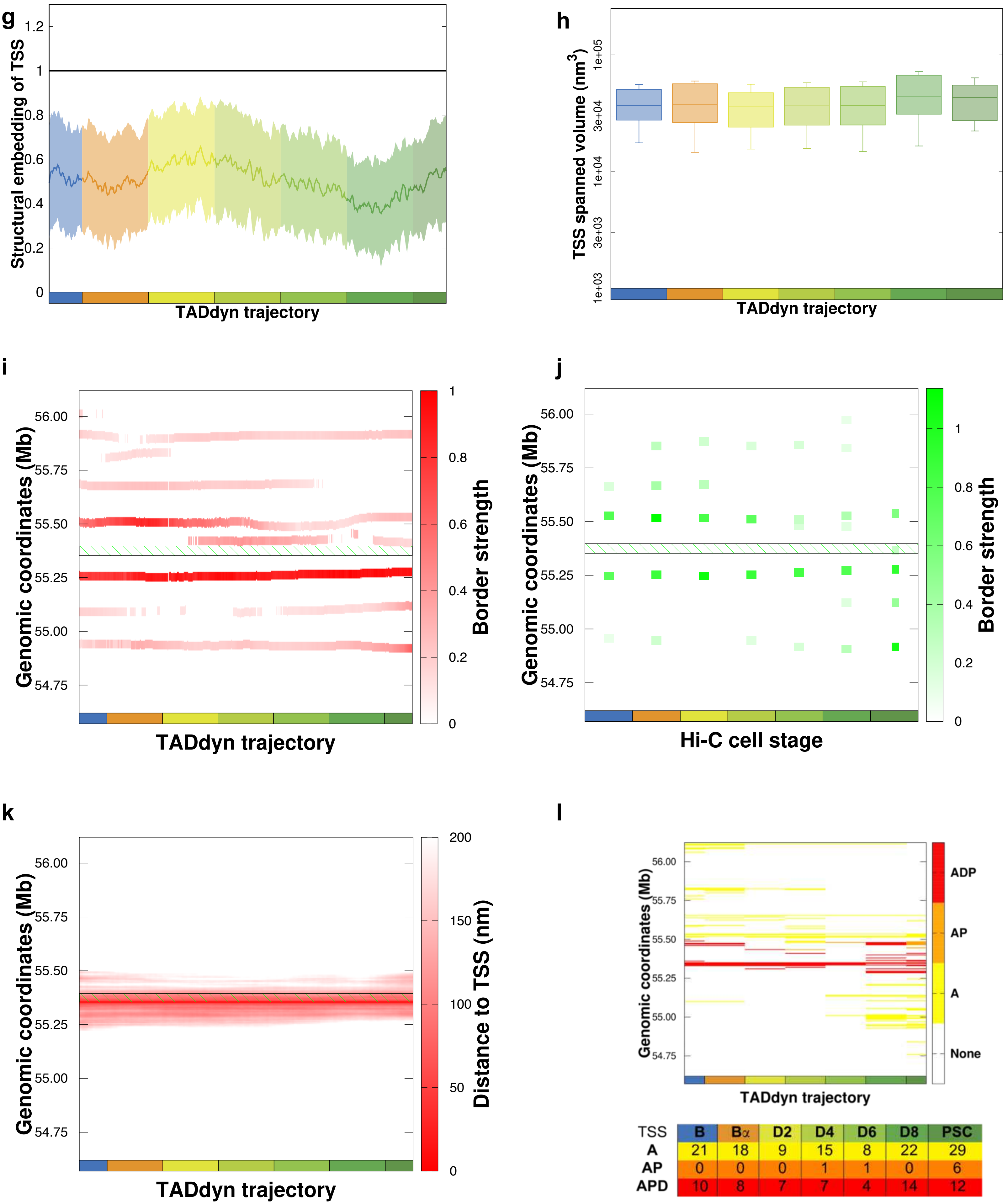

TSS	B	Bα	D2	D4	D6	D8	PSC
A	21	18	9	15	8	22	29
AP	0	0	0	1	1	0	6
APD	10	8	7	7	4	14	12

Supplementary Figure 15. Complete TADdyn analysis of the *Rad23b* simulated locus. The panels present in two pages all the analysis done on the TADdyn models, the Hi-C datasets, the chromatin tracks (ATAC-seq and H3K4me2 peaks), and RNAseq experiments. Specifically, in page 1 we present **(a)** the expression level per cell stage, **(b)** the in-situ Hi-C interaction maps, **(c)** the models' contact maps at dcutoff=200 nm, **(d-f)** the clustering analysis based on models' vs. Hi-C **(d)** and Hi-C vs. Hi-C **(e)** correlations transformed in normalized distances, and models vs. models **(f)** based on structural distance root-mean-squared displacement (dRMSD). On page 2 we show **(g)** the TSS structural embedding along the trajectories where the line represent the average and the colored areas (+/-) the standard deviation, **(h)** the average (over the 100 replicates) of the volume explored by the TSS along the TADdyn trajectories every 5 simulation timesteps at each cell stage represented as boxplots (n=100 data points for B and PSC stages, and n=200 data points for the other cell stages) showing: central line, median; box limits, 75th and 25th percentiles; whiskers, 1.5x interquartile range (outliers not shown), **(i-j)** the domains borders on the models contact maps along the entire trajectory (600 time points) **(i)** and the Hi-C interactions maps at each cell stage (7 time points) **(j)**, **(k)** the heat-map showing the average distance to the TSS of each particle along the TADdyn trajectories, and **(l)** the number of active (A), active-proximal (AP), and active-proximal-domain (APD) particle respect to the TSS. Genome tracks for the annotated genes and regulatory elements (promoters, enhancers, and protein binding sites), and for ATAC-seq, CTCF, and H3K4me2 peaks at each reprogramming stage generated in Stadhouders *et al. Nat. Genet.* **50**, 238-249 (2018) are available here for the *Rad23b* simulated region.

Rergl: chr6:139493181-139501909. Reverse

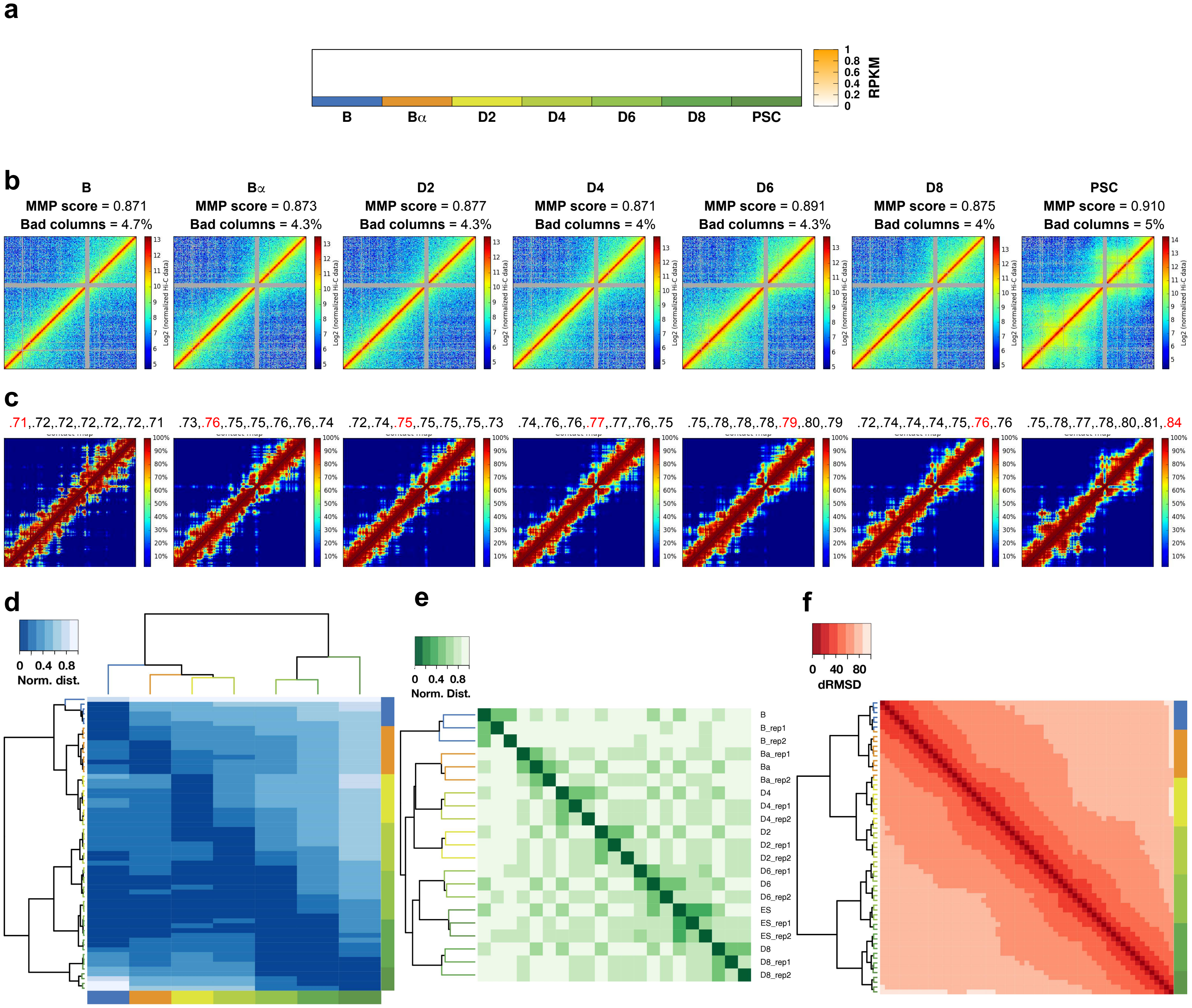

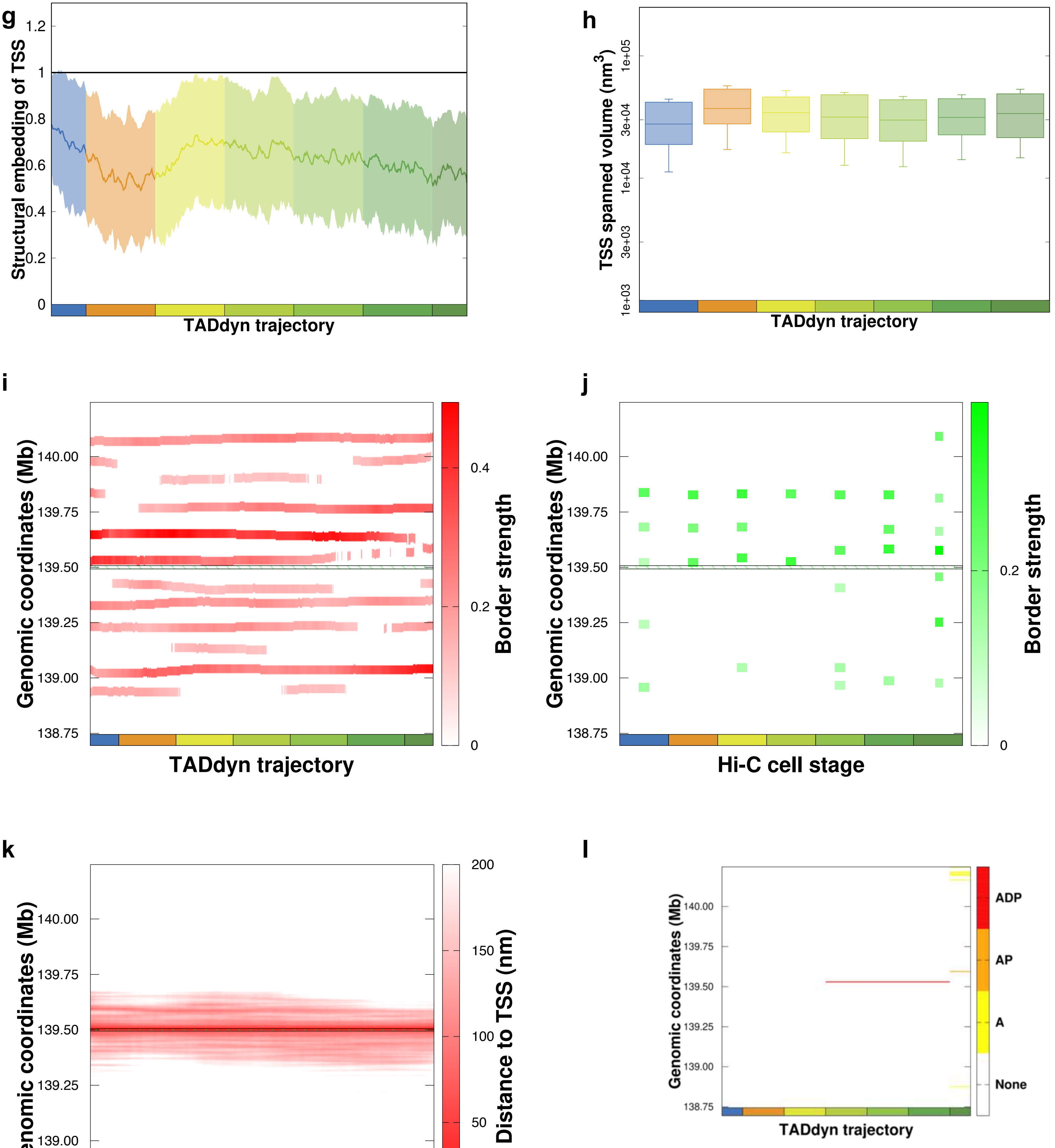

TSS	B	Bα	D2	D4	D6	D8	PSC
A	0	0	0	0	0	0	7
AP	0	0	0	0	0	0	1
APD	0	0	0	1	1	1	0

Supplementary Figure 16. Complete TADdyn analysis of the *Rergl* simulated locus. The panels present in two pages all the analysis done on the TADdyn models, the Hi-C datasets, the chromatin tracks (ATAC-seq and H3K4me2 peaks), and RNAseq experiments. Specifically, in page 1 we present **(a)** the expression level per cell stage, **(b)** the in-situ Hi-C interaction maps, **(c)** the models' contact maps at dcutoff=200 nm, **(d-f)** the clustering analysis based on models' vs. Hi-C **(d)** and Hi-C vs. Hi-C **(e)** correlations transformed in normalized distances, and models vs. models **(f)** based on structural distance root-mean-squared displacement (dRMSD). On page 2 we show **(g)** the TSS structural embedding along the trajectories where the line represent the average and the colored areas (+/-) the standard deviation, **(h)** the average (over the 100 replicates) of the volume explored by the TSS along the TADdyn trajectories every 5 simulation timesteps at each cell stage represented as boxplots (n=100 data points for B and PSC stages, and n=200 data points for the other cell stages) showing: central line, median; box limits, 75th and 25th percentiles; whiskers, 1.5x interquartile range (outliers not shown), **(i-j)** the domains borders on the models contact maps along the entire trajectory (600 time points) **(i)** and the Hi-C interactions maps at each cell stage (7 time points) **(j)**, **(k)** the heat-map showing the average distance to the TSS of each particle along the TADdyn trajectories, and **(l)** the number of active (A), active-proximal (AP), and active-proximal-domain (APD) particle respect to the TSS. Genome tracks for the annotated genes and regulatory elements (promoters, enhancers, and protein binding sites), and for ATAC-seq, CTCF, and H3K4me2 peaks at each reprogramming stage generated in Stadhouders *et al. Nat. Genet.* **50**, 238-249 (2018) are available here for the *Rergl* simulated region.

Rnu7: chr6:124741224-124741286. Reverse

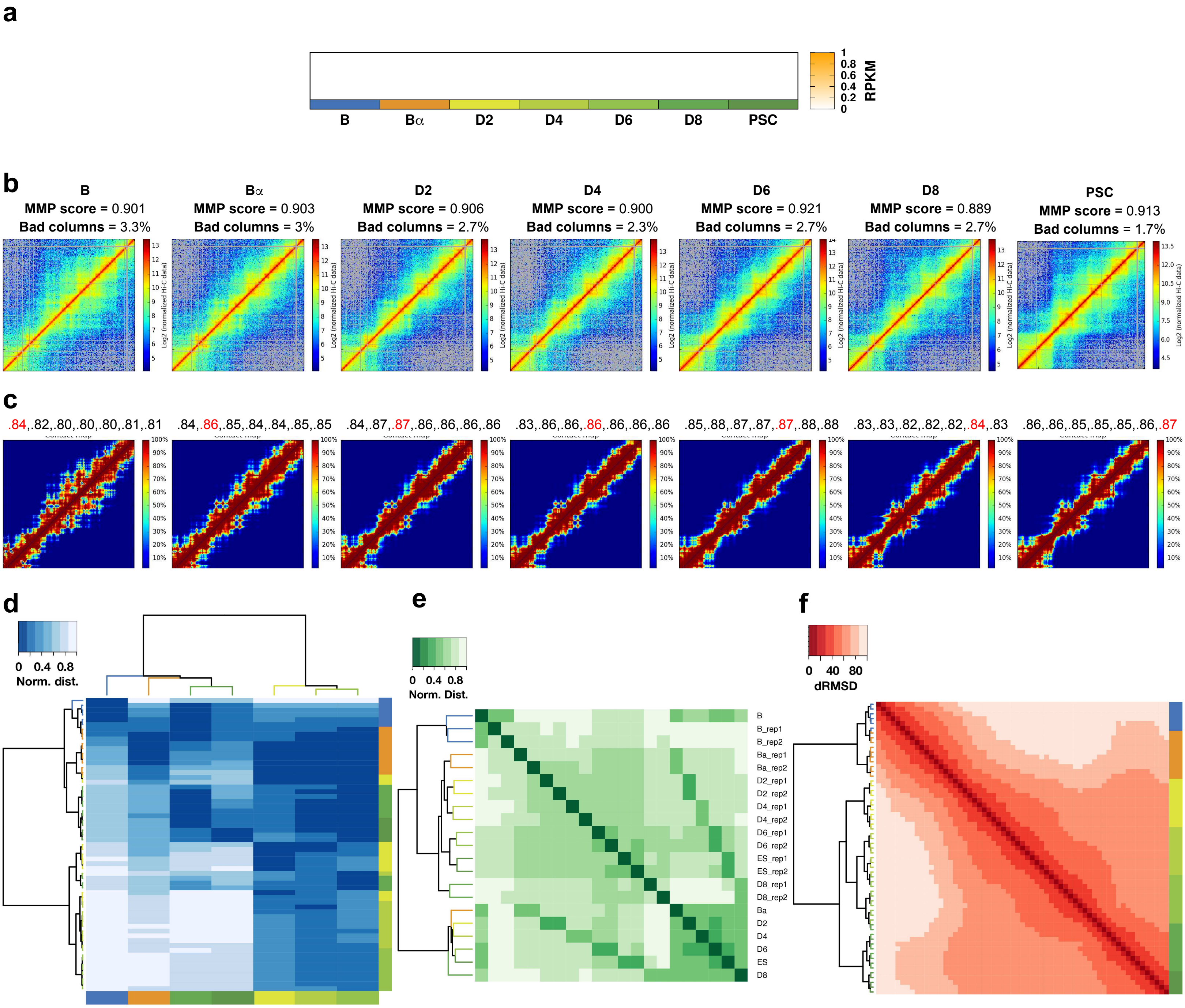

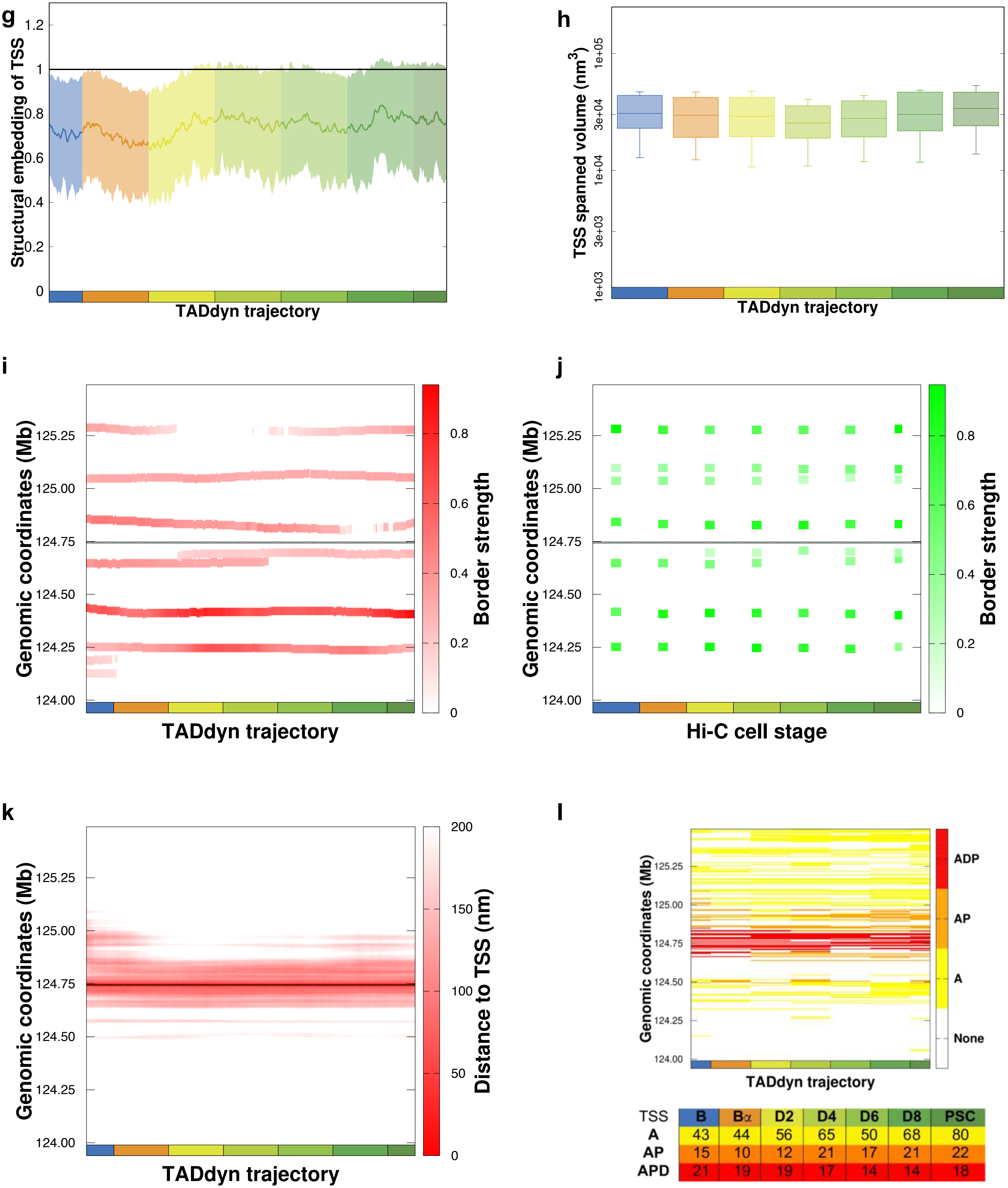

TSS	B	Bα	D2	D4	D6	D8	PSC
A	43	44	56	65	50	68	80
AP	15	10	12	21	17	21	22
APD	21	19	19	17	14	14	18

Supplementary Figure 17. Complete TADdyn analysis of the *Rnu7* simulated locus. The panels present in two pages all the analysis done on the TADdyn models, the Hi-C datasets, the chromatin tracks (ATAC-seq and H3K4me2 peaks), and RNAseq experiments. Specifically, in page 1 we present **(a)** the expression level per cell stage, **(b)** the in-situ Hi-C interaction maps, **(c)** the models' contact maps at dcutoff=200 nm, **(d-f)** the clustering analysis based on models' vs. Hi-C **(d)** and Hi-C vs. Hi-C **(e)** correlations transformed in normalized distances, and models vs. models **(f)** based on structural distance root-mean-squared displacement (dRMSD). On page 2 we show **(g)** the TSS structural embedding along the trajectories where the line represent the average and the colored areas (+/-) the standard deviation, **(h)** the average (over the 100 replicates) of the volume explored by the TSS along the TADdyn trajectories every 5 simulation timesteps at each cell stage represented as boxplots (n=100 data points for B and PSC stages, and n=200 data points for the other cell stages) showing: central line, median; box limits, 75th and 25th percentiles; whiskers, 1.5x interquartile range (outliers not shown), **(i-j)** the domains borders on the models contact maps along the entire trajectory (600 time points) **(i)** and the Hi-C interactions maps at each cell stage (7 time points) **(j)**, **(k)** the heat-map showing the average distance to the TSS of each particle along the TADdyn trajectories, and **(l)** the number of active (A), active-proximal (AP), and active-proximal-domain (APD) particle respect to the TSS. Genome tracks for the annotated genes and regulatory elements (promoters, enhancers, and protein binding sites), and for ATAC-seq, CTCF, and H3K4me2 peaks at each reprogramming stage generated in Stadhouders *et al. Nat. Genet.* **50**, 238-249 (2018) are available here for the *Rnu7* simulated region.

Rpl41: chr10:128548109-128549168. Reverse

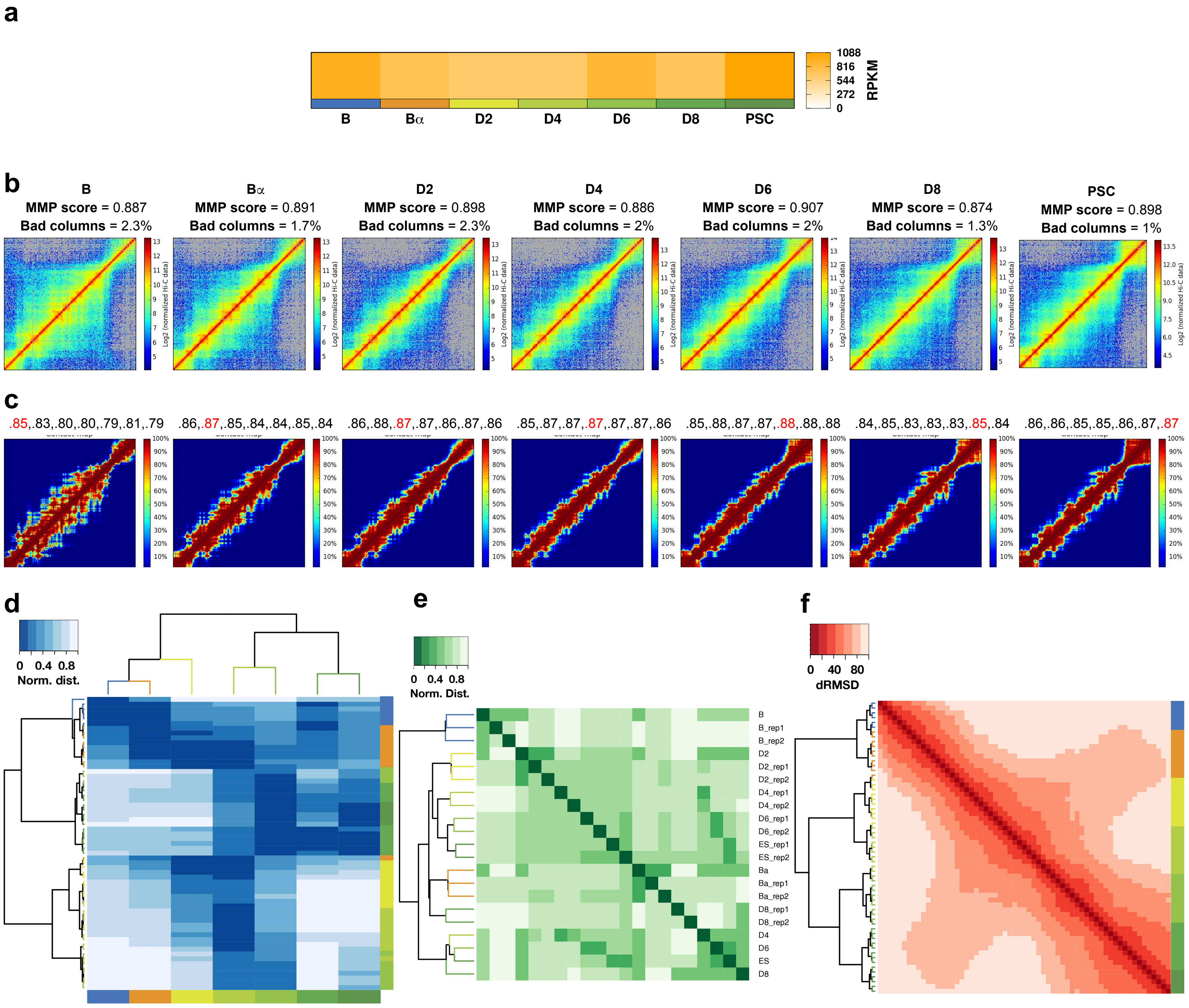

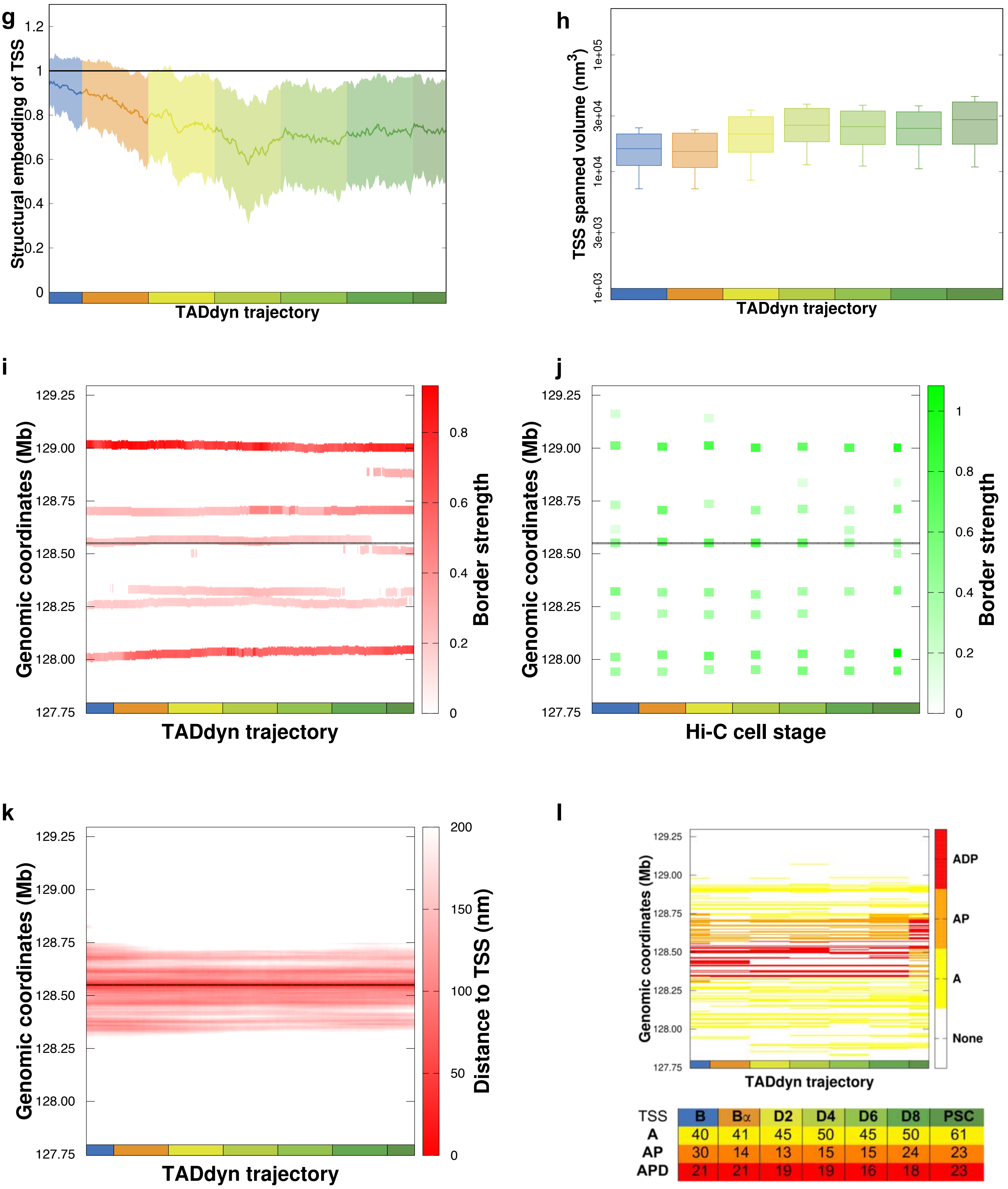

TSS	B	Bα	D2	D4	D6	D8	PSC
A	40	41	45	50	45	50	61
AP	30	14	13	15	15	24	23
APD	21	21	19	19	16	18	23

Supplementary Figure 18. Complete TADdyn analysis of the *Rpl41* simulated locus. The panels present in two pages all the analysis done on the TADdyn models, the Hi-C datasets, the chromatin tracks (ATAC-seq and H3K4me2 peaks), and RNAseq experiments. Specifically, in page 1 we present **(a)** the expression level per cell stage, **(b)** the in-situ Hi-C interaction maps, **(c)** the models' contact maps at dcutoff=200 nm, **(d-f)** the clustering analysis based on models' vs. Hi-C **(d)** and Hi-C vs. Hi-C **(e)** correlations transformed in normalized distances, and models vs. models **(f)** based on structural distance root-mean-squared displacement (dRMSD). On page 2 we show **(g)** the TSS structural embedding along the trajectories where the line represent the average and the colored areas (+/-) the standard deviation, **(h)** the average (over the 100 replicates) of the volume explored by the TSS along the TADdyn trajectories every 5 simulation timesteps at each cell stage represented as boxplots (n=100 data points for B and PSC stages, and n=200 data points for the other cell stages) showing: central line, median; box limits, 75th and 25th percentiles; whiskers, 1.5x interquartile range (outliers not shown), **(i-j)** the domains borders on the models contact maps along the entire trajectory (600 time points) **(i)** and the Hi-C interactions maps at each cell stage (7 time points) **(j)**, **(k)** the heat-map showing the average distance to the TSS of each particle along the TADdyn trajectories, and **(l)** the number of active (A), active-proximal (AP), and active-proximal-domain (APD) particle respect to the TSS. Genome tracks for the annotated genes and regulatory elements (promoters, enhancers, and protein binding sites), and for ATAC-seq, CTCF, and H3K4me2 peaks at each reprogramming stage generated in Stadhouders *et al. Nat. Genet.* **50**, 238-249 (2018) are available here for the *Rpl41* simulated region.

Rps14: chr18:60774595-60778546. Forward

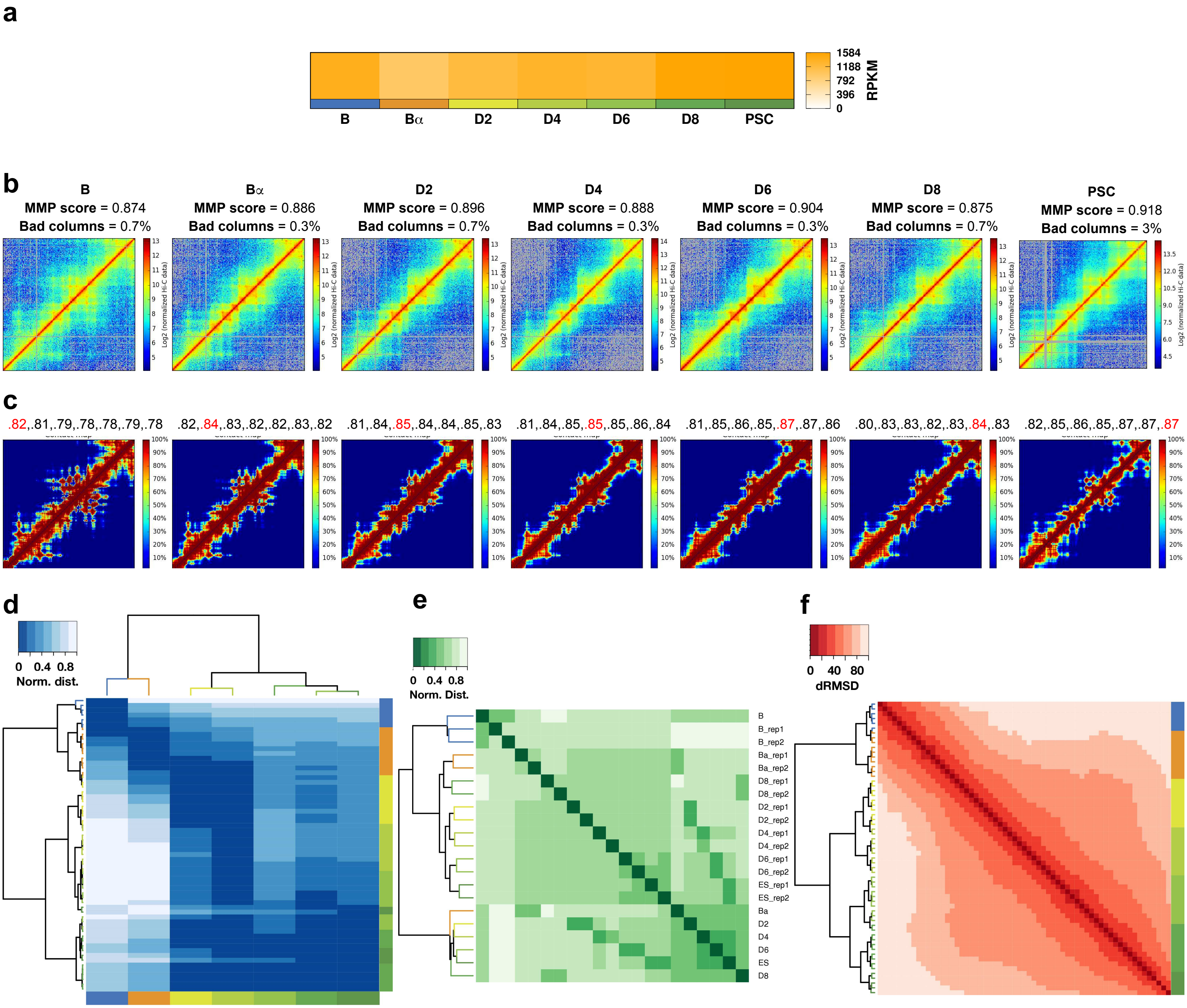

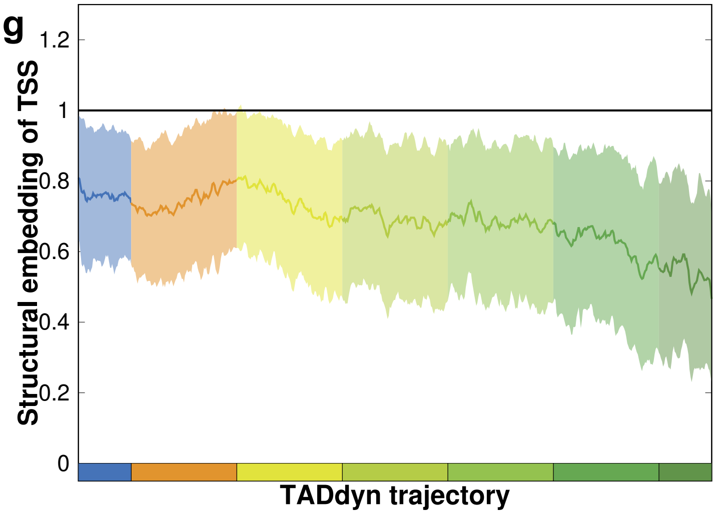

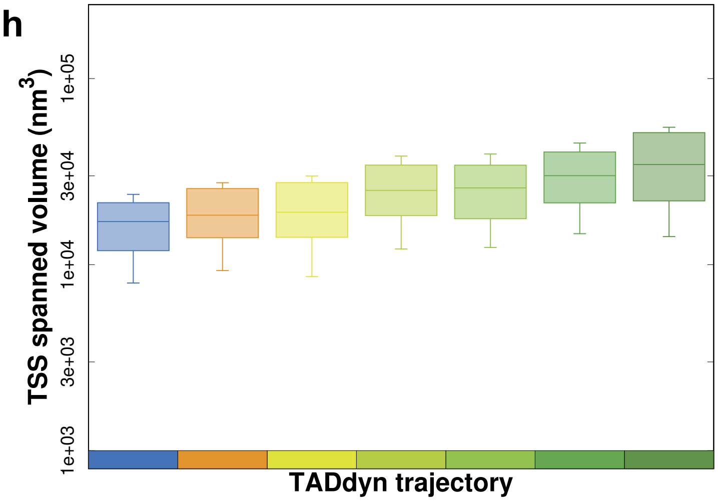

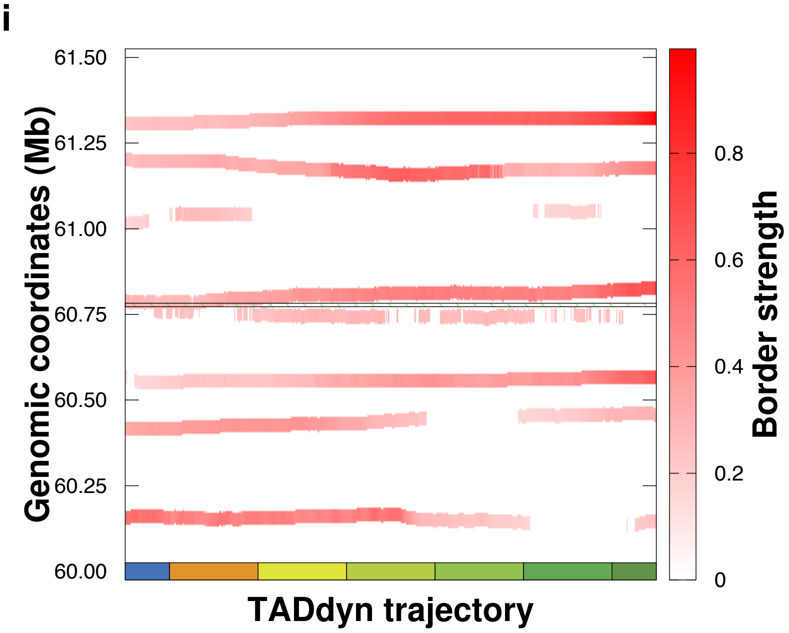

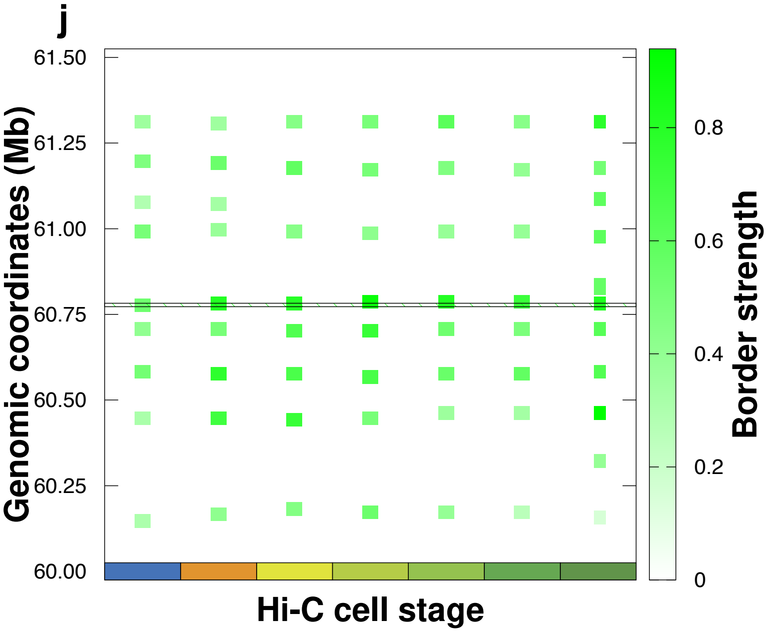

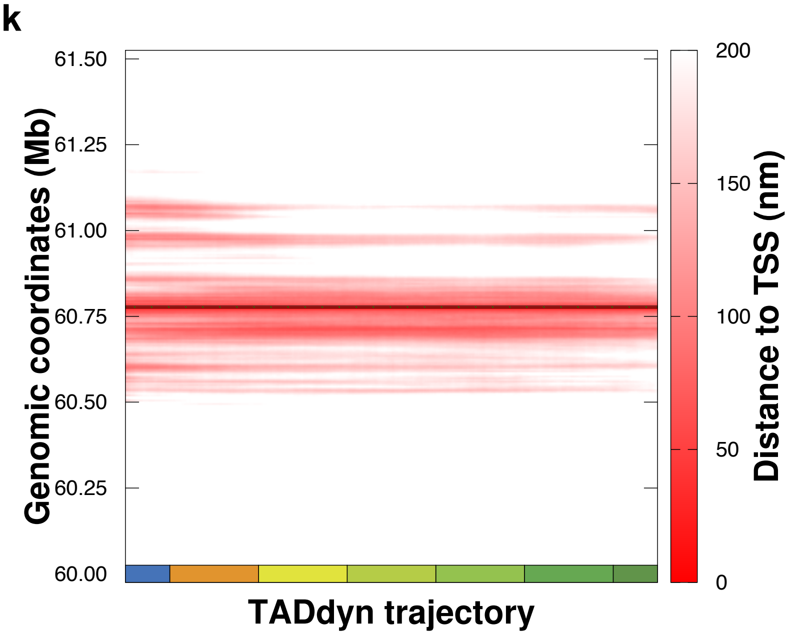

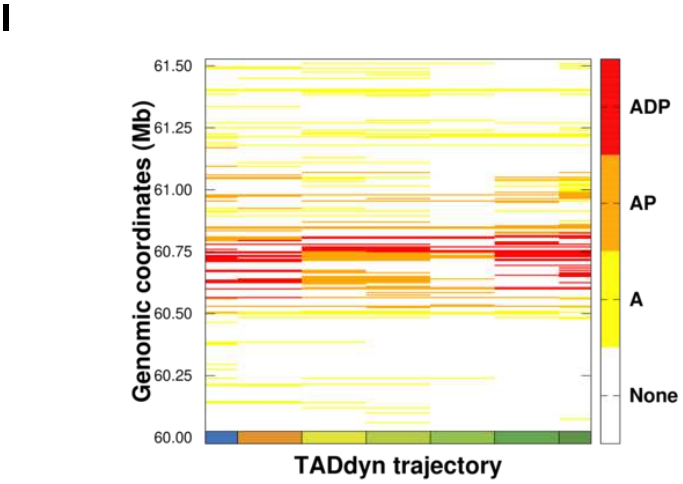

TSS	B	Bα	D2	D4	D6	D8	PSC
A	29	22	32	31	13	14	30
AP	19	13	28	29	15	14	16
APD	17	17	7	8	5	18	24

Supplementary Figure 19. Complete TADdyn analysis of the *Rps14* simulated locus. The panels present in two pages all the analysis done on the TADdyn models, the Hi-C datasets, the chromatin tracks (ATAC-seq and H3K4me2 peaks), and RNAseq experiments. Specifically, in page 1 we present **(a)** the expression level per cell stage, **(b)** the in-situ Hi-C interaction maps, **(c)** the models' contact maps at dcutoff=200 nm, **(d-f)** the clustering analysis based on models' vs. Hi-C **(d)** and Hi-C vs. Hi-C **(e)** correlations transformed in normalized distances, and models vs. models **(f)** based on structural distance root-mean-squared displacement (dRMSD). On page 2 we show **(g)** the TSS structural embedding along the trajectories where the line represent the average and the colored areas (+/-) the standard deviation, **(h)** the average (over the 100 replicates) of the volume explored by the TSS along the TADdyn trajectories every 5 simulation timesteps at each cell stage represented as boxplots (n=100 data points for B and PSC stages, and n=200 data points for the other cell stages) showing: central line, median; box limits, 75th and 25th percentiles; whiskers, 1.5x interquartile range (outliers not shown), **(i-j)** the domains borders on the models contact maps along the entire trajectory (600 time points) **(i)** and the Hi-C interactions maps at each cell stage (7 time points) **(j)**, **(k)** the heat-map showing the average distance to the TSS of each particle along the TADdyn trajectories, and **(l)** the number of active (A), active-proximal (AP), and active-proximal-domain (APD) particle respect to the TSS. Genome tracks for the annotated genes and regulatory elements (promoters, enhancers, and protein binding sites), and for ATAC-seq, CTCF, and H3K4me2 peaks at each reprogramming stage generated in Stadhouders *et al. Nat. Genet.* **50**, 238-249 (2018) are available here for the *Rps14* simulated region.

Rps26: chr10:128624528-128626416. Reverse

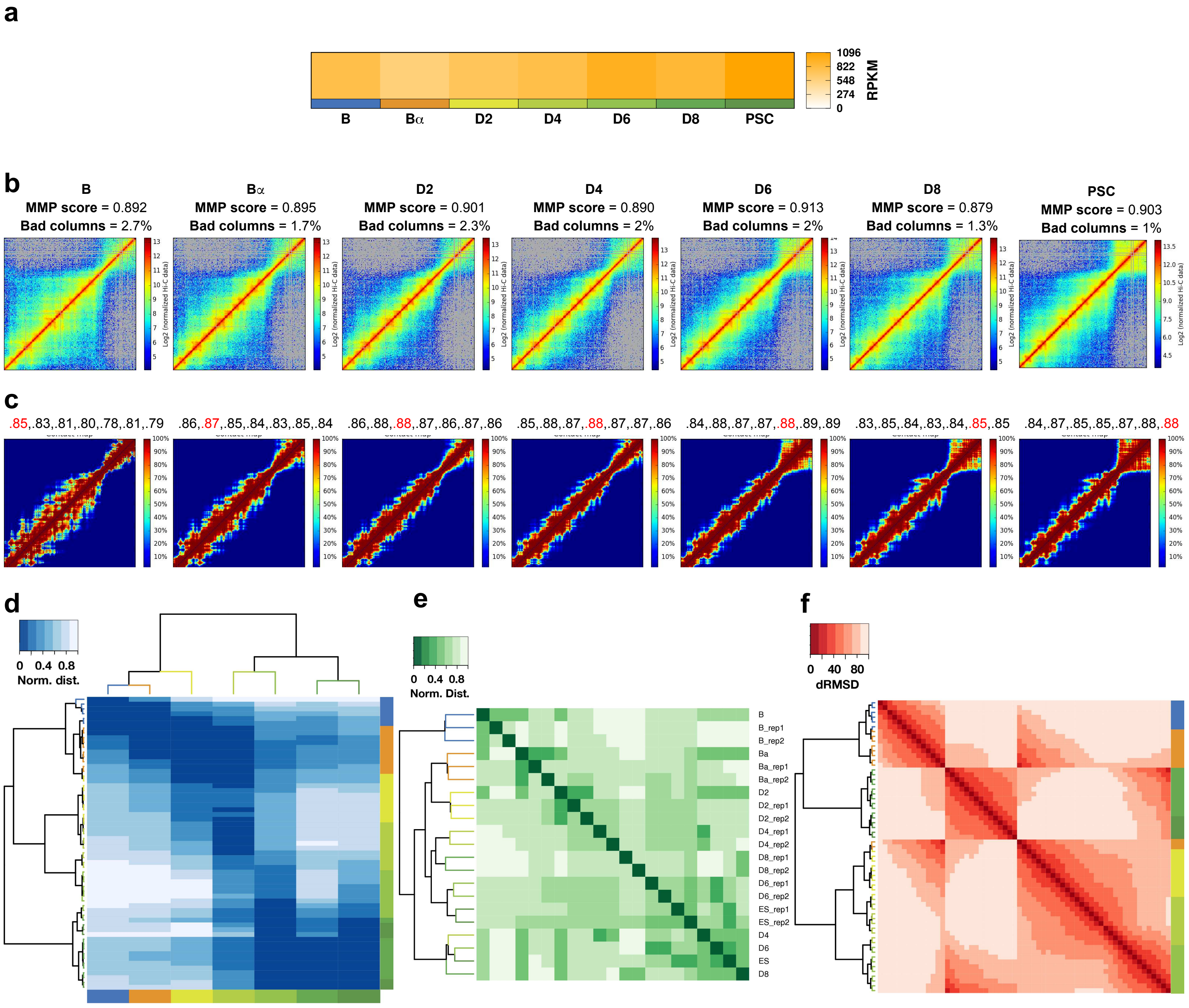

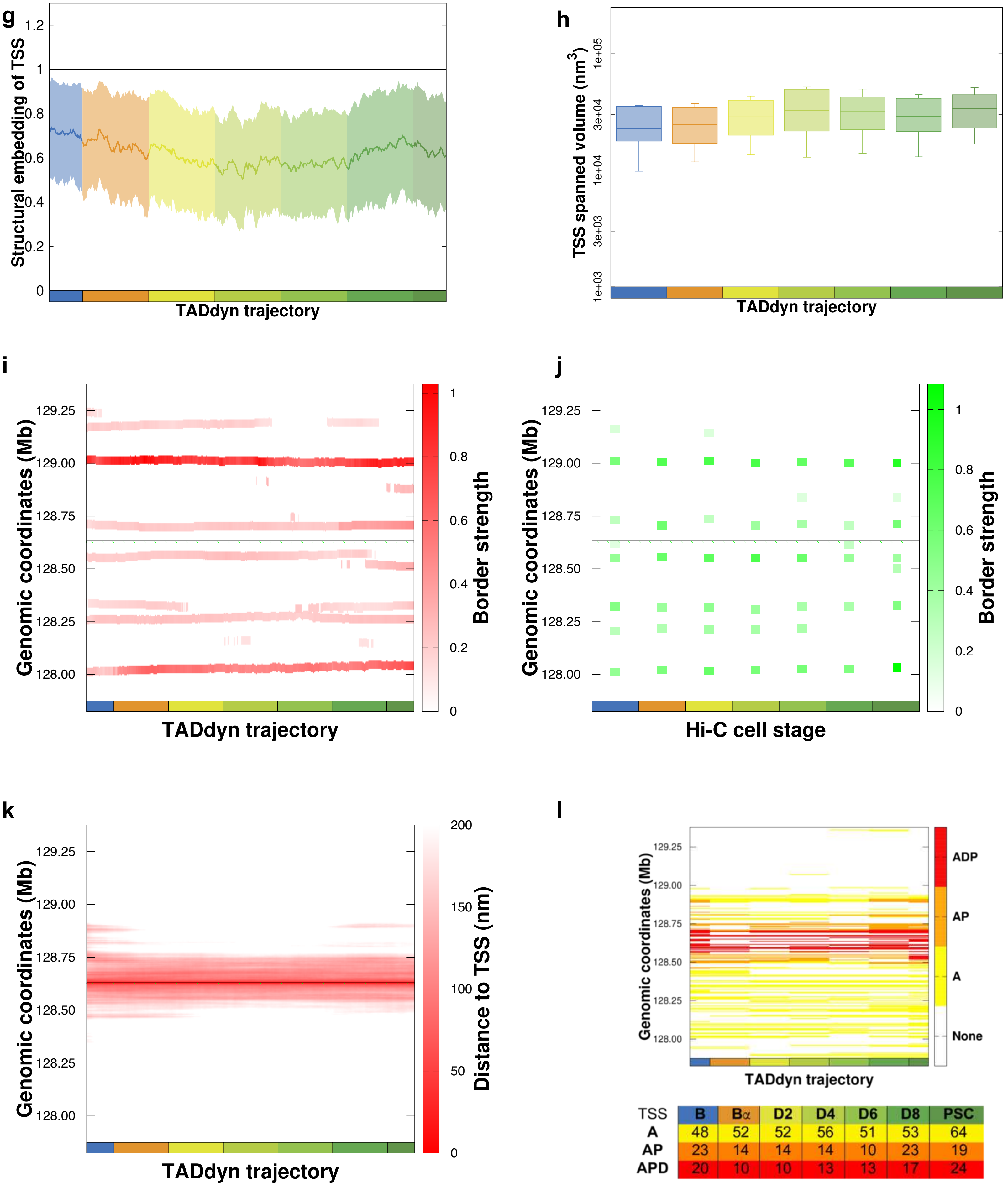

TSS	B	Bα	D2	D4	D6	D8	PSC
A	48	52	52	56	51	53	64
AP	23	14	14	14	10	23	19
APD	20	10	10	13	13	17	24

Supplementary Figure 20. Complete TADdyn analysis of the *Rps26* simulated locus. The panels present in two pages all the analysis done on the TADdyn models, the Hi-C datasets, the chromatin tracks (ATAC-seq and H3K4me2 peaks), and RNAseq experiments. Specifically, in page 1 we present **(a)** the expression level per cell stage, **(b)** the in-situ Hi-C interaction maps, **(c)** the models' contact maps at dcutoff=200 nm, **(d-f)** the clustering analysis based on models' vs. Hi-C **(d)** and Hi-C vs. Hi-C **(e)** correlations transformed in normalized distances, and models vs. models **(f)** based on structural distance root-mean-squared displacement (dRMSD). On page 2 we show **(g)** the TSS structural embedding along the trajectories where the line represent the average and the colored areas (+/-) the standard deviation, **(h)** the average (over the 100 replicates) of the volume explored by the TSS along the TADdyn trajectories every 5 simulation timesteps at each cell stage represented as boxplots (n=100 data points for B and PSC stages, and n=200 data points for the other cell stages) showing: central line, median; box limits, 75th and 25th percentiles; whiskers, 1.5x interquartile range (outliers not shown), **(i-j)** the domains borders on the models contact maps along the entire trajectory (600 time points) **(i)** and the Hi-C interactions maps at each cell stage (7 time points) **(j)**, **(k)** the heat-map showing the average distance to the TSS of each particle along the TADdyn trajectories, and **(l)** the number of active (A), active-proximal (AP), and active-proximal-domain (APD) particle respect to the TSS. Genome tracks for the annotated genes and regulatory elements (promoters, enhancers, and protein binding sites), and for ATAC-seq, CTCF, and H3K4me2 peaks at each reprogramming stage generated in Stadhouders *et al. Nat. Genet.* **50**, 238-249 (2018) are available here for the *Rps26* simulated region.

Sox2: chr3:34649994-34652461. Forward

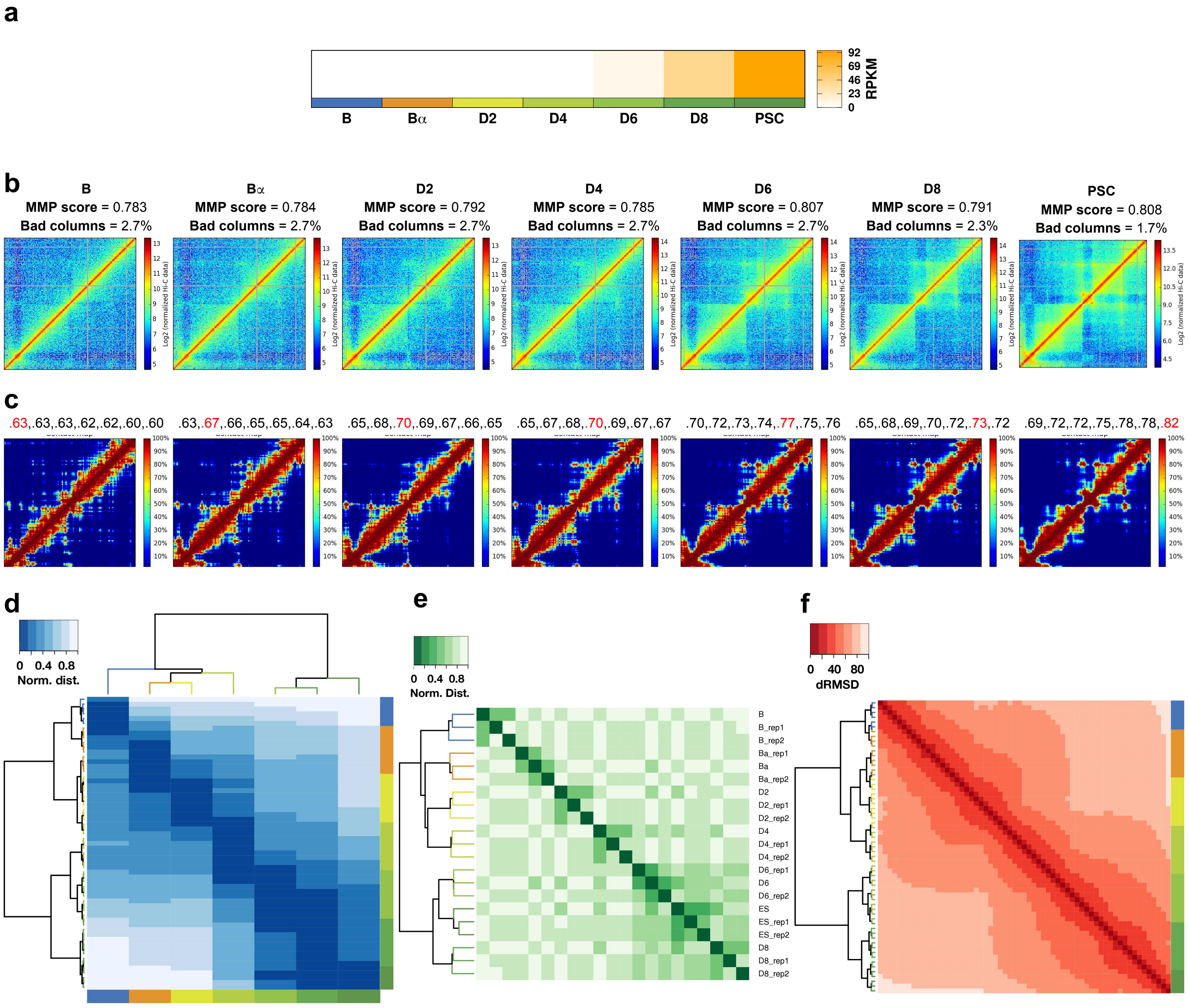

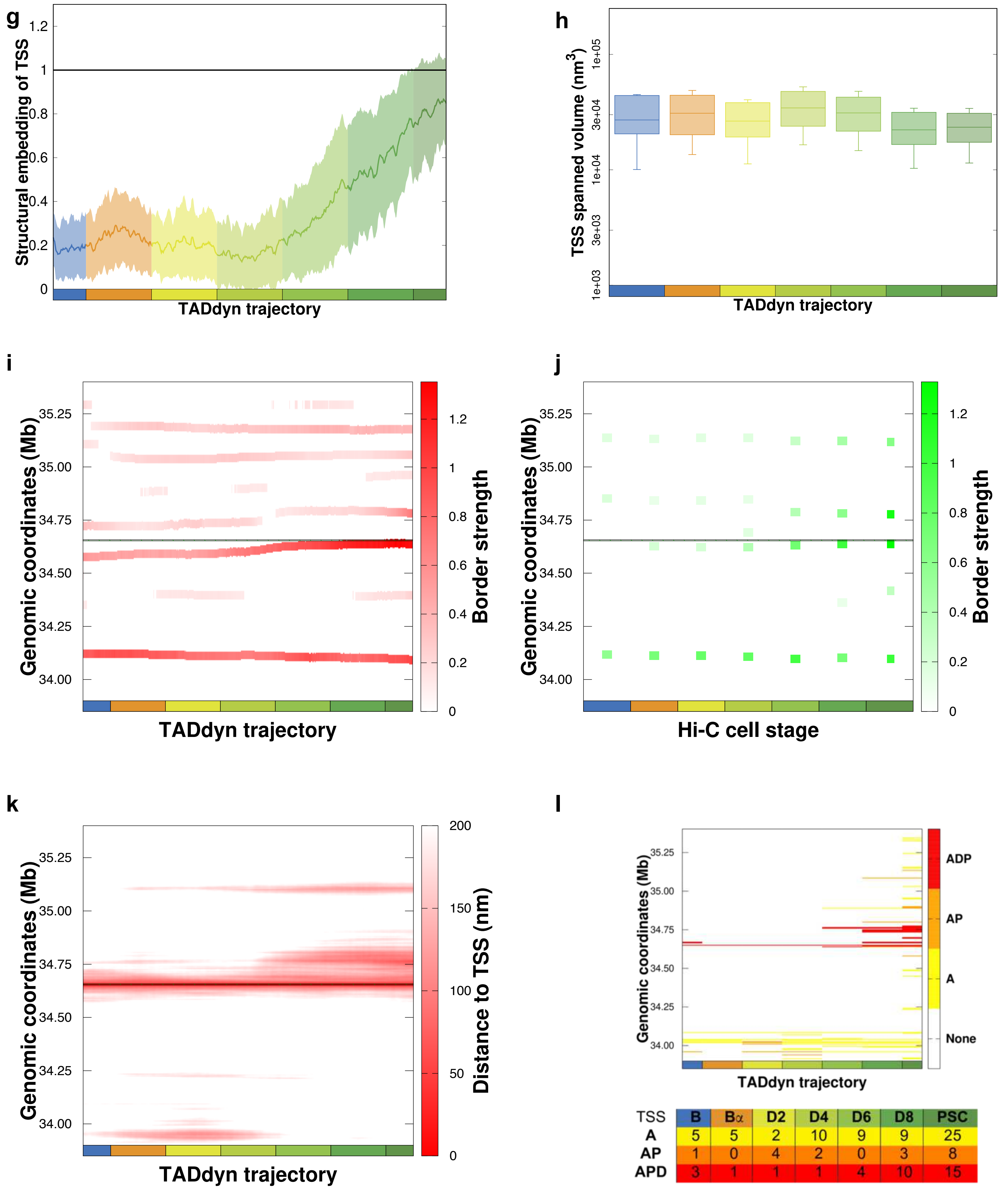

TSS	B	Bα	D2	D4	D6	D8	PSC
A	5	5	2	10	9	9	25
AP	1	0	4	2	0	3	8
APD	3	1	1	1	4	10	15

Supplementary Figure 21. Complete TADdyn analysis of the *Sox2* simulated locus. The panels present in two pages all the analysis done on the TADdyn models, the Hi-C datasets, the chromatin tracks (ATAC-seq and H3K4me2 peaks), and RNAseq experiments. Specifically, in page 1 we present **(a)** the expression level per cell stage, **(b)** the in-situ Hi-C interaction maps, **(c)** the models' contact maps at dcutoff=200 nm, **(d-f)** the clustering analysis based on models' vs. Hi-C **(d)** and Hi-C vs. Hi-C **(e)** correlations transformed in normalized distances, and models vs. models **(f)** based on structural distance root-mean-squared displacement (dRMSD). On page 2 we show **(g)** the TSS structural embedding along the trajectories where the line represent the average and the colored areas (+/-) the standard deviation, **(h)** the average (over the 100 replicates) of the volume explored by the TSS along the TADdyn trajectories every 5 simulation timesteps at each cell stage represented as boxplots (n=100 data points for B and PSC stages, and n=200 data points for the other cell stages) showing: central line, median; box limits, 75th and 25th percentiles; whiskers, 1.5x interquartile range (outliers not shown), **(i-j)** the domains borders on the models contact maps along the entire trajectory (600 time points) **(i)** and the Hi-C interactions maps at each cell stage (7 time points) **(j)**, **(k)** the heat-map showing the average distance to the TSS of each particle along the TADdyn trajectories, and **(l)** the number of active (A), active-proximal (AP), and active-proximal-domain (APD) particle respect to the TSS. Genome tracks for the annotated genes and regulatory elements (promoters, enhancers, and protein binding sites), and for ATAC-seq, CTCF, and H3K4me2 peaks at each reprogramming stage generated in Stadhouders *et al. Nat. Genet.* **50**, 238-249 (2018) are available here for the *Sox2* simulated region.

Tet2: chr3:133463676-133544390. Reverse

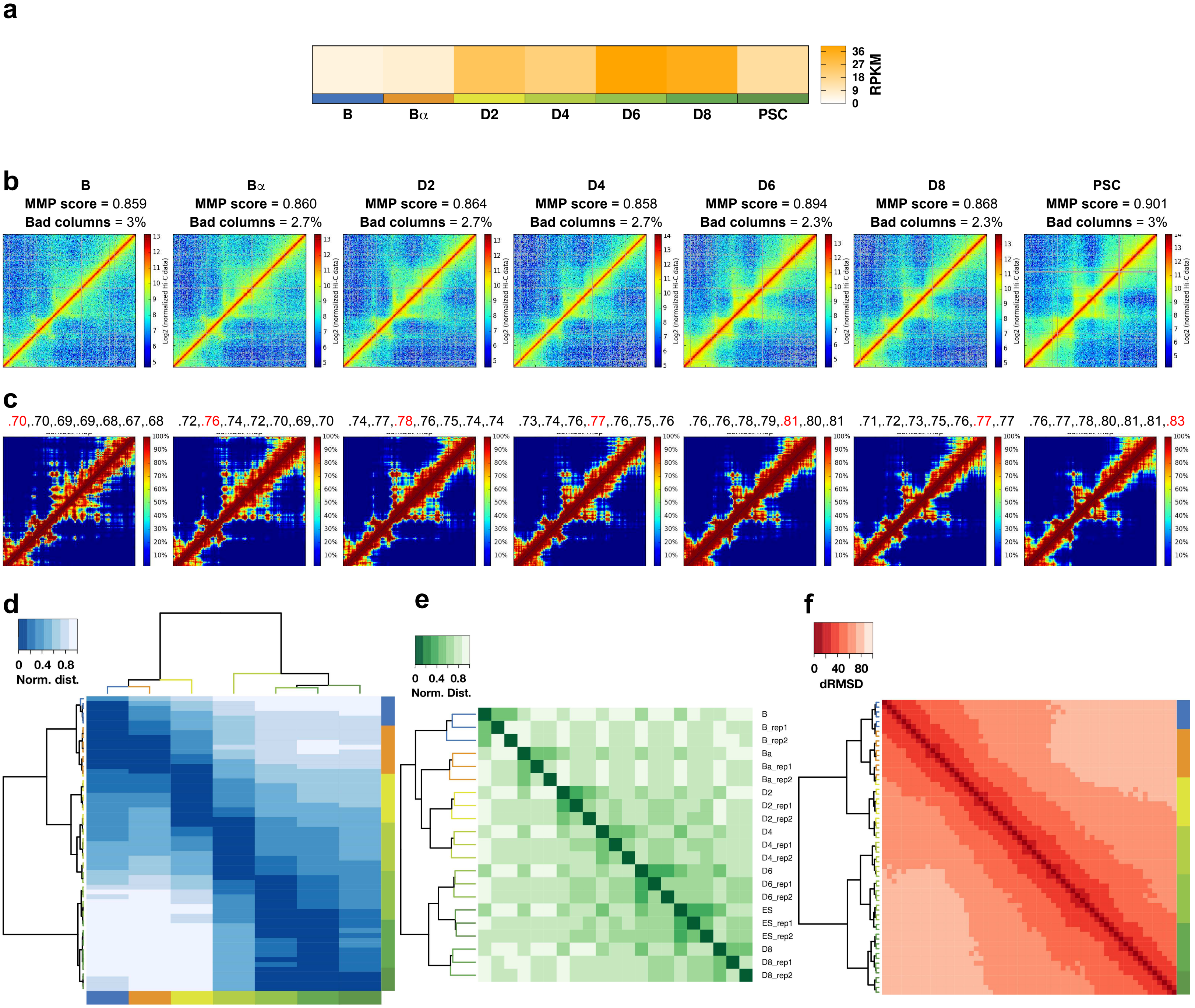

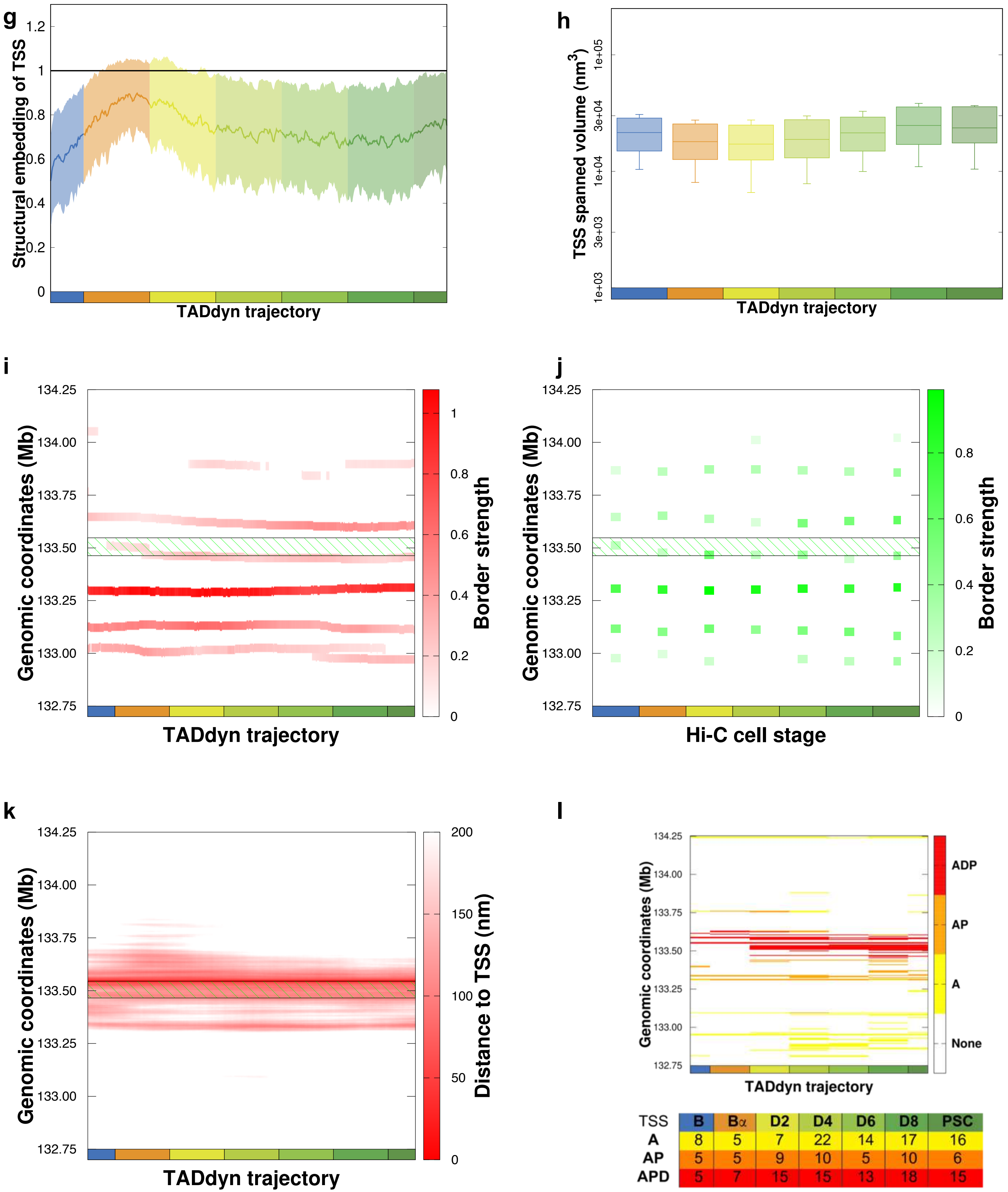

TSS	B	Bα	D2	D4	D6	D8	PSC
A	8	5	7	22	14	17	16
AP	5	5	9	10	5	10	6
APD	5	7	15	15	13	18	15

Supplementary Figure 22. Complete TADdyn analysis of the *Tet2* simulated locus. The panels present in two pages all the analysis done on the TADdyn models, the Hi-C datasets, the chromatin tracks (ATAC-seq and H3K4me2 peaks), and RNAseq experiments. Specifically, in page 1 we present **(a)** the expression level per cell stage, **(b)** the in-situ Hi-C interaction maps, **(c)** the models' contact maps at dcutoff=200 nm, **(d-f)** the clustering analysis based on models' vs. Hi-C **(d)** and Hi-C vs. Hi-C **(e)** correlations transformed in normalized distances, and models vs. models **(f)** based on structural distance root-mean-squared displacement (dRMSD). On page 2 we show **(g)** the TSS structural embedding along the trajectories where the line represent the average and the colored areas (+/-) the standard deviation, **(h)** the average (over the 100 replicates) of the volume explored by the TSS along the TADdyn trajectories every 5 simulation timesteps at each cell stage represented as boxplots (n=100 data points for B and PSC stages, and n=200 data points for the other cell stages) showing: central line, median; box limits, 75th and 25th percentiles; whiskers, 1.5x interquartile range (outliers not shown), **(i-j)** the domains borders on the models contact maps along the entire trajectory (600 time points) **(i)** and the Hi-C interactions maps at each cell stage (7 time points) **(j)**, **(k)** the heat-map showing the average distance to the TSS of each particle along the TADdyn trajectories, and **(l)** the number of active (A), active-proximal (AP), and active-proximal-domain (APD) particle respect to the TSS. Genome tracks for the annotated genes and regulatory elements (promoters, enhancers, and protein binding sites), and for ATAC-seq, CTCF, and H3K4me2 peaks at each reprogramming stage generated in Stadhouders *et al. Nat. Genet.* **50**, 238-249 (2018) are available here for the *Tet2* simulated region.

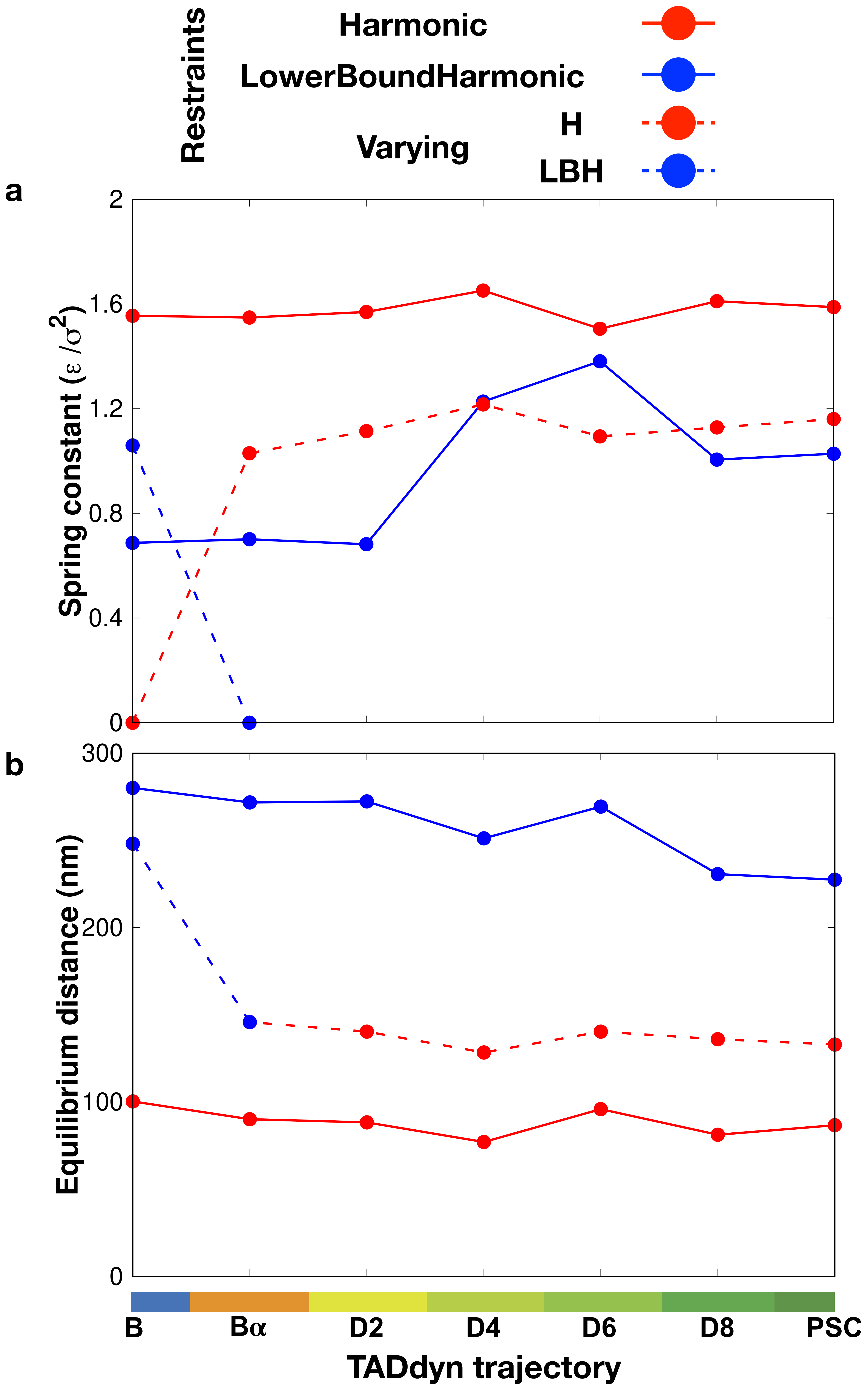

Supplementary Figure 23. Time dependent parameters of the TADdyn dynamic restraints. The TADdyn dynamic restraints are based on the linear interpolations of their two parameters, spring constant **(a)** and equilibrium distance **(b)**, during the trajectory. We show the time variations for 3 pairs of particle in *Sox2* models: (i) the pair (203, 206) interacts with Harmonic restraints (red dots) along the entire trajectory (continuous line), (ii) the pair (28, 201) always (continuous line) with LowerBoundHarmonic restraints (blue dots), and (iii) the particles (111, 123) change their mutual interaction along the trajectory. During the B to B transition the LowerBoundHarmonic (Varying LBH – dashed line and blue dots) vanishes, because its spring constant is brought to zero (panel a), while the Harmonic (Varying H – dashed red line and red dots) takes over and, hence, its spring constant is raised from 0 in B to its value in B.

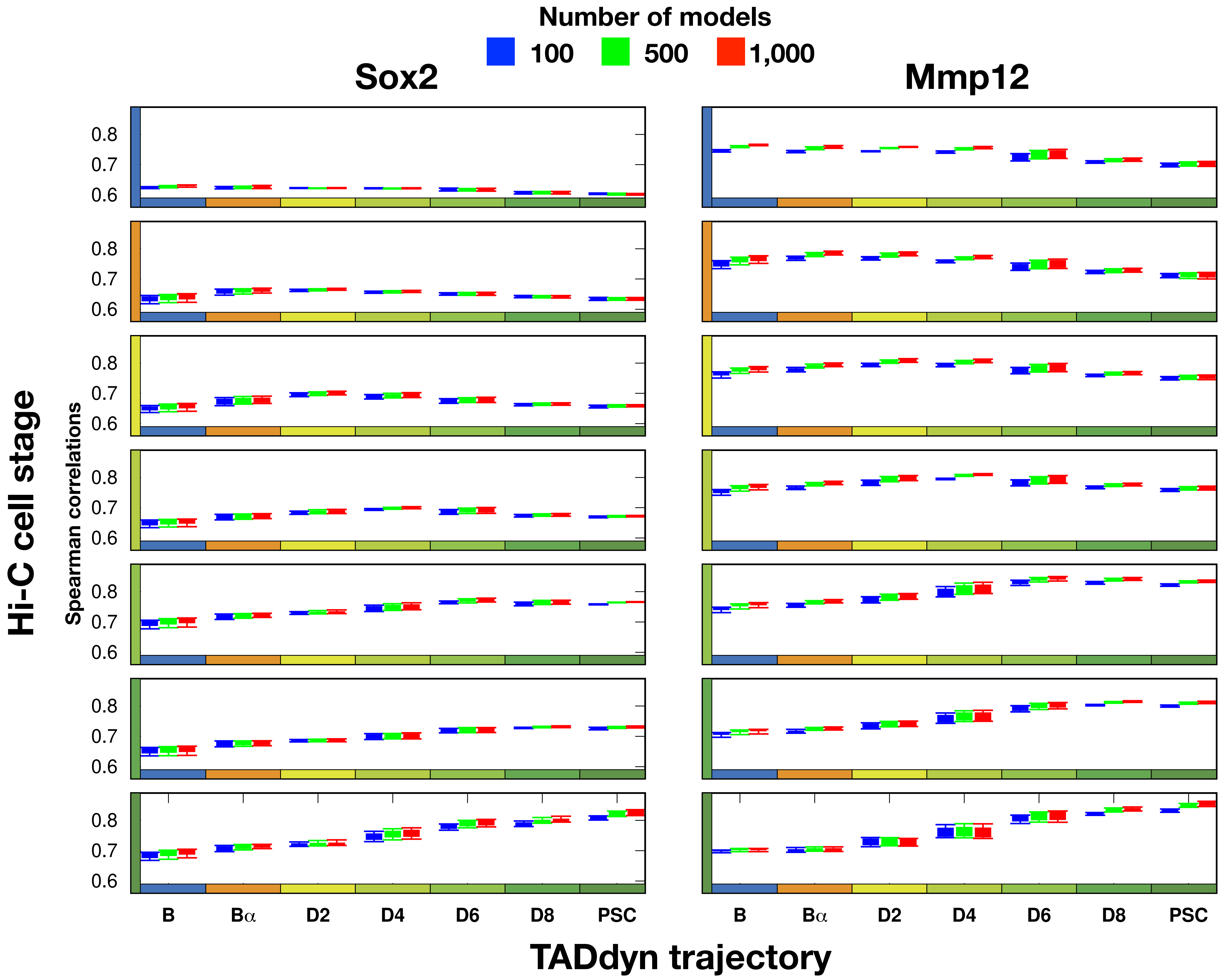

Supplementary Figure 24. Model *vs.* Hi-C contact maps correlation analysis using 100, 500, and 1,000 models of *Sox2* and *Mmp12* loci. The boxplots represent the correlations between the models contact maps along the simulated trajectories (x-axis) and the Hi-C interaction maps at each cell stage (y-axis). Each comparison has been done using an increasing number of models 100 (blue), 500 (green), and 1,000 (red). Although the number of models is varied by a factor of 10, the correlation values are very similar (maximum difference between the averages is 0.02 indicating that already with 100 models the ensemble of TADdyn models is well characterized. Each boxplot shows: central line, median; box limits, 75th and 25th percentiles; whiskers span 95% of the points in the set. The number of data points in each box depends on the cell stage in the x-axis: n=50 for B and PSC and n=100 for the other stages.
