# Supplementary material for: Transcriptional activation during cell reprogramming correlates with the formation of 3D open chromatin hubs
Source: Nat Commun. 2020 May 22;11:2564. doi: 10.1038/s41467-020-16396-1 (PMC7244774; doi:10.1038/s41467-020-16396-1)
Supplement: Supplementary file 2 — Description of Additional Supplementary Files [file 41467_2020_16396_MOESM2_ESM.pdf]

Description of Additional Supplementary Files

File Name: Supplementary Movie 1
Description: CEBPa trajectory. The video displays three distinct synchronized panels. (i) In the model's trajectory (top left) the simulated beads are colored using the red (first particle) - white - blue (last particle) scheme. The TSS particle is represented in black and the promoter in green. If the TSS and the promoter span the same single particle, that is colored in black. (ii) In the expression panel (bottom left) the expression heat-map is spanned by an arrow pointing to the actual simulation time. (iii) The time dependent model's contact maps at dcutoff=200 nm is, finally, shown on the right.

File Name: Supplementary Movie 2
Description: Ebf1 trajectory. The video displays three distinct synchronized panels. (i) In the model's trajectory (top left) the simulated beads are colored using the red (first particle) - white - blue (last particle) scheme. The TSS particle is represented in black and the promoter in green. If the TSS and the promoter span the same single particle, that is colored in black. (ii) In the expression panel (bottom left) the expression heat-map is spanned by an arrow pointing to the actual simulation time. (iii) The time dependent model's contact maps at dcutoff=200 nm is, finally, shown on the right.

File Name: Supplementary Movie 3
Description: Hsp90ab1 trajectory. The video displays three distinct synchronized panels. (i) In the model's trajectory (top left) the simulated beads are colored using the red (first particle) - white - blue (last particle) scheme. The TSS particle is represented in black and the promoter in green. If the TSS and the promoter span the same single particle, that is colored in black. (ii) In the expression panel (bottom left) the expression heat-map is spanned by an arrow pointing to the actual simulation time. (iii) The time dependent model's contact maps at dcutoff=200 nm is, finally, shown on the right.

File Name: Supplementary Movie 4
Description: Lmo7 trajectory. The video displays three distinct synchronized panels. (i) In the model's trajectory (top left) the simulated beads are colored using the red (first particle) - white - blue (last particle) scheme. The TSS particle is represented in black and the promoter in green. If the TSS and the promoter span the same single particle, that is colored in black. (ii) In the expression panel (bottom left) the expression heat-map is spanned by an arrow pointing to the actual simulation time. (iii) The time dependent model's contact maps at dcutoff=200 nm is, finally, shown on the right.

File Name: Supplementary Movie 5
Description: Mmp12 trajectory. The video displays three distinct synchronized panels. (i) In the model's trajectory (top left) the simulated beads are colored using the red (first particle) - white - blue (last particle) scheme. The TSS particle is represented in black and the promoter in green. If the TSS and the promoter span the same single particle, that is colored in black. (ii) In the expression panel (bottom left) the expression heat-map is spanned by an arrow pointing to the actual simulation time. (iii) The time dependent model's contact maps at dcutoff=200 nm is, finally, shown on the right.

File Name: Supplementary Movie 6
Description: Mmp3 trajectory. The video displays three distinct synchronized panels. (i) In the model's trajectory (top left) the simulated beads are colored using the red (first particle) - white - blue (last particle) scheme. The TSS particle is represented in black and the promoter in green. If the TSS and the promoter span the same single particle, that is colored in black. (ii) In the expression panel (bottom left) the expression heat-map is spanned by an arrow pointing to the actual simulation time. (iii) The time dependent model's contact maps at dcutoff=200 nm is, finally, shown on the right.

File Name: Supplementary Movie 7
Description: Nanog trajectory. The video displays three distinct synchronized panels. (i) In the model's trajectory (top left) the simulated beads are colored using the red (first particle) - white - blue (last particle) scheme. The TSS particle is represented in black and the promoter in green. If the TSS and the promoter span the same single particle, that is colored in black. (ii) In the expression panel (bottom left) the expression heat-map is spanned by an arrow pointing to the actual simulation time. (iii) The time dependent model's contact maps at dcutoff=200 nm is, finally, shown on the right.

File Name: Supplementary Movie 8
Description: Neurod6 trajectory. The video displays three distinct synchronized panels. (i) In the model's trajectory (top left) the simulated beads are colored using the red (first particle) - white - blue (last particle) scheme. The TSS particle is represented in black and the promoter in green. If the TSS and the promoter span the same single particle, that is colored in black. (ii) In the expression panel (bottom left) the expression heat-map is spanned by an arrow pointing to the actual simulation time. (iii) The time dependent model's contact maps at dcutoff=200 nm is, finally, shown on the right.

File Name: Supplementary Movie 9
Description: Nos1ap trajectory. The video displays three distinct synchronized panels. (i) In the model's trajectory (top left) the simulated beads are colored using the red (first particle) - white - blue (last particle) scheme. The TSS particle is represented in black and the promoter in green. If the TSS and the promoter span the same single particle, that is colored in black. (ii) In the expression panel (bottom left) the expression heat-map is spanned by an arrow pointing to the actual simulation time. (iii) The time dependent model's contact maps at dcutoff=200 nm is, finally, shown on the right.

File Name: Supplementary Movie 10
Description: Olfr1002 trajectory. The video displays three distinct synchronized panels. (i) In the model's trajectory (top left) the simulated beads are colored using the red (first particle) - white - blue (last particle) scheme. The TSS particle is represented in black and the promoter in green. If the TSS and the promoter span the same single particle, that is colored in black. (ii) In the expression panel (bottom left) the expression heat-map is spanned by an arrow pointing to the actual simulation time. (iii) The time dependent model's contact maps at dcutoff=200 nm is, finally, shown on the right.

File Name: Supplementary Movie 11
Description: Olfr33 trajectory. The video displays three distinct synchronized panels. (i) In the model's trajectory (top left) the simulated beads are colored using the red (first particle) - white - blue (last particle) scheme. The TSS particle is represented in black and the promoter in green. If the TSS and the promoter span the same single particle, that is colored in black. (ii) In the expression panel (bottom left) the expression heat-map is spanned by an arrow pointing to the actual simulation time. (iii) The time dependent model's contact maps at dcutoff=200 nm is, finally, shown on the right.

File Name: Supplementary Movie 12
Description: Ppia trajectory. The video displays three distinct synchronized panels. (i) In the model's trajectory (top left) the simulated beads are colored using the red (first particle) - white - blue (last particle) scheme. The TSS particle is represented in black and the promoter in green. If the TSS and the promoter span the same single particle, that is colored in black. (ii) In the expression panel (bottom left) the expression heat-map is spanned by an arrow pointing to the actual simulation time. (iii) The time dependent model's contact maps at dcutoff=200 nm is, finally, shown on the right.

File Name: Supplementary Movie 13
Description: Ppia trajectory. The video displays three distinct synchronized panels. (i) In the model's trajectory (top left) the simulated beads are colored using the red (first particle) - white - blue (last particle) scheme. The TSS particle is represented in black and the promoter in green. If the TSS and the promoter span the same single particle, that is colored in black. (ii) In the expression panel (bottom left) the expression heat-map is spanned by an arrow pointing to the actual simulation time. (iii) The time dependent model's contact maps at dcutoff=200 nm is, finally, shown on the right.

File Name: Supplementary Movie 14
Description: Rad23a trajectory. The video displays three distinct synchronized panels. (i) In the model's trajectory (top left) the simulated beads are colored using the red (first particle) - white - blue (last particle) scheme. The TSS particle is represented in black and the promoter in green. If the TSS and the promoter span the same single particle, that is colored in black. (ii) In the expression panel (bottom left) the expression heat-map is spanned by an arrow pointing to the actual simulation time. (iii) The time dependent model's contact maps at dcutoff=200 nm is, finally, shown on the right.

File Name: Supplementary Movie 15
Description: Rergl trajectory. The video displays three distinct synchronized panels. (i) In the model's trajectory (top left) the simulated beads are colored using the red (first particle) - white - blue (last particle) scheme. The TSS particle is represented in black and the promoter in green. If the TSS and the promoter span the same single particle, that is colored in black. (ii) In the expression panel (bottom left) the expression heat-map is spanned by an arrow pointing to the actual simulation time. (iii) The time dependent model's contact maps at dcutoff=200 nm is, finally, shown on the right.

File Name: Supplementary Movie 16

Description: Rnu7 trajectory. The video displays three distinct synchronized panels. (i) In the model's trajectory (top left) the simulated beads are colored using the red (first particle) - white - blue (last particle) scheme. The TSS particle is represented in black and the promoter in green. If the TSS and the promoter span the same single particle, that is colored in black. (ii) In the expression panel (bottom left) the expression heat-map is spanned by an arrow pointing to the actual simulation time. (iii) The time dependent model's contact maps at dcutoff=200 nm is, finally, shown on the right.

File Name: Supplementary Movie 17

Description: Rpl41 trajectory. The video displays three distinct synchronized panels. (i) In the model's trajectory (top left) the simulated beads are colored using the red (first particle) - white - blue (last particle) scheme. The TSS particle is represented in black and the promoter in green. If the TSS and the promoter span the same single particle, that is colored in black. (ii) In the expression panel (bottom left) the expression heat-map is spanned by an arrow pointing to the actual simulation time. (iii) The time dependent model's contact maps at dcutoff=200 nm is, finally, shown on the right.

File Name: Supplementary Movie 18

Description: Rps14 trajectory. The video displays three distinct synchronized panels. (i) In the model's trajectory (top left) the simulated beads are colored using the red (first particle) - white - blue (last particle) scheme. The TSS particle is represented in black and the promoter in green. If the TSS and the promoter span the same single particle, that is colored in black. (ii) In the expression panel (bottom left) the expression heat-map is spanned by an arrow pointing to the actual simulation time. (iii) The time dependent model's contact maps at dcutoff=200 nm is, finally, shown on the right.

File Name: Supplementary Movie 19

Description: Rps26 trajectory. The video displays three distinct synchronized panels. (i) In the model's trajectory (top left) the simulated beads are colored using the red (first particle) - white - blue (last particle) scheme. The TSS particle is represented in black and the promoter in green. If the TSS and the promoter span the same single particle, that is colored in black. (ii) In the expression panel (bottom left) the expression heat-map is spanned by an arrow pointing to the actual simulation time. (iii) The time dependent model's contact maps at dcutoff=200 nm is, finally, shown on the right.

File Name: Supplementary Movie 20

Description: Sox2 trajectory. The video displays three distinct synchronized panels. (i) In the model's trajectory (top left) the simulated beads are colored using the red (first particle) - white - blue (last particle) scheme. The TSS particle is represented in black and the promoter in green. If the TSS and the promoter span the same single particle, that is colored in black. (ii) In the expression panel (bottom left) the expression heat-map is spanned by an arrow pointing to the actual simulation time. (iii) The time dependent model's contact maps at dcutoff=200 nm is, finally, shown on the right.

File Name: Supplementary Movie 21

Description: Tet2 trajectory. The video displays three distinct synchronized panels. (i) In the model's trajectory (top left) the simulated beads are colored using the red (first particle) - white - blue (last particle) scheme. The TSS particle is represented in black and the promoter in green. If the TSS and the promoter span the same single particle, that is colored in black. (ii) In the expression panel (bottom left) the expression heat-map is spanned by an arrow pointing to the actual simulation time. (iii) The time dependent model's contact maps at dcutoff=200 nm is, finally, shown on the right.

File Name: Supplementary Data 1

Description: List of the simulated regions. For each of the 21 simulated regions we report in this table the following properties: (1) ID, (2) chromosome, (3-5) the start, stop, and length in base pairs of the locus, (6-8) the start, stop, and length in base pairs of the entire simulated region, (9) the strand forward (+) or reverse (-) in which the gene of interested is expressed, and (10) the index of the particle containing the TSS. Notice that the loci, Mmp3 and Mmp12, are hosted on different parts of the same simulated region called Mmp3_12.

File Name: Supplementary Data 2

Description: Active particle analysis per locus. The detailed account of active (yellow), active-proximal (orange), and active-proximal-domain (red) 5kb- genomic regions is shown per each of the 21 simulated genomic regions.
