# Supplementary material for: Transcriptional activation during cell reprogramming correlates with the formation of 3D open chromatin hubs
Source: Nat Commun. 2020 May 22;11:2564. doi: 10.1038/s41467-020-16396-1 (PMC7244774; doi:10.1038/s41467-020-16396-1)
Supplement: Supplementary file 26 — Reporting Summary [file 41467_2020_16396_MOESM26_ESM.pdf]

natureresearch

Corresponding author(s): Marc Marti-Renom, Marco Di Stefano, Thomas Graf

Last updated by author(s): Apr 13, 2020

# Reporting Summary

Nature Research wishes to improve the reproducibility of the work that we publish. This form provides structure for consistency and transparency in reporting. For further information on Nature Research policies, see Authors & Referees and the Editorial Policy Checklist .

## Statistics

For all statistical analyses, confirm that the following items are present in the figure legend, table legend, main text, or Methods section.

| n/a | Confirmed | |
|---|---|---|
| x | | The exact sample size (*n*) for each experimental group/condition, given as a discrete number and unit of measurement |
| x | | A statement on whether measurements were taken from distinct samples or whether the same sample was measured repeatedly |
| | x | The statistical test(s) used AND whether they are one- or two-sided<br>*Only common tests should be described solely by name; describe more complex techniques in the Methods section.* |
| x | | A description of all covariates tested |
| x | | A description of any assumptions or corrections, such as tests of normality and adjustment for multiple comparisons |
| | x | A full description of the statistical parameters including central tendency (e.g. means) or other basic estimates (e.g. regression coefficient) AND variation (e.g. standard deviation) or associated estimates of uncertainty (e.g. confidence intervals) |
| | x | For null hypothesis testing, the test statistic (e.g. $F$, $t$, $r$) with confidence intervals, effect sizes, degrees of freedom and $P$ value noted<br>*Give P values as exact values whenever suitable.* |
| x | | For Bayesian analysis, information on the choice of priors and Markov chain Monte Carlo settings |
| x | | For hierarchical and complex designs, identification of the appropriate level for tests and full reporting of outcomes |
| x | | Estimates of effect sizes (e.g. Cohen's *d*, Pearson's *r*), indicating how they were calculated |

*Our web collection on statistics for biologists contains articles on many of the points above.*

## Software and code

Policy information about availability of computer code

| | |
|---|---|
| Data collection | TADdyn v1.0 (https://github.com/3DGenomes/TADbit/tree/TADdyn)<br>LAMMPS 31Mar17 |
| Data analysis | Custom scripts used used for Data analysis are available at http://sgt.cnag.cat/3dg/datasets/<br><br>Data analysis:<br>R v3.5.2<br>gnuplot v5.2<br>STAR (Dobin et al., 2013)<br>Bowtie2 (Langmead and Salzberg, 2012)<br>SAMtools (Li et al., 2009)<br>Picard (http://broadinstitute.github.io/picard)<br>HOMER (Heinz et al., 2010)<br>TADbit (Serra et al., 2017)<br>BEDTools (Quinlan and Hall, 2010)<br>ICE (Imakaev et al. 2012)<br><br>3D models visualization:<br>VMD v1.9.3<br>Chimera v1.13.1 |

For manuscripts utilizing custom algorithms or software that are central to the research but not yet described in published literature, software must be made available to editors/reviewers. We strongly encourage code deposition in a community repository (e.g. GitHub). See the Nature Research guidelines for submitting code & software for further information.

## Data

Policy information about availability of data

All manuscripts must include a data availability statement. This statement should provide the following information, where applicable:
- Accession codes, unique identifiers, or web links for publicly available datasets
- A list of figures that have associated raw data
- A description of any restrictions on data availability

In-situ Hi-C, ChIP-seq (H3K4me2 histone mark), ATAC-seq, and RNA-seq datasets were obtained from Gene Expression Omnibus (GEO) at the accession number GSE96611.

# Field-specific reporting

Please select the one below that is the best fit for your research. If you are not sure, read the appropriate sections before making your selection.

- [x] Life sciences
- [ ] Behavioural & social sciences
- [ ] Ecological, evolutionary & environmental sciences

For a reference copy of the document with all sections, see nature.com/documents/nr-reporting-summary-flat.pdf

# Life sciences study design

All studies must disclose on these points even when the disclosure is negative.

| | |
|---|---|
| Sample size | No statistical methods were used to determine sample sizes. |
| Data exclusions | No data exclusions were applied in this study. |
| Replication | No attempts to reproduce results failed. |
| Randomization | Randomization is not relevant to this study because no comparisons between experimental groups were made. |
| Blinding | Blinding was not relevant to this study because all metrics were derived from absolute quantitative methods without human subjectivity |

# Reporting for specific materials, systems and methods

We require information from authors about some types of materials, experimental systems and methods used in many studies. Here, indicate whether each material, system or method listed is relevant to your study. If you are not sure if a list item applies to your research, read the appropriate section before selecting a response.

## Materials & experimental systems

| n/a | Involved in the study | |
|---|---|---|
| x | | Antibodies |
| x | | Eukaryotic cell lines |
| x | | Palaeontology |
| x | | Animals and other organisms |
| x | | Human research participants |
| x | | Clinical data |

## Methods

| n/a | Involved in the study | |
|---|---|---|
| x | | ChIP-seq |
| x | | Flow cytometry |
| x | | MRI-based neuroimaging |
